# Investigating glycemic potential of rice by unraveling compositional variations in mature grain and starch mobilization patterns during seed germination

**DOI:** 10.1038/s41598-017-06026-0

**Published:** 2017-07-19

**Authors:** Maria Krishna de Guzman, Sabiha Parween, Vito M. Butardo, Crisline Mae Alhambra, Roslen Anacleto, Christiane Seiler, Anthony R. Bird, Chung-Ping Chow, Nese Sreenivasulu

**Affiliations:** 1 Grain Quality and Nutrition Center, Plant Breeding Division, International Rice Research Institute, Los Baños, Laguna 4030 Philippines; 2 The Leibniz Institute of Plant Genetics and Crop Plant Research (IPK), Gatersleben, Germany; 3 CSIRO Health and Biosecurity, Kintore Ave, Adelaide, SA 5000 Australia; 4 Waters Pacific Pte Ltd, Singapore Science Park II, Singapore, 117528 Singapore; 5 ARC Industrial Transformation Training Centre for Functional Grains (FGC), Graham Centre for Agricultural Innovation, Charles Sturt University, Wagga, Wagga NSW 2650 Australia; 6 Genedata AG, Basel, CH-4053 Switzerland

## Abstract

Rice lines with slower starch digestibility provide opportunities in mitigating the global rise in type II diabetes and related non-communicable diseases. However, screening for low glycemic index (GI) in rice breeding programs is not possible due to time and cost constraints. This study evaluated the feasibility of using *in vitro* cooked grain amylolysis, starch mobilization patterns during seed germination, and variation in starch structure and composition in the mature seed to differentiate patterns of starch digestibility. Mobilization patterns of total starch, resistant starch, amylose and amylopectin chains, and free sugars during seed germination revealed that the process is analogous to digestion in the human gastrointestinal tract. The combination of these biochemical markers can be used as an alternative measure to predict GI. Additionally, transcriptome analysis of stored mRNA transcripts in high and low GI lines detected differences in starch metabolism and confirmed the importance of seed storage pathways in influencing digestibility. Pathway analyses supported by metabolomics data revealed that resistant starch, cell wall non-starch polysaccharides and flavonoids potentially contribute to slower digestibility. These new insights can guide precision breeding programs to produce low GI rice with acceptable cooking quality to help mitigate the burden of diet-associated lifestyle diseases.

## Introduction

The world is facing a global rise in deaths related to non-communicable diseases (NCDs) such as diabetes mellitus, obesity and cardiovascular ailments. An estimated 630 million people in developing and developed countries are projected to contract diabetes by the year 2030^1^. Likewise, the global incidence of obesity has increased two-fold, affecting even the younger population^2^. In 2015, 31% of all global deaths were due to cardiovascular disease^3^. It is currently the leading cause of death in the world. These alarming health statistics call for a focus on diet-based nutritional intervention across the entire socioeconomic consumer spectrum. Thus, crop breeding programs have to be reviewed with the aim of diversifying the digestibility of starchy products. For instance, staple cereals with slowly digestible grains can be developed by elevating the proportion of amylose and resistant starch (RS) fractions. This is usually accomplished by increasing the proportion of amylose or long chain amylopectin to reduce glycemic response^4–7^.

Glycemic index (GI) is defined as the area under the blood glucose response curve that is measured two hours after a fixed amount of available carbohydrate is consumed compared to a control food, which is either white bread or glucose solution^8^. It has been used as the key clinical marker in characterizing starchy food and assessing dietary carbohydrate quality^9^. GI is influenced primarily by the structure and composition of starch^10^. The most reliable and generally-accepted method of measuring GI is through *in vivo* clinical assays that involve human volunteers as subjects. However, this approach is expensive and is highly affected by the genetics, physiology and metabolism of the volunteers. Aside from issues of intra-individual variability and inter-individual reproducibility, methodical variations in determining the GI response among laboratories exist^11–13^. Consequently, proxy measures for the *in vitro* determination of GI were developed^14^ and gained wide acceptance. These methods use enzymatic hydrolysis that closely mimics starch digestion in humans^15^.

The digestion of starch in the human gastrointestinal tract involves multiple interdependent steps which make the development of *in vitro* simulation challenging. Starch is first broken down by mechanical disruption during chewing, followed by mixing with salivary α-amylases in the mouth^16, 17^. The process continues in the stomach and small intestine where pancreatic α-amylase degrades starch to oligosaccharides which are converted to glucose by the action of maltase, isomaltase and glucoamylase. If digestion is sufficiently slow such that starch survives until the end of the small intestine, the residual material is considered as RS. The RS enters the colon where it is fermented in a manner analogous to that of dietary fiber which benefits gut health^18–20^.

Automated *in vitro* systems which mimic digestion along the human gastrointestinal tract are available^14, 21^ but are still low throughput. Interestingly, starch digestion in animals is comparable to starch mobilization during seed germination in plants. During germination, starch in the grain reserve is accessed and mobilized by the action of endohydrolases and glucosidases which act on the α-1,4 glucan linkages of amylose to release simple sugars^22^. In the case of amylopectin, an additional enzyme specific for α-1,6 cleavage (debranching enzyme) is needed to obtain glucose from liberated maltodextrins^22^. In comparison, carbohydrate-active enzymes are also utilized to breakdown starch during the *in vivo* or *in vitro* determination of GI. The amylolysis of starch granules during rice germination is the major metabolic route for starch utilization in the endosperm. The peak of starch breakdown in germinating rice seeds happens after four days of imbibition which is accompanied by the elevation of α-amylase activity^23^. The combined action of α-amylases, debranching enzymes and α-glucosidases releases glucose which is converted to sucrose in the scutellum and transported to the embryo to promote seed germination and radicle growth^24^. Hence, using germination assay to predict the digestibility of the grain potentially offers a more affordable alternative to *in vitro* amylolysis assays because it relies on endogenous amylolytic enzymes in the rice kernel.

In this study, the GI values of 27 rice genotypes from different breeding materials, including a high amylose mutant IR36 amylose extender (IR36ae) were predicted using an *in vitro* method for predicting GI^5, 25^. Five selected rice varieties that significantly differ in their GI responses were subjected to more detailed starch digestibility kinetic assays. The proportions of total starch, RS, amylose and amylopectin were measured in mature seed and at different time points to determine starch mobilization patterns during germination. In addition, genome-wide transcriptome and metabolome analyses using the mature grains of varieties with low, intermediate, and high GI values were conducted to identify key regulatory genes and target metabolites that influence the pathways of storage products and their digestibility.

## Results

### The proportion of amylose and amylopectin is correlated not just with estimated GI but also with the kinetics of starch digestibility

**Figure 1:**
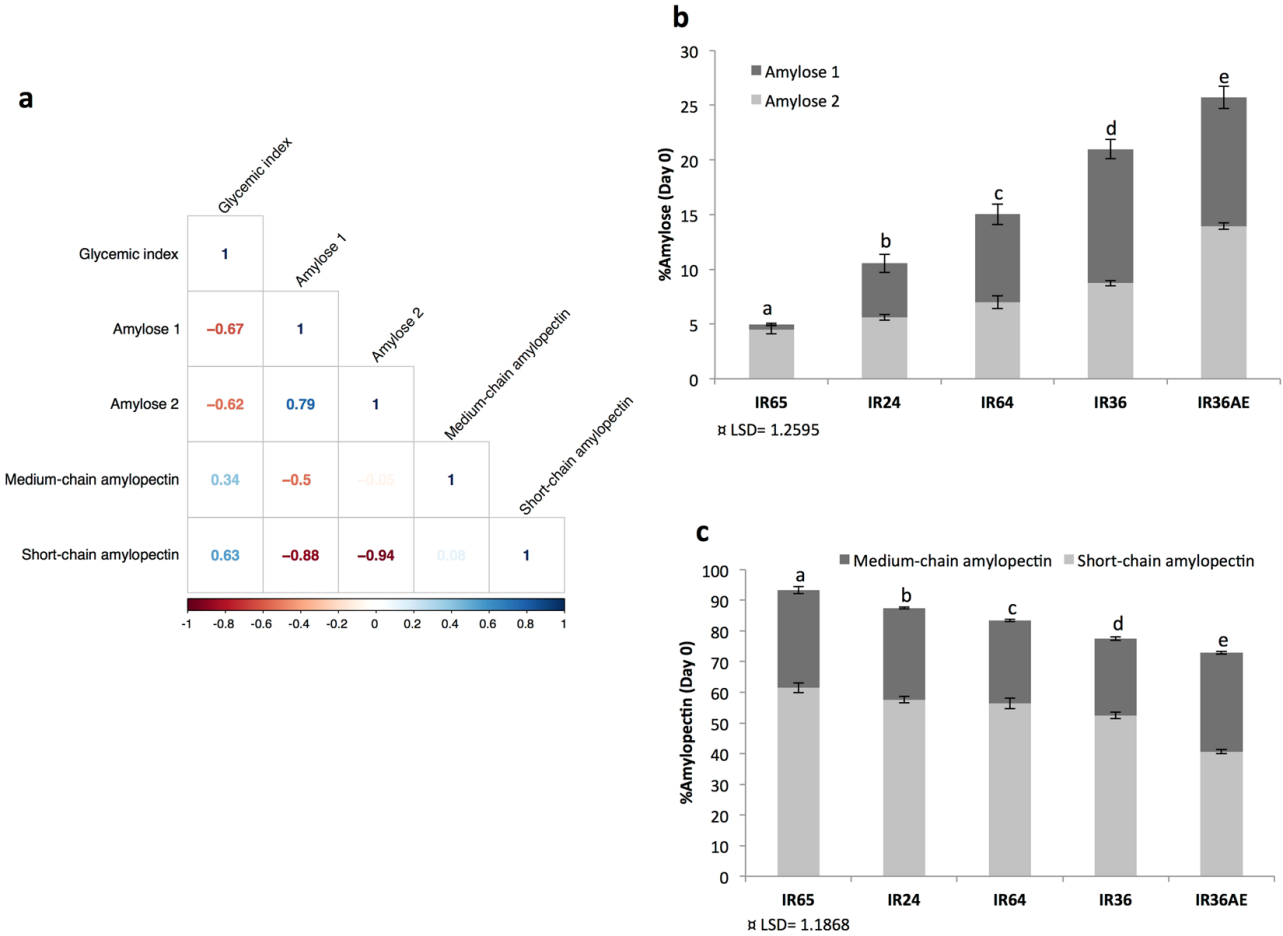
Glycemic index and starch regions. (**a**) Correlation between the starch regions in the mature grains of 27 breeding lines with estimated glycemic indeces and amounts of (**b**) amylose and (**c**) amylopectin fractions before the start of imbibition of five selected rice lines.

The predicted GI values of the 27 breeding lines ranged from 56 to 100. IR36ae (control) had the highest levels of amylose and RS and also the lowest GI value, which is corroborated by previous studies^4, 7^. The correlation between GI, amylose and amylopectin was investigated by dividing the starch into four regions based on previously reported methods^5, 7^. Carbohydrate-containing foods like rice contain starch fractions that are digested at different rates^26, 27^. The relationship between GI and these starch fractions was investigated by establishing correlations with percent amylose 1 (AM1, long chain amylose), amylose 2 (AM2, long chain amylopectin behaving like amylose), medium chain amylopectin (MCAP) and short chain amylopectin (SCAP). Based on correlation analysis (Fig. 1a), AM1 (R = −0.67) and AM2 (R = −0.62) were inversely correlated to GI. On the other hand, the SCAP fraction had a positive correlation with a value of 0.63. MCAP, however, had low correlation with GI. Thus, higher levels of AM1 and AM2 lead to reduced GI. Actual results show that the control sample with the lowest GI (IR36ae) has a higher percentage of AM1 and the lowest percentage of SCAP compared to other samples (Fig. 1b and c).

**Figure 2:**
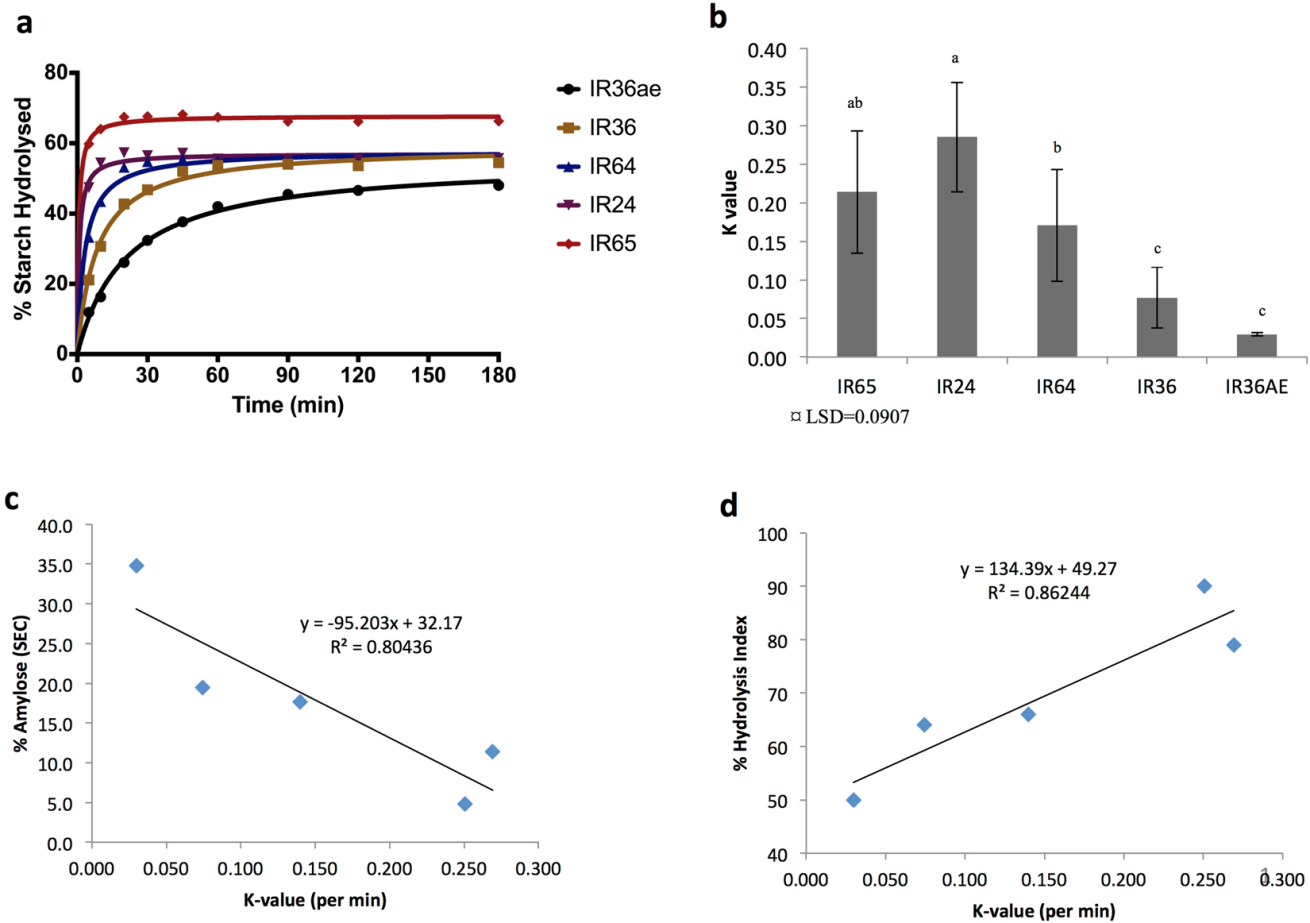
Digestion kinetics of five contrasting lines. (**a**) *In vitro* starch digestion by cooked grain amylolysis and derived (**b**) digestion rate constants (*k value)* using the logarithm of slope (LOS) method. The kinetics of starch digestion is described by comparing the *k value* with (**c**) percent amylose and (**d**) predicted GI.

To further determine the correlation between starch structure and digestibility, five contrasting lines were subjected to detailed analyses. These lines represent waxy (IR65), low (IR24), intermediate (IR64), high (IR36) and very high (IR36ae) amylose classes (Supplementary Table 1). Characterizing the digestibility kinetics based on the extent of starch hydrolyzed (%) found that the first hour is the most crucial in differentiating digestibility (Fig. 2a) and that, by the second hour, starch hydrolysis is complete as indicated by a plateau in the kinetics. This starch hydrolysis kinetics is analogous to clinical GI determination where blood glucose is measured for a period of two hours. The digestion curves suggest that the varieties IR65 and IR24, which have no or low proportion of AM1, respectively, exhibit higher digestibility. In these high GI genotypes, starch hydrolysis was rapid as indicated by a steep curve within the first 5 minutes of amylolysis and nearly 60–70% starch was hydrolyzed within 20 minutes. This corresponds to rapidly digestible starch (RDS) fraction which can significantly elevate blood glucose immediately upon consumption. In contrast, the digestion of IR64 and IR36, which have higher proportion of AM1 took 30 minutes to reach the 60% starch hydrolysis mark, corresponding to slowly digestible starch (SDS) fraction which can provide slow and steady supply of glucose. The least digestible sample was IR36ae, in which only 56% starch was hydrolyzed after 180 minutes (Fig. 2a). Undigested starch fraction in IR36ae corresponds to RS which act as dietary fibre in the colon. Using the digestion curves, digestibility rate constants (*k value*) were estimated using the natural logarithm of slope (LOS) method^10, 28^. As shown in Fig. 2b, *k value* is directly related to digestibility. The rates of starch hydrolysis, based on *k* value routinely used to estimate the GI of starchy foods^29^, revealed that digestibility decreases with increasing amylose content (Fig. 2c). The *k* value is also shown to be strongly correlated with estimated GI (Fig. 2d). Over all, cooked grain amylolysis assessed through a variation in amylose and amylopectin fractions by size exclusion chromatography (SEC) and by an independent *in vitro* digestibility method provides a good estimate of GI.

### Starch compositional and structural variation in the mature grain together with starch reserve mobilization during seed germination reflects the digestibility of rice varieties

**Figure 3:**
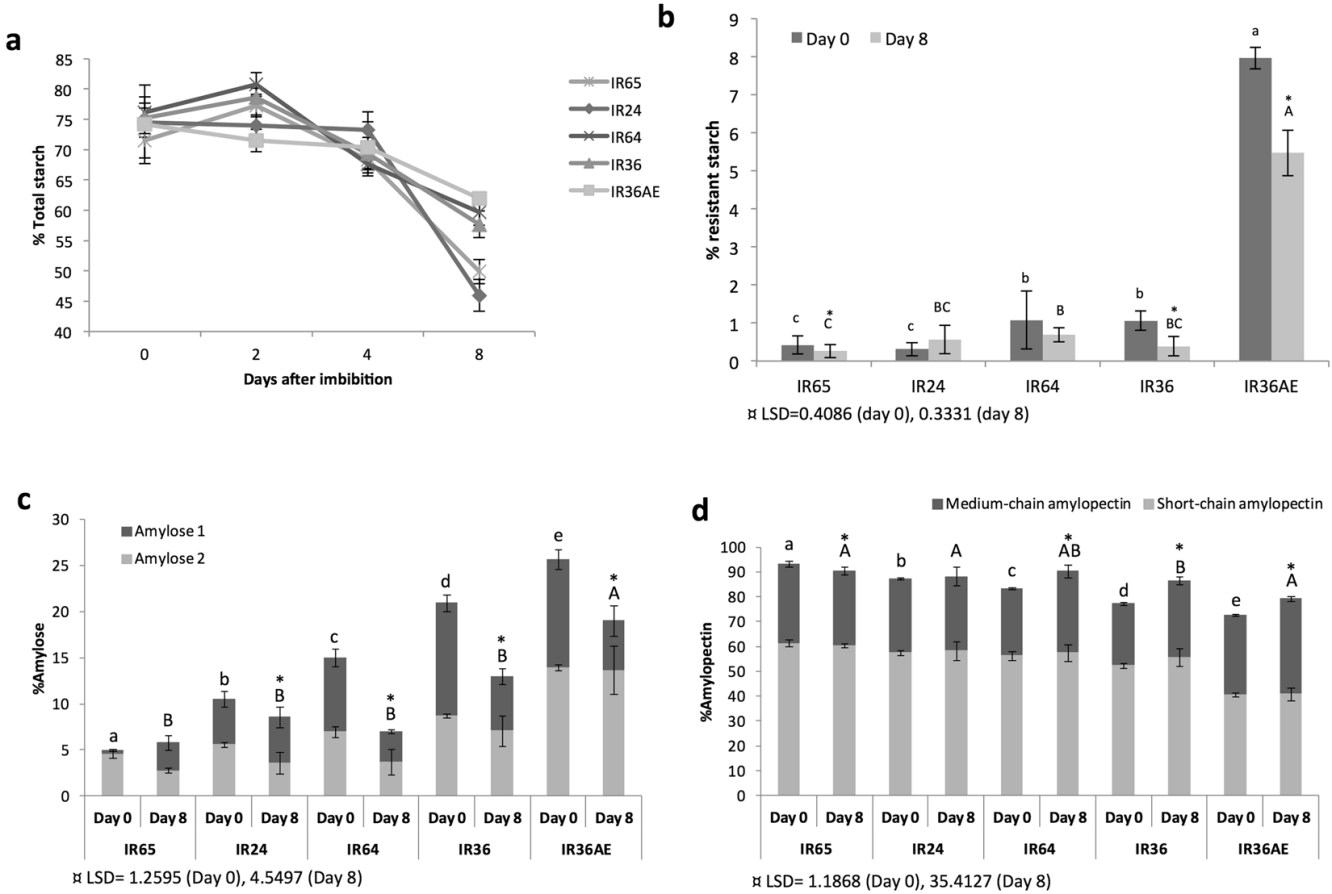
Mobilization of starch during germination. Amounts of (**a**) total starch and (**b**) resistant starch in the germinated grain. The changes in the (**c**) amylose and (**d**) amylopectin fractions are shown. Each fraction is calculated based on 100% starch. Values represent the mean ± SD of 3 biological replicates. In each time point, means with the same letter are not significantly different based on pairwise mean comparison of variance through LSD (least significant difference) test. Values of test statistics are indicated by ¤. Means with * are significantly different compared to day 0 *(P* < 0.05).

The five rice varieties confirmed to have varying GI levels by cooked grain amylolysis (*k value*) and starch hydrolysis index (HI) were subjected to germination to quantify the extent of starch mobilization. The starch mobilization patterns of the five GI lines differ as total starch composition varied significantly during germination (Fig. 3a). Before imbibition, the total starch contents of each of the five GI lines fall within 70–76%, which is similar to what was previously reported for rice^30^. During germination, there was a reduction in starch from day 0 to 8 with two distinct patterns. The waxy and low amylose varieties (IR65, IR24) mobilized around 30–40% of the starch, while the intermediate (IR64), high (IR36) and very high (IR36 and IR36ae) amylose types mobilized only 15–25% of the original content in the mature grain. There was a clear distinction between the behavior of the waxy and low amylose samples (IR65 and IR24), which will be referred to as group 1, and the intermediate to very high amylose varieties (IR64, IR36, and IR36ae), which will be referred to as group 2. The lower levels of total starch at 8 days after germination (DAG) in group 1 indicate that more starch was mobilized compared to group 2, despite all samples having statistically comparable proportion of available starch in the beginning (Supplementary Table 2). Moreover, there are higher levels of free glucose and sucrose in group 1 at 4 DAG (Supplementary Fig. S1). These results suggest that it is easier to mobilize starch and convert it into simple sugars in group 1. In a similar manner, it is easier to hydrolyze starch in group 1 based on the digestibility curves from *in vitro* amylolysis (Fig. 2a).

Both the levels of amylose and RS affect the rate of digestion during germination (Fig. 3b and c). The high amylose and elevated RS content of IR36ae contribute to its lower GI value. There are also substantial differences noted in the mobilization of amylose in IR36ae: AM2 remained unchanged between mature grain (0) to 8 DAG in the low GI line and >50% of the AM1 fraction was mobilized (Fig. 3c). At 8 DAG, IR36ae mobilized only 25% of RS. In comparison, the intermediate GI lines (IR64 and IR36) have much lower RS levels. In sharp contrast, the high GI lines (IR65 and IR24) have the least amylose and the least RS in their mature grain and have the fastest starch hydrolysis rate due to their higher amylopectin content (Fig. 3d). This suggests that amylopectin is the most preferred substrate during the mobilization of starch in germinating seedlings at 8 DAG which is followed by AM1, with RS as the least digestible component.

As expected, the mobilization of storage reserves during seed germination results in the production of free sugars such as sucrose and glucose (Supplementary Fig. S1). During the early stages of germination, sucrose was the most abundant. However, during the course of seed germination, sucrose declined and glucose started to accumulate. By 4 DAG, high GI lines (IR65, IR24) possessed higher sucrose and glucose, which suggests that waxy and low amylose varieties can easily hydrolyze starch (composed mostly of amylopectin) into simple sugars. The intermediate and low GI lines had the lowest sucrose and glucose at 4 DAG (Supplementary Fig. S1).

**Figure 4:**
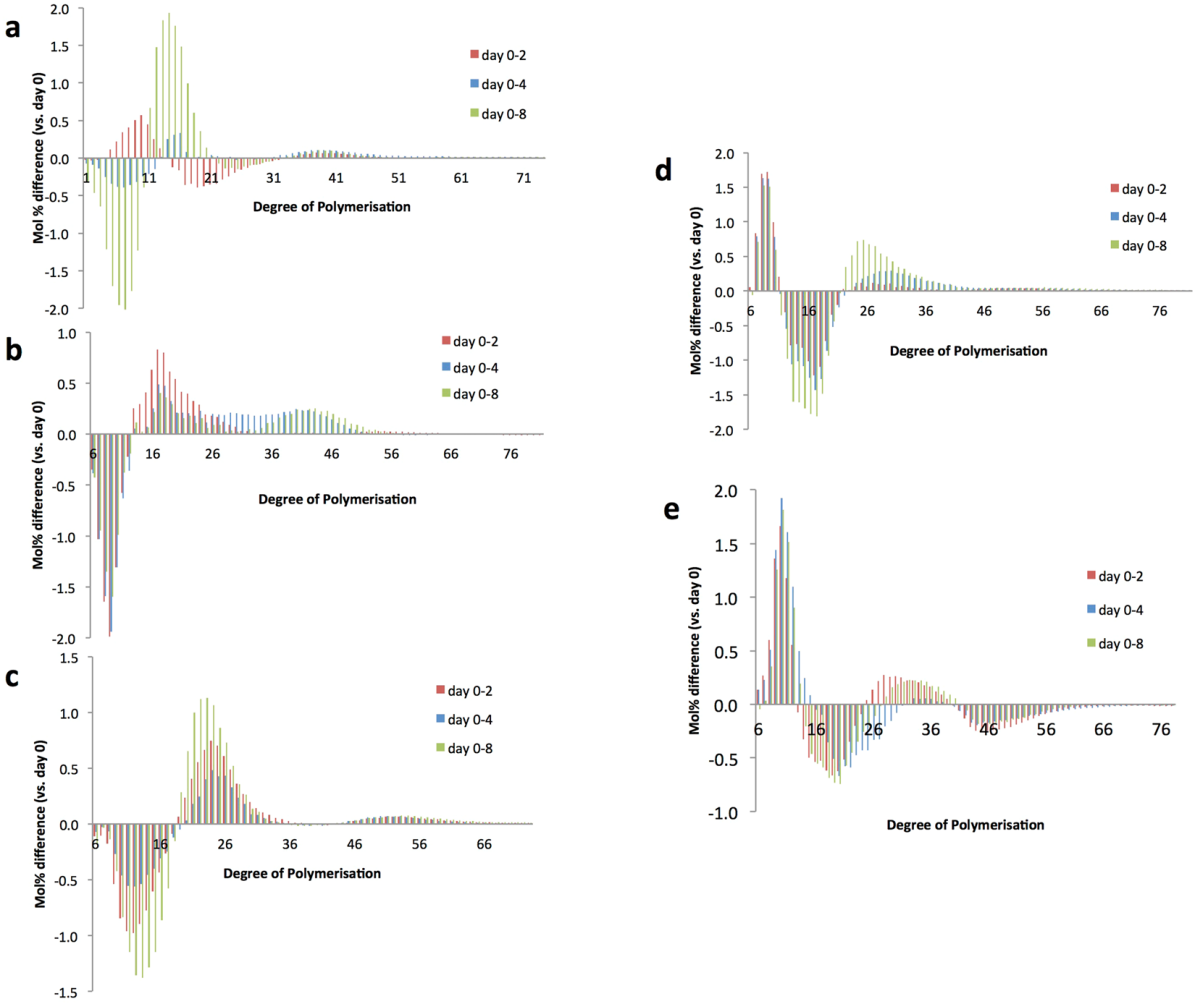
Change in amylopection chain length distribution during germination. Mol% difference of debranched amylopectin chains at 3 different germination time points (days 2, 4 and 8) compared with ungerminated seeds (day 0) for (**a**) IR65, (**b**) IR24, (**c**) IR64, (**d**) IR36, and (**e**) IR36AE. Positive values indicate a decrease while negative values indicate an increase in the amount of chains compared to day 0.

The difference plot of amylopectin chain length distribution (CLD) between mature grain (0 DAG) and 2, 4 and 8 DAG shows two different trends: trend A exhibited by the waxy to intermediate amylose accessions IR65, IR24 and IR64 (Fig. 4a,b and c) and trend B displayed by the high to very high amylose varieties IR36 and IR36ae (Fig. 4d and e). For trend A, major differences include the increase in short chains with DP 6–16 and the decline in DP ≥ 17 onwards during seed germination. For trend B, the opposite is evident: DP 6–12 and DP 22–40 were reduced, while DP 13–22 increased in IR36 at 8 DAG. Likewise, DP 6–12 and DP 22–40 were reduced and DP 27–40 and DP 41–56 increased in IR36ae. It is interesting to note that amylopectin is being used up in different ways as demonstrated by the increase and decrease of certain chain lengths. As a collective measure, profiling for starch composition, free sugars and amylopectin CLD provides novel insights into the differential starch mobilization patterns of selected rice genotypes during seed germination (Table 1).

**Table 1:** Summary of differentiating parameters for the three GI categories. Positive results are marked with (●) and negative results with (○).

Parameters	Low GI	Intermediate GI	High GI
*In vitro* glycemic index measurement			
<55 Predictive glycemic index value	●	○	○
<0.025 digestion rate constant (*k value*)	●	○	○
Mature grain (Day 0)			
≥2% Resistant starch	●	○	○
≥10% Amylose 1 (DP > 1000)	●	○	○
≥10% Amylose 2 (DP 121–1000)	●	○	○
≥50% Short-chain amylopectin (DP 6–36)	○	●	●
Changes during starch mobilization (8 days after imbibition)			
≥30% Decrease in total starch	○	○	●
Decrease in resistant starch	●	●	○
Decrease in Amylose 1 (DP > 1000)	●	●	○
Decrease in Amylose 2 (DP 121–1000)	○	●	●
Increase in medium-chain amylopectin (DP 37–120)	●	●	○
%mol difference: increase in DP 6–16	○	NA	●
%mol difference: increase in DP 13–22	●	NA	○
%mol difference: increase in DP ≥ 41	●	○	○
<15% increase in free glucose (day 0 vs day 4)	●	○	○

### Transcriptome and metabolite analyses of mature grains reveal the importance of storage macronutrient interaction with starch in conferring variations in food matrix that influence rice grain digestibility

**Figure 5:**
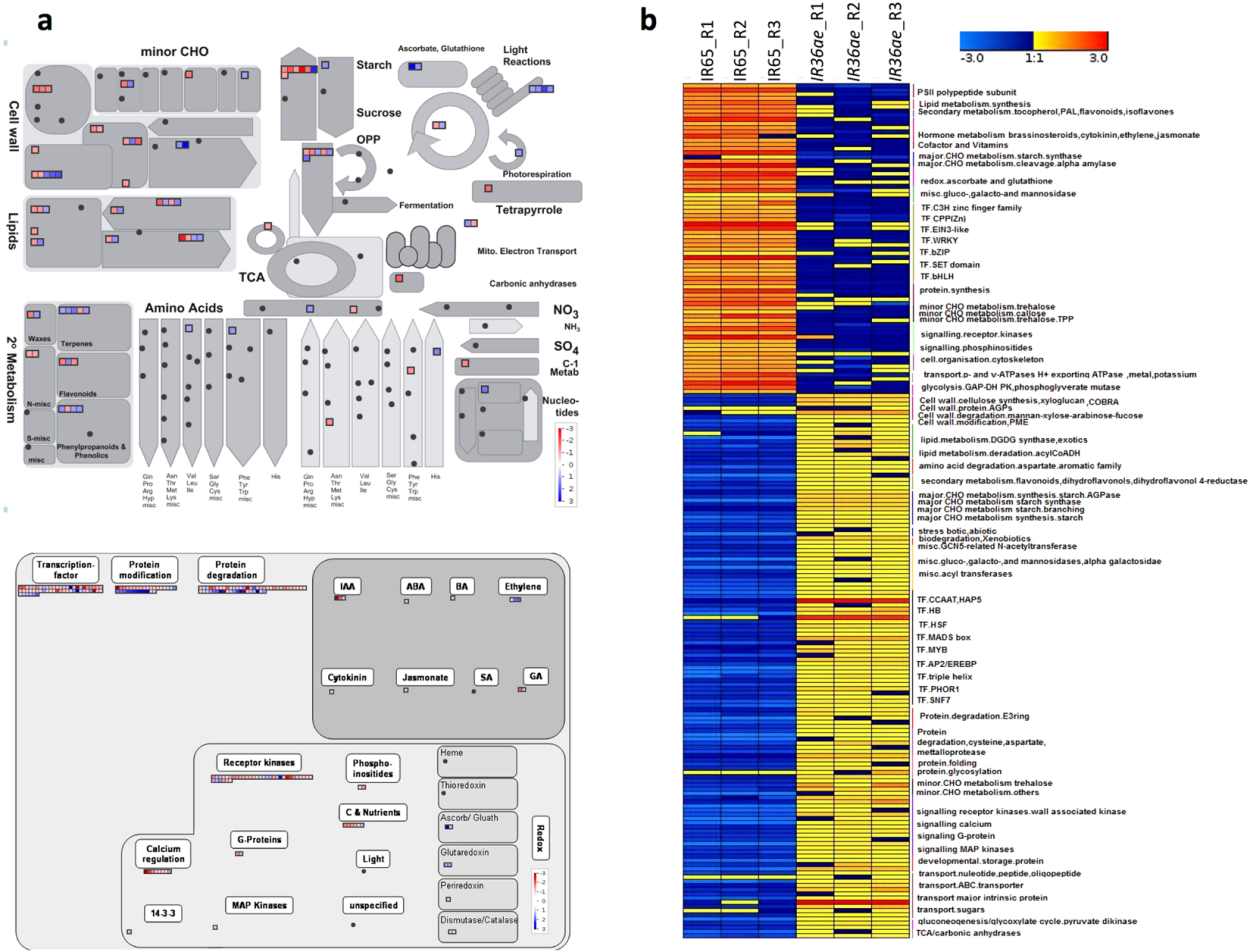
Transcriptomics analysis of low vs. high GI lines. (**a**) MapMan overview of differences in metabolites between low and high glycemic index varieties and (**b**) heatmap showing differentially expressed genes.

Genome-wide transcriptome analysis of GI contrasting lines was conducted to unravel the shifts in metabolic pathways based on the presence of stored mRNA in the mature seeds. In total, 1511 genes were found to be differentially expressed between high GI (IR65) and low GI (IR36ae) lines. Among these, 821 genes were preferentially upregulated in the low GI line and 690 genes were downregulated (Supplementary Table 3). The fold change of differentially expressed genes (DEGs) calculated between low GI and high GI lines is plotted in the MapMan metabolic and regulatory pathway chart (Fig. 5a). The normalized expression values are shown as heat map along with their gene annotation (Fig. 5b). In the low GI line, a higher transcript abundance was found in reserve accumulation pathways for storage starch [ADP-glucose pyrophosphorylase (AGPase) large and small subunits, granule bound starch synthase 1 (*GBSS 1*), starch synthase 3 (*SS 3*), plant glycogenin-like starch initiation protein (*PGSIP*), sugar transporters], pyruvate orthophosphate dikinase (*PPDK*) and storage protein (Glutelin type-A 3, Glutelin type-B 1, Glutelin type-B 2, Glutelin type-B 4, and Glutelin type-B 5). In addition, specific differences were noticed in cell wall metabolism, lipid metabolism and isoflavonoid pathways between low GI and high GI lines. Within the cell wall metabolism pathways, the preferential upregulation of genes related to the synthesis of cellulose, xyloglucan, homogalacturonan, arabinogalactan and expansins was observed in the low GI line. In lipid storage pathways, a higher transcript abundance was detected in phospholipid synthesis (phosphoethanolamine N-methyltransferase) and glycolipid synthesis (digalactosyldiacylglycerol synthase 1), which suggests the importance of starch-lipid complexation in the low GI line as a consequence of elevated amylose content. Among the regulators, preferential upregulation in the low GI line was seen in various MYB transcription factors (TFs) including *GAMYB*, *bHLH*, *Homeobox Hox29*, *MADSbox27*, *WRKY75*, cell wall-associated kinases, various receptor kinases and sugar and nutrient signaling.

**Figure 6:**
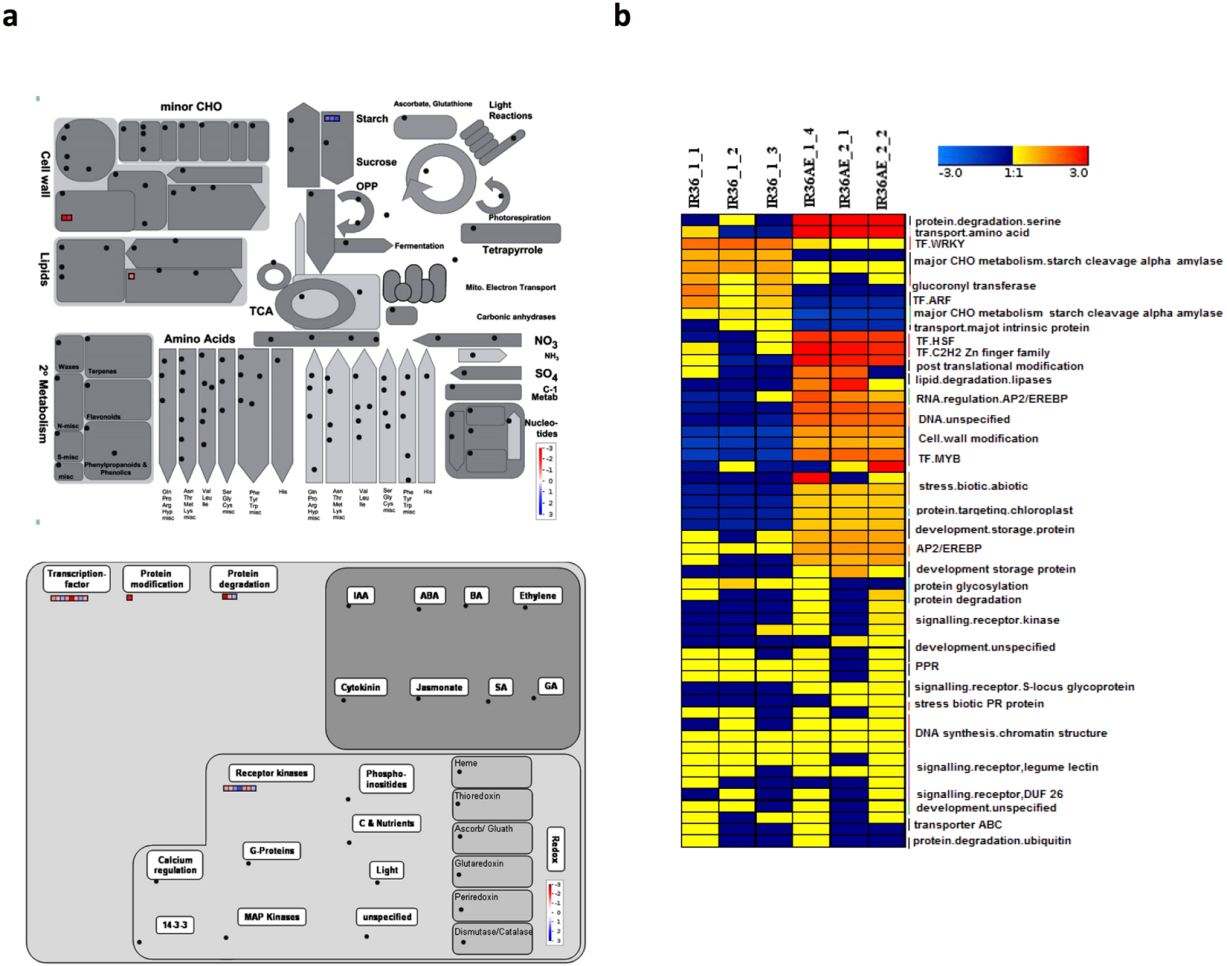
Transcriptomics analysis of intermediate vs. low GI lines. (**a**) MapMan overview of differences in metabolites between low and intermediate glycemic index varieties having the same genetic background and (**b**) heatmap showing differentially expressed genes.

Transcriptome responses were further analyzed in the mature paddy of IR36ae (low GI line) compared with its wild type parent IR36 (intermediate GI line) in the near isogenic background^31, 32^ to rule out varietal background effects. A total of 154 DEGs were identified (Fig. 6). Among these, 107 genes were preferentially downregulated and 47 were upregulated (Supplementary Table 4). IR36ae has a defective starch branching enzyme IIb (*SBEIIb*) which leads to the accumulation of less branched amylopectin chains due to a stop codon mutation of its structural gene^4^. *SBEIIb* probe is missing in the microarray, hence the downregulation of the *SBEIIb* gene expression in this *amylose extender* mutant could not be confirmed in this study. However, the knockout mutation of this gene has been functionally confirmed by previous study using targeted gene sequencing and proteomics approaches^4^. In addition, lower transcript levels of α-amylase gene family members were observed in the mature paddy of the low GI line. The preferential upregulation of key starch biosynthetic pathway to elevate amylose and long chain amylopectin, in conjunction with the suppression of starch mobilizing transcripts in mature seeds, ensures slower digestibility (Fig. 6). Also, a higher abundance of transcripts was observed for cell wall metabolism (*expansin A-19* and *A-20*, *UDP glucosyl and glucoronyl transferases*) and *phospholipase A1-II* from the lipid degradation pathway. This can act as a stored mRNA pool for initiating translating mechanisms to mobilize the cell wall matrix and starch-lipid complex during seed germination in the low GI line. In addition, a higher abundance of transcripts was seen for various storage proteins (*Glutelin type-A 1* and *Glutelin type-B 2*), which corroborated with the higher percentage of proteins in the low GI line in comparison to its wild type (Supplementary Table 1). Among the key regulators, there was enrichment for various receptor kinases and TF family members such as *MYB*, *AP2/EREBP*, *WRKY*, *ARF*, *HSF*, and *C*_*2*_ *H*_*2*_. Among the DEGs, 101 genes were uncharacterized unknown genes and their functional relevance in the context of the low GI phenotype is yet to be determined.

**Figure 7:**
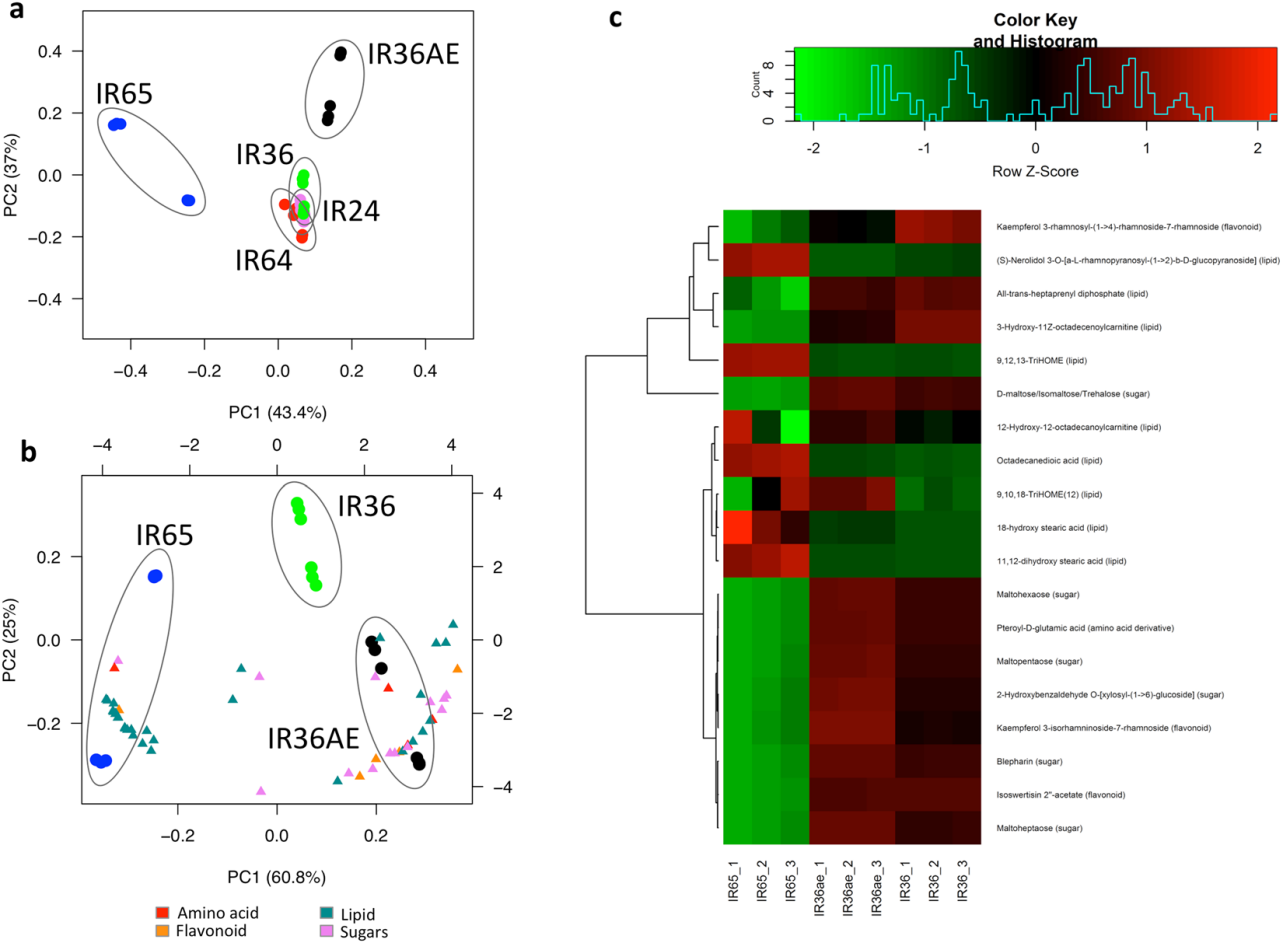
Metabolomics analysis of low, intermediate and high GI lines. PCA plot of (**a**) five contrasting lines with the (**b**) different classes of metabolites that are present in the low, intermediate, and high GI lines. (**c**) Heatmap showing the levels of the metabolites that distinguish the different glycemic index classifications.

Lastly, ultra performance liquid chromatography-mass spectrometry (UPLC-MS) was conducted to determine the metabolites present in the low, intermediate and high GI lines. A total of 1839 peaks were detected which corresponds to 17,000 possible metabolites. These detected metabolites clearly distinguish the low, intermediate and high GI lines based on principal component analysis (Fig. 7a and b). Among these metabolites, flavonoids such as isoswertisin 2″-acetate^33, 34^ and derivatives of kaempferol^34, 35^, which can act as potential α-amylase inhibitors, were found to be present at higher levels in the low GI line (Fig. 7c). Similarly, sugars such as maltose, maltopentaose, maltoheptaose and maltohexaose are more abundant in IR36ae. On the other hand, a number of lipids such as isomers of hydroxy stearic acid, octadecanedioic acid, and 9,12,13-TriHOME are more abundant in the high GI line. The presence of these metabolites corroborates the findings derived from the transcriptome analysis.

## Discussion

Starch-rich cereals including rice commonly give an intermediate to high glycemic response ranging from 64–93^25, 36^. Estimating glycemic response through clinical methods is not routinely applied in rice breeding programs to screen for germplasms with low GI as these methods are expensive, low throughput and require clinical facilities. To circumvent this constraint, the predictive GI based on a validated *in vitro* method has been applied to rice and has demonstrated good correlations with clinical GI scores^5, 25^.

In general, rice grain is primarily composed of starch with a higher proportion of amylopectin contributing to rapid digestibility^7^. Understanding starch structure and proportion, together with determining the amount of RS, is important to modify rice into rapidly digestible, slowly digestible and/or non-digestible (RS) food^4, 6, 7, 37^. Studying a set of diverse 27 breeding lines with different GI values revealed that high levels of AM1 (true long amylose chains) and AM2 (longer amylopectin which behaves like amylose^5^) fractions are associated with lower GIs. Conversely, high amounts of SCAP elevate the GI value (Fig. 1). These observations corroborate with our previous findings^5, 7, 25^. Alternatively, determining the kinetics of starch granule amylolysis provides another way of measuring GI *in vitro* ^10^. Our results revealed that the digestibility curve generated by cooked grain amylolysis agrees with the GI data (Fig. 2). The digestibility kinetics (*k*) *value* also provides a reliable alternative measure of GI since it directly reflects the rate and extent of starch digestion. Based on this result, the waxy variety (IR65) is the rapidly digestible line, while the high amylose variety (IR36ae) is the least digestible.

Aside from starch structure, the proportion of RS also has a significant contribution to digestibility. If the amylose and RS content is high, GI values decrease substantially since amylose is slowly digestible and RS is resistant to digestive enzyme hydrolysis^38, 39^. RS escapes digestion in the small intestine and proceeds to the large intestine where it is fermented by colonic bacteria^40, 41^. IR65 and IR24 have the lowest RS, followed by IR64 and IR36, with IR36ae having the highest RS at 8% (Fig. 3b). This trend in RS contributes to the digestibility curves presented in Fig. 2a. Aside from the effect of amylose, the large quantity of RS in IR36ae made it the least digestible among the samples. Taken together, AM1, AM2 and RS fractions were least mobilized at 8 DAG.

Starch is the major energy reserve used by the seed during germination until it becomes autotrophic. The decrease in the amount of total starch indicates that starch is being utilized (Fig. 3a). The amylose and RS fractions were hydrolyzed with slower rate as suggested by the moderate decline in their proportion. Earlier studies have reported that the preferential hydrolysis of amylopectin occurs during the early stage of germination which results in the release of free sugars^42^. The difference plot of capillary electrophoresis (CE) (Fig. 5) shows how the quantities of amylopectin chains changed throughout the germination period. Since α-amylase cleaves α (1 → 4) bonds at random points within the chain, DP ≤ 80 could have been produced from the cleavage of amylose. This means that at days 2, 4 and 8, the measured CLD is not exclusively from the apparent amylopectin fraction but is rather generated through the amylose mobilization process. Two trends (A and B) can be observed from the behavior of amylopectin during germination. For trend A, the increase in DP 6–12 is potentially due to the activity of a combination of enzymes: α-amylase, β-amylase and debranching enzymes (DBEs). Amylopectin is first degraded by α-amylase to produce limit dextrins. These are hydrolyzed by β-amylase to produce maltose. The remaining branched structures are acted upon by DBEs which cut α (1 → 6) branch points. All these results in the subsequent enrichment of short chains from the CLD data obtained by CE. This is also the reason for the decrease in DP ≥ 17 as these were trimmed and converted into shorter forms. For trend B, the opposite is true, with significant reduction in DP 6–12. The β-amylase of IR36 and IR36ae is possibly more active, leading to the formation of more maltose from the short chains, thereby substantially reducing the proportion of DP 6–12. Chains of DP ≥ 23 decreased due to its breakdown and conversion into DP 13–22, which conversely increased in amount. For IR36ae, DP ≥ 41 also increased but this corresponds only to <0.5%, which is potentially generated through amylose hydrolysis by α-amylase during imbibition.

Using genome-wide transcriptome profiling approach, key starch biosynthetic genes such as *AGPase* (large and small subunits), *GBSS 1* (involved in amylose elevation), *SS3*, *PGSIP*, sugar transporters and *PPDK* were found to be more abundant in the low GI (IR36ae*)* than in the high GI (IR65) lines. AGPase catalyzes the first committed step in starch biosynthesis by the ATP-dependent conversion of glucose-1-phosphate to ADP-glucose^43^. On the other hand, alternative splicing in the first exon/intron boundary of *GBSS 1* determines the conversion of low or no amylose into high amylose rice due to allelic variations in its structural gene^44, 45^. The upregulation of these three key genes and other genes for sugar transporters drive the sugar flux towards the synthesis of higher proportions of AM1 and AM2 chains^7^, thus explaining the very high amylose phenotype of IR36ae. In addition, the α-amylase transcripts are suppressed in the low GI line, while the high GI line had a higher abundance of these starch hydrolytic transcripts. These are readily synthesized during seed maturation and possibly used during early seed germination to trigger starch mobilization, where a higher proportion of amylopectin is rapidly mobilized in the high GI line.

Non-starch polysaccharides (NSPs), storage lipids and storage proteins in the rice endosperm matrix can also play a role in lowering the GI of rice^7^, the mechanism of which is yet to be verified. Together with the increase in the flux of pure amylose (AM1 fractions), the preferential activation of pathways for phospholipid synthesis and glycolipid synthesis was observed in the low GI line (Fig. 5), which are involved in the formation of amylose-lipid complexes that can further increase the proportion of RS. It has been recently demonstrated that by suppressing the expression of the *SSIIIa* gene in transgenic lines increases the proportion of amylose. In these transgenic lines, carbon partitioning is shifted from amylopectin to amylose through pyruvate orthophosphate dikinase (*PPDK*)^39^. This enzyme is crucial during rice grain filling as it modulates carbon metabolism^46^. The flux shifted to support amylose-lipid complex formation, leading to an elevated RS. This is supported by the previous observation that IR36ae has elevated levels of amylose, leading to a proportional increase in starch-complexed phospholipids^47^. In contrast, free lipids were detected in higher levels in IR65 (Fig. 7c). Their abundance in this high GI line could be a possible consequence of the absence of amylose-lipid complexes considering that amylose is absent in this waxy rice variety.

Transcriptome analyses also revealed that the genes for cellulose, xyloglucan, homogalacturonan, arabinogalactan and expansin synthesis were preferentially upregulated in the low GI line IR36ae (Fig. 5). This suggests that the low GI line also has a preferential carbon sink for NSPs. An increase in NSP cell wall components can confer a reduction in digestibility^7, 48^. This is true for legumes where intact cell walls are required to reduce starch digestibility^49^. Moreover, the degradation of cell wall is a prerequisite in the mobilization of starch during barley germination^50^. It appears that a variation in NSP can also alter germination and digestibility rates in rice. In addition, it appears that isoflavonoids and glutelin storage proteins can also play a role in modulating rice grain digestibility by inhibiting α-amylase activity. *In vitro* studies have shown that flavonoids can inhibit the action of human α-amylase^51, 52^. This inhibitory property is attributed to the conjugated π-system that forms between Trp^59^ of α-amylase and the AC- or B-ring of the flavonoid scaffold. The presence of –OH groups that bind to the side chains of Asp^197^ and Glu^233^ are also involved in inhibition^51^. In addition, α-amylase inhibitors were found to be preferentially expressed in the mature seed, previously demonstrated to interfere with α-amylase activity by adsorption and complexation during amylolysis of cooked rice grains^53^. Based on these mechanistic information, the flavonoids detected in abundance in IR36ae (Fig. 7c) are potential α-amylase inhibitors. In conjunction, lowered abundances of α-amylase transcripts in the mature seed were observed in IR36ae.

It appears that the process of starch hydrolysis that occurs during digestion is analogous to the mobilization of stored grain reserves during germination in that both processes release energy. Starch digestion releases glucose which is an important nutrient for animals whereas germination converts storage starch to simple sugars to drive seedling growth. The mobilization pattern of the individual components of the grain during germination reflects the glycemic potential of a genotype (Table 1). In particular, the total starch content, amylose, and RS levels at 8 DAG, as well as the amounts of free glucose and sucrose at 4 DAG reveal the mechanism of starch mobilization during germination. In addition, changes in CLD of amylopectin exhibited a distinction between the low and high GI samples. These results are comparable to the digestibility of cooked rice as determined by *in vitro* starch hydrolysis kinetics and predicted GI measurements. In this context, biochemical changes during germination can serve as an alternative proxy measure to estimate GI.

Moreover, the digestion curves suggest that the variety IR65 with pure amylopectin (i.e. no AM1) leads to rapid starch digestibility, where key genes involved in starch biosynthesis such as *AGPase*, *GBSS 1* and *SS3* were lowly expressed, while alpha amylase transcripts were abundantly enriched. As compared to IR36 wild type seeds with intermediate GI, the significantly lower GI of IR36ae highlights the importance of shifting the carbon flux towards amylose and high RS. Aside from *SBEIIb* mutation previously reported in IR36ae^4^, metabolomics and transcriptomics analyses revealed that storage proteins, amylose-complexed lipids, flavonoids and NSPs were preferentially upregulated in the near isogenic mutant compared to its parental line IR36 (Supplementary Table 3 and 4).

In summary, we have provided a corroborative set of evidences in this study to support that cooked rice grain digestibility is primarily due to the proportion and structure of storage starch in the grain. In addition, the importance of the interaction of storage starch with proteins, lipids, cell wall polysaccharides and isoflavonoid α-amylase inhibitors are critical underlying factors to distinguish between RS and SDS^54^ in milled rice grains. Additional structural and functional genomics studies are required to validate genes that significantly influence SDS and RS formation. The associated candidate genes will be future targets to manipulate the digestibility and enhance dietary fiber and RS levels in rice grains.

## Methods

### Cooked rice amylolysis

Milled rice flour samples were ground and sieved to obtain particles in the range of 400–600 microns. In 50 mL glass test tubes, 500 mg flour was cooked by soaking in water for 30 minutes and subsequent boiling over 100 °C water bath for another 30 minutes. The amount of water added was dependent on the apparent amylose content of the sample as described in Butardo *et al*.^5^. After cooking, the tubes were transferred to 37 °C water bath over a stirring plate and *in vitro* starch digestibility was carried out immediately for the freshly-cooked samples to avoid retrogradation. Rice clumps were first broken down by the addition of 6 mL water (37 °C) and subsequent stirring at 350 rpm. The following were added consecutively, with their corresponding incubation time: 5 mL pepsin (1 mg/mL in 0.01 M HCl) for 30 minutes, 5 mL 0.02 M NaOH for 1 minute, and 20 mL 0.2 M NaOAc buffer (pH 6.0, 0.49 mM MgCl_2_, 4 mM CaCl_2_) for 10 minutes. A 200 µL aliquot (duplicate) was transferred to microfuge tube (labeled as time 0). Afterwards, 5 mL pancreatin/amyloglucosidase (AMG) solution (2 mg/ml pancreatin and 28 U/ml AMG in 0.2 M NaOAc buffer pH 6.0 with 0.49 mM MgCl_2_ and 4 mM CaCl_2_) was added to the test tube. At the following sampling points, 200 µL aliquots (duplicate) were obtained: 5, 10, 20, 30, 45, 60, 90, 120, and 180 minutes after the addition of the pancreatin/AMG solution. Aliquots were rapidly immersed in ice bath to stop enzyme digestion, centrifuged at 13,000 rpm (4 °C) for 10 minutes to collect the supernatant, and stored at −80 °C before measuring the glucose.

Glucose was measured based on Megazyme’s D-glucose assay kit (K-GLUC). In 2 mL microfuge tubes, 50 µL solution (with proper dilution) was transferred and 5 µL AMG solution (300 U/mL AMG in 0.2 M NaOAc buffer pH 6.0 with 0.49 mM MgCl_2_ and 4 mM CaCl_2_) was added. The mixture was incubated for 20 minutes in 50 °C. GOPOD reagent (1.5 mL) was added and the solution was incubated at 50 °C for 20 minutes. Absorbance (Beckman Coulter DU 800 spectrophotometer) was read at 510 nm using water as blank. A calibration curve was generated using standard glucose solution treated similarly as the sample.

### Rice seed germination

Rice grains were dehulled and cleaned by washing with 70% ethanol for 30 seconds and sterile water multiple times. Afterwards, the seeds were placed on sterile 100 × 20 mm petri dishes lined with sterile Whatman filter paper (90 mm circles) moistened with gibberellic acid (GA3) as described by the International Rules for Seed Testing (ISTA). Plates were incubated at 35 °C, with 12/12 light-dark cycle and ≥70% relative humidity for 2, 4, and 8 days. At the end of germination, the shoot, roots, and scutellum were excised and the remaining grains were freeze-dried, ground, and stored at −80 °C prior to analysis.

### *In vitro* determination of GI of cooked milled rice grains

The predicted GI values of the 27 breeding lines were determined using the *in vitro* GI measurement described in Fitzgerald *et al*.^25^ and Butardo *et al*.^5, 25^. Briefly, rice grains were cooked for 16 minutes and allowed to cool for 5 minutes at room temperature. Cooked grains were mixed with artificial saliva (250 U/mL α-amylase) and incubated in pepsin (1 mg/mL) for 30 minutes with shaking at 37 °C. The pH was adjusted to 6 followed by the addition of pancreatin (2 mg/mL) and AMG solution (28 U/mL). The samples were digested for 5 hours and aliquots were obtained at different time intervals. The GI was calculated using the concentration of glucose determined with an automated electrochemical method.

### Total starch determination

Total starch was measured using Megazyme total starch assay kit (K-TSTA), with sample and reagent amounts downscaled ten-fold as originally optimized by Butardo *et al*.^5^. Briefly, 10 mg ground brown rice was incubated in 0.5 ml ethanol (80%v/v) at 80 °C for 5 minutes. After incubation, the same volume of ethanol was added and the samples were centrifuged. The recovered pellet was resuspended in 1 mL ethanol (80%v/v) and centrifuged as before. The supernatant was removed and 0.2 mL 2 M KOH was added. Samples were stirred for 20 minutes while submerged in an ice bath. While stirring, 0.8 mL 1.2 M sodium acetate buffer (pH 3.8) was added, followed by 0.01 mL of α-amylase (3,000 U/ml) and 0.01 mL of AMG solution (3,300 U/ml). Samples were incubated at 50 °C for 30 minutes with intermittent mixing and were centrifuged. A 0.1 mL aliquot was obtained and 3.0 mL GOPOD reagent was added. Samples were incubated at 50 °C for 20 minutes. The absorbance (Beckman Coulter DU 800 spectrophotometer) was determined at 510 nm against a blank solution consisting of water and GOPOD. Total starch was calculated based on the absorbance of 1 mg/ml D-glucose standard.

### Characterization of debranched starch by size exclusion chromatography

Prior to analysis, Waters Alliance 2695 fitted with Ultrahydrogel 250 (Waters) was calibrated for molecular weight using pullulan standards (P-82 Shodex, Showa Denko, K. K. Kawasaki, Japan). Universal calibration was performed using Mark–Houwink–Sakaruda equation (*K* = 0.00126mLg^–1^ and a = 0.733 for pullulan, and *K* = 0.0544mLg^–1^ and a = 0.486 for linear starch) as described in Castro *et al*.^55^. Waters 2414 Refractive Index was used as detector and 0.05 M NH_4_OAc pH 4.75 with 0.02% sodium azide as mobile phase.

Brown rice flour (55 mg) was gelatinized by the addition of 400 μL of 95% ethanol and 1 mL of 0.25 M NaOH. The mixture was heated at 150 °C for 12 minutes while stirring, with the addition of 0.8 mL hot water in 800 μL aliquots (three times). The total weight of the solution was adjusted to 4 g by the further addition of hot water. To debranch the gelatinized solution, 794 µL aliquot was mixed with 206 µL of sodium acetate buffer (10 mL 0.2 M NaOAc pH 4.0 with 360 µL glacial acetic acid). The solution was incubated with 8 μL of isoamylase (Megazyme, P113541) at 50 °C water bath for 2 hours with intermittent mixing. Afterwards, the tubes were placed in boiling water bath for 5 minutes and centrifuged at 12,500 rpm for 10 minutes. The supernatant was transferred to a tube containing approximately 320 mg ion exchange resin (Bio-Rad AG 501-X8 (D)) and incubated for 30 minutes at 50 °C with mixing every 10 minutes. The same conditions for calibration were used for the samples, with an injection volume of 40 µL and stop time of 35 minutes.

### Chain length distribution by capillary electrophoresis

An aliquot of the debranched sample (100 µL) from the SEC was dried (Speedvac Concentrator Savant, Sunnyvale, CA, USA) and 3.5 µL 0.2 M 8-amino-1, 3, 6-pyrenetrisulfonic acid (APTS) (Sigma, St Louis, MO, USA) in 15% acetic acid and 3.5 mL 1 M sodium cyanoborohydride was added. The mixture was incubated for 16 hours at 50 °C to label the samples. Afterwards, 40 μL 6 M urea and 40 µL water were added. The reaction mixture was filtered and transferred into vials. The labelled amylopectin chains were separated by CE^56^ (P/ACE MDQ Beckman Coulter, Fullerton, CA, USA) using N-CHO capillary (PA800 Beckman Coulter) and data were recorded and analysed using 32 Karat 7.0 software (Fullerton, CA, USA). The mol% was calculated by getting the percentage area of each peak with respect to total area.

### Free sugars determination by HPLC

Free sugars were extracted based on the method described in Megazyme total starch assay kit (K-TSTA). Briefly, brown rice flour (100 mg) was extracted with 1 mL hot 80% ethanol three times. Each time, the sample was centrifuged at 12,500 rpm for 10 minutes to obtain the supernatant. The extracts were pooled together, dried (Speedvac Concentrator Savant, Sunnyvale, CA, USA), and stored at −80 °C prior to analysis.

Dried extracts were reconstituted with 0.5 ml water and boiled in a water bath for 5 minutes. Samples were transferred to a spin column (Biorad, Freeze and squeeze, 7326166) and centrifuged at 12,500 rpm for 10 minutes. The resulting supernatant was used for HPLC analysis with Sugar-Pak I column (Waters, WAT085188) installed in the Waters Alliance 2695 system equipped with Waters 2414 Refractive Index detector. Samples were eluted using 1 × 10^−4^ M Ca EDTA (65 °C) at a flow rate of 0.50 ml/min with an injection volume of 50 µL and column temperature of 60 °C. Sucrose (Sigma, S9378-10MG), glucose (Sigma Ultra, S7903-250G), and fructose (Sigma Ultra, F2543-100G) were used as standards.

### Resistant and non-resistant starch measurement

Both resistant and non-resistant starch were analysed using Megazyme resistant starch kit (K-RSTAR), with sample and reagent amounts downscaled ten-fold as originally optimized by Butardo *et al*.^5^. Brown rice flour (10 mg) was digested with 0.4 ml enzyme (10 mg/mL pancreatic α-amylase and 3.0 U/mL AMG) for 16 hours at 37 °C with shaking (100 strokes/minute). After overnight incubation for exactly 16 hours, 0.4 mL 99% ethanol was added to stop the reaction. Samples were centrifuged for 20 minutes at 12,000 rpm. The supernatant was collected and the residue was washed serially with 0.2 mL and 0.6 mL 50% ethanol (twice). The washings were pooled and combined with the supernatant collected after digestion.

To the remaining residue, 0.2 mL 2 M KOH was added and incubated for 20 minutes in an ice bath with stirring. The solution was continuously stirred with the addition of 0.8 mL 1.2 M sodium acetate buffer. Then 10 µL AMG (3,300 U/mL) was immediately added and the solution was vortexed. Tubes were incubated at 50 °C water bath for 30 minutes, with intermittent mixing by vortex mixer every 15 minutes. Samples were diluted appropriately and 0.1 mL aliquot was obtained and allowed to react with 3 mL GOPOD reagent for 20 minutes in 50 °C water bath. The absorbance (Beckman Coulter DU 800 spectrophotometer) was determined at 510 nm against a blank solution consisting of water and GOPOD.

The pooled washings and supernatant were diluted to 10 mL with pH 4.5 100 mM sodium acetate buffer. A 0.1 mL aliquot was reacted with 10 µL AMG (300 U/mL) and incubated for 20 minutes at 50 °C. A second incubation with 3 mL GOPOD reagent followed at the same temperature and time interval. The absorbance was determined in Beckman Coulter DU 800 spectrophotometer at 510 nm against a solution consisting of buffer and GOPOD.

### Transcriptome analysis

The mature paddy grains were used to isolate RNA using the Trizol method, cleaned with RNeasy spin columns (QIAGEN), and RNA quality was assessed using Agilent Bioanalyzer 2100. The purified RNA was subjected to probe synthesis and labeling as described in Butardo *et al*.^7^. The Cyanin 3 labelled probes from IR65 (high), IR36 (intermediate), and IR36ae (low) GI lines were hybridized to Agilent single channel 57k microarray chip and data were analysed consisting of 9 arrays with three replicates each. The data were normalized using GeneSpringGX (Agilent, CA, USA) following quantile normalization algorithm. The DEGs between IR65 and IR36ae and between IR36 and IR36ae were identified following the empirical Bayes method to shrink the probe-wise sample variances towards a common value and to augment the degrees of freedom for the individual variances (Smyth, 2004).The P-values adjustment method used was Bonferroni. The top-ranked genes were selected having a P-value below 0.05 and fold change above +/− 1. The DEGs were further subjected to pathway analysis using Mapman software. The details of metabolic regulation overview and cellular response have been constructed based on the fold change value above +/− 1 among the contrasting lines. The heatmap for DEGs between IR65 and IR36ae and between IR36 and IR36ae was constructed by Genesis software. For specific functional processes, the heatmap has been shown for IR65, IR36ae, and IR36 lines.

### Metabolomics analysis by UPLC-MS

Metabolites were extracted from 100 mg rice flour using 1 mL methanol. The suspension was mixed using vortex mixer at room temperature and centrifuged to separate the supernatant. The extract was analyzed using Waters Acquity UPLC system equipped with Xevo G2-S QTof as described in Waters application note no. APNT134775221^57^. Briefly, chromatographic separation was achieved using Acquity UPLC HSS T3 (2.1 × 100mm, 1.8 µm). Gradient elution was employed using 0.1% formic acid (solvent A) and acetonitrile (solvent B) at a flowrate of 0.6 mL/min and a stop time of 15 minutes. Column and sample temperatures were kept at 40 °C and 15 °C, respectively.

The ESI source was kept at negative ionization mode with MS^E^ data acquisition strategy. The capillary voltage was set at 2.5 kV, while the cone voltage at 100 V. Source and desolvation temperatures were kept at 120 °C and 500 °C, respectively. Cone gas and desolvation gas flows were 30 L/h and 1000 L/h, respectively. Increasing collision energy was employed at 15–40 eV. Leucine Enkephalin was used as a lock mass being sampled every 60 s (scan for 0.3 s). Chromatogram processing and metabolite identification were conducted using Progenesis QI software.

## Electronic supplementary material

Supplementary figures and tables
