# Supplementary material for: Investigating glycemic potential of rice by unraveling compositional variations in mature grain and starch mobilization patterns during seed germination
Source: Sci Rep. 2017 Jul 19;7:5854. doi: 10.1038/s41598-017-06026-0 (PMC5517638; doi:10.1038/s41598-017-06026-0)
Supplement: Supplementary file 1 — Supplementary figures and tables [file 41598_2017_6026_MOESM1_ESM.pdf]

Investigating glycemic potential of rice by unraveling compositional variations in mature grain and starch mobilization patterns during seed germination

Maria Krishna de Guzman[1,+], Sabiha Parween[1,+], Vito M. Butardo Jr.[1❖], Crisline Mae Alhambra[1], Roslen Anacleto[1], Christiane Seiler[2], Anthony R. Bird[3], Chung-Ping Chow[4¤] and Nese Sreenivasulu[1,*]

[1]Grain Quality and Nutrition Center, Plant Breeding Division, International Rice Research Institute, Los Baños, Laguna, 4030, Philippines

[2]The Leibniz Institute of Plant Genetics and Crop Plant Research (IPK), Gatersleben, Germany

[3]CSIRO Health and Biosecurity, Kintore Ave, Adelaide SA, 5000 Australia

[4]Waters Pacific Pte Ltd, Singapore Science Park II, Singapore 117528

*Corresponding author: n.sreenivasulu@irri.org

[+]These authors contributed equally to this work

[❖]Present affiliation: ARC Industrial Transformation Training Centre for Functional Grains (FGC), Graham Centre for Agricultural Innovation, Charles Sturt University, Wagga Wagga NSW, 2650 Australia

[¤] Present affiliation: Genedata AG, Basel, CH-4053, Switzerland

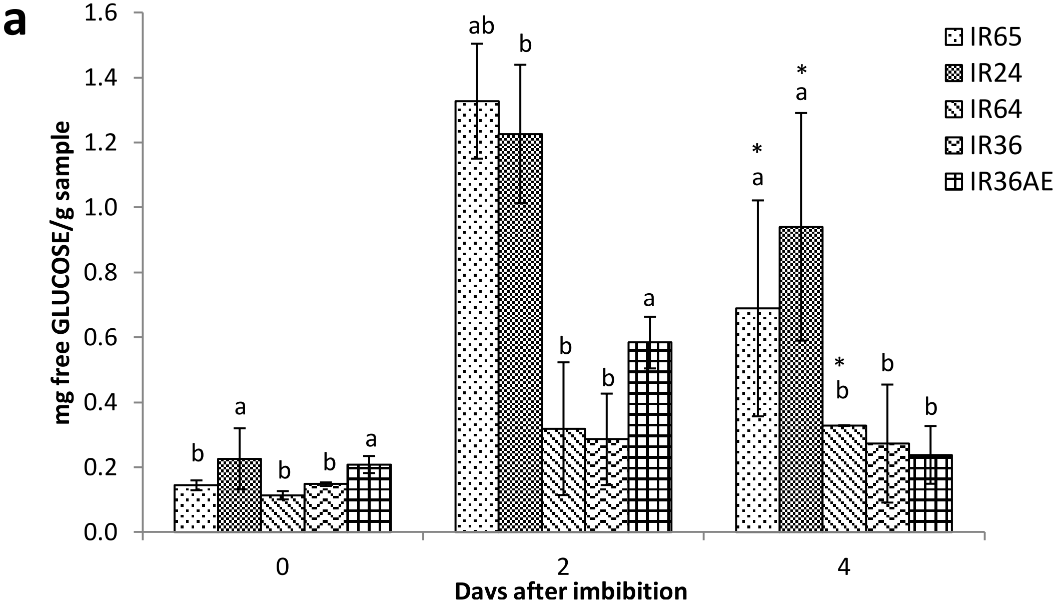

¤ LSD= 0.0540 (Day 0), 0.2214 (Day 2), 0.3053 (Day 4)

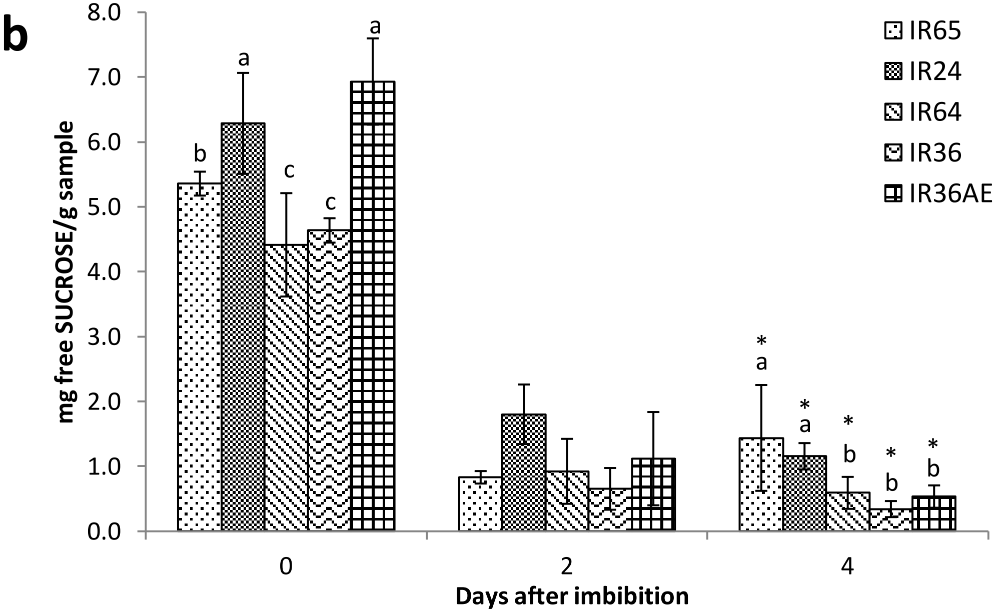

¤ LSD= 0.7036 (Day 0), 0.5205 (Day 4)

Supplementary figure 1. Free sugars analysis. Amounts of (A) free glucose and (B) free sucrose in the germinated grain. Values represent the mean ± SD of 3 biological replicates. In each time point, means with the same letter are not significantly different based on pairwise mean comparison of variance through LSD (least significant difference) test. Values of test statistics are indicated by ¤. Means with * are significantly different compared to day 0 *(P<0.05)*.

Supplementary Table 1. Biochemical properties of five contrasting lines

	IR65	IR24	IR64	IR36	IR36AE
GI classification	High	High	Intermediate	Intermediate	Low
Hydrolysis index	90	79	66	64	48
K value	0.21	0.28	0.17	0.07	0.03
% Resistant starch	0.42	0.31	1.08	1.06	7.96
% Amylose 1	0.46	4.93	8.04	12.24	11.77
% Amylose 2	4.51	5.62	7.00	8.74	13.95
% Medium chain amylopectin	31.79	29.90	27.03	25.01	32.27
% Short chain amylopectin	61.45	57.58	56.40	52.44	40.63
% Protein (Kjeldahl method)	9.04	8.33	8.47	8.33	11.75

Supplementary Table 2. Pairwise comparison of means by least significant difference (LSD) test
*Means with the same letter are not significantly different (P<0.05)

	Grouping			
Variety	% total starch-day 0	%total starch- day 2	%total starch- day 4	%total starch- day 8
IR65		b		c
IR24		c		d
IR64	No significant difference	a	No significant difference	ab
IR36		ab		b
IR36AE		c		a
	% amylose-day 0	%amylose- day 2	%amylose- day 4	%amylose- day 8
IR65	e	e	d	
IR24	d	d	c	
IR64	c	c	bc	No significant difference
IR36	a	b	a	
IR36AE	b	a	b	
	% amylopectin-day 0	%amylopectin- day 2	%amylopectin- day 4	%amylopectin- day 8
IR65	a	a	b	c
IR24	b	b	a	d
IR64	c	c	c	a
IR36	d	d	d	b
IR36AE	d	e	b	a

Supplementary Table 3. List of DEGs between IR36ae vs IR65 with potential candidate genes in bold.

ProbeName	SystematicName	logFC	P.Value	Regulation
A105518	LOC_Os01g65692.1	5.861454904	2.55E-009	Up
A137174	LOC_Os08g28030.1	5.176396881	3.79E-007	Up
A116635	LOC_Os03g61280.1	5.114112384	4.29E-008	Up
A127300	LOC_Os06g12640.1	4.828508253	1.51E-009	Up
A126727	LOC_Os06g06600.1	4.165428561	4.65E-009	Up
A102178	LOC_Os01g26130.1	4.089480058	9.82E-006	Up
A147842	LOC_Os11g32820.1	4.068588223	1.86E-007	Up
A148430	LOC_Os11g39660.1	3.994963498	3.52E-007	Up
A130579	LOC_Os06g50630.1	3.935956781	4.54E-005	Up
A129604	LOC_Os06g40415.1	3.835783354	2.73E-005	Up
A134150	LOC_Os07g41970.1	3.62483516	9.22E-005	Up
A135982	LOC_Os08g13699.1	3.593520472	7.00E-006	Up
A130590	LOC_Os06g50780.1	3.545209266	1.86E-005	Up
A100106	LOC_Os01g02120.1	3.436775765	9.71E-009	Up
A126139	LOC_Os05g51300.1	3.375783564	3.76E-008	Up
A132823	LOC_Os07g27390.1	3.220562826	5.99E-008	Up
A109422	LOC_Os02g36510.1	3.211736017	1.10E-007	Up
A118498	LOC_Os04g20930.1	3.116423185	1.40E-007	Up
A107736	LOC_Os02g16544.1	3.098568347	3.68E-007	Up
A138630	LOC_Os08g43560.1	3.090856577	0.000000033	Up
A130563	LOC_Os06g50450.1	3.051879239	0.000376434	Up
A135936	LOC_Os08g13130.1	3.01515092	1.14E-006	Up
A133603	LOC_Os07g36110.1	2.972305829	3.29E-005	Up
A132261	LOC_Os07g19130.1	2.880337399	0.000000206	Up
A108795	LOC_Os02g29140.1	2.794605117	2.28E-007	Up
A142516	LOC_Os10g07340.1	2.794168421	0.000014589	Up
A146720	LOC_Os11g18044.1	2.773724963	4.08E-006	Up
A109963	LOC_Os02g42690.1	2.74771275	0.000000041	Up
A119272	LOC_Os04g30190.1	2.747513239	0.000000085	Up
A148760	LOC_Os11g43350.1	2.73437061	8.31E-007	Up
A127942	LOC_Os06g20310.1	2.685891474	6.20E-006	Up
A101404	LOC_Os01g16250.1	2.646379942	6.55E-007	Up
A103117	LOC_Os01g39000.1	2.635389428	5.61E-005	Up
A142675	LOC_Os10g09240.1	2.624859318	0.000000091	Up
A109780	LOC_Os02g40280.1	2.620233044	2.04E-005	Up
A147540	LOC_Os11g29500.1	2.6157465	1.24E-007	Up
A116889	LOC_Os03g63999.1	2.589816817	2.68E-005	Up
A146758	LOC_Os11g18550.1	2.544864437	1.13E-007	Up
**A100157**	**LOC_Os01g02700.1**	**2.536394946**	**1.21E-007**	**Up**
A120454	LOC_Os04g43890.1	2.535333993	2.42E-005	Up
A135527	LOC_Os08g08300.1	2.533789591	1.78E-006	Up
**A132459**	**LOC_Os07g22930.2**	**2.527798234**	**3.49E-006**	**Up**
A118429	LOC_Os04g20080.1	2.518846202	4.53E-007	Up
A136753	LOC_Os08g23200.1	2.437868222	5.68E-007	Up
A124303	LOC_Os05g30590.1	2.430216704	7.76E-007	Up

A151896	LOC_Os12g33750.1	**2.425956706**	1.11E-007	Up
A134523	LOC_Os07g46110.1	**2.42170479**	6.69E-006	Up
A126553	LOC_Os06g04840.1	**2.413221378**	2.97E-005	Up
A152211	LOC_Os12g37030.1	**2.407550468**	2.80E-007	Up
A123547	LOC_Os05g20440.1	**2.399121644**	1.17E-005	Up
A142832	LOC_Os10g11210.1	**2.3892481**	2.42E-006	Up
A147952	LOC_Os11g34460.1	**2.389202402**	1.89E-006	Up
A114068	LOC_Os03g30070.1	**2.383759913**	6.85E-007	Up
A151672	LOC_Os12g31140.1	**2.377135525**	1.60E-005	Up
A123401	LOC_Os05g18550.1	**2.365774961**	1.52E-007	Up
A109072	LOC_Os02g32600.1	**2.360033927**	1.78E-005	Up
A143611	LOC_Os10g23240.1	**2.357609382**	1.25E-006	Up
A151710	LOC_Os12g31600.1	**2.349170926**	2.06E-006	Up
A111620	LOC_Os03g01950.1	**2.343747191**	8.74E-006	Up
A135223	LOC_Os08g04880.1	**2.336933253**	4.04E-005	Up
A138880	LOC_Os09g02100.1	**2.321268236**	1.51E-006	Up
A123176	LOC_Os05g15530.1	**2.319067517**	1.90E-007	Up
A147776	LOC_Os11g32140.1	**2.319048563**	4.40E-007	Up
A117681	LOC_Os04g10610.1	**2.291580124**	3.25E-007	Up
A111836	LOC_Os03g04350.1	**2.288748679**	4.45E-007	Up
A138065	LOC_Os08g37650.1	**2.287266608**	0.000320137	Up
A140464	LOC_Os09g21570.1	**2.285493419**	6.46E-007	Up
A101863	LOC_Os01g22430.1	**2.280482369**	0.000005724	Up
A137234	LOC_Os08g28670.1	**2.260654214**	7.34E-006	Up
A102351	LOC_Os01g28690.1	**2.25837709**	1.14E-006	Up
A148343	LOC_Os11g38700.1	**2.240583722**	7.06E-006	Up
A143262	LOC_Os10g19020.1	**2.236693475**	2.01E-005	Up
A118919	LOC_Os04g26060.1	**2.23250735**	2.66E-006	Up
A148321	LOC_Os11g38462.1	**2.231958454**	3.04E-007	Up
A119307	LOC_Os04g30580.1	**2.223958434**	0.000005186	Up
A133688	LOC_Os07g37030.1	**2.213641707**	1.02E-006	Up
A142206	LOC_Os10g03470.1	**2.213496786**	2.78E-007	Up
A117836	LOC_Os04g12400.1	**2.2034143**	4.99E-007	Up
A151383	LOC_Os12g27540.1	**2.201347349**	1.72E-005	Up
A119926	LOC_Os04g38240.1	**2.194205062**	3.79E-006	Up
A142579	LOC_Os10g08060.1	**2.192128566**	7.79E-005	Up
A127438	LOC_Os06g14120.1	**2.189565072**	3.04E-007	Up
A152255	LOC_Os12g37519.2	**2.175122221**	5.23E-007	Up
A124023	LOC_Os05g27020.1	**2.167616873**	1.64E-006	Up
A122194	LOC_Os05g03940.1	**2.159607429**	6.93E-007	Up
A145641	LOC_Os11g05220.1	**2.159182844**	0.000164321	Up
A130466	LOC_Os06g49450.1	**2.156480962**	2.55E-005	Up
A107637	LOC_Os02g15470.1	**2.14891232**	0.000001127	Up
A152229	LOC_Os12g37220.1	**2.148087682**	2.36E-006	Up
A106360	LOC_Os02g01430.1	**2.146404175**	1.42E-006	Up
A151269	LOC_Os12g26080.1	**2.129015379**	2.06E-006	Up
A117632	LOC_Os04g10030.1	**2.123096515**	9.23E-007	Up
A121258	LOC_Os04g52750.1	**2.098004905**	2.71E-007	Up

A122725	LOC_Os05g10240.1	**2.088889839**	0.000001377	Up
A109889	LOC_Os02g41910.1	**2.083373165**	7.84E-006	Up
A110610	LOC_Os02g49300.1	**2.077963874**	7.53E-005	Up
A102238	LOC_Os01g27360.1	**2.074028301**	0.000113077	Up
A148612	LOC_Os11g41740.1	**2.070443783**	0.000002287	Up
A129789	LOC_Os06g42360.1	**2.068069428**	4.19E-005	Up
A114828	LOC_Os03g40600.1	**2.067392631**	1.37E-005	Up
A110802	LOC_Os02g51220.1	**2.060817369**	9.72E-006	Up
A118764	LOC_Os04g24230.1	**2.056981418**	1.61E-006	Up
A110116	LOC_Os02g44155.1	**2.056764737**	0.000410948	Up
A117708	LOC_Os04g10950.1	**2.0520747**	1.25E-006	Up
A140115	LOC_Os09g17280.1	**2.046185171**	0.000012795	Up
A147173	LOC_Os11g25010.1	**2.042437479**	0.00010267	Up
A108944	LOC_Os02g30740.1	**2.036710675**	0.003399062	Up
A151012	LOC_Os12g22890.1	**2.036682169**	2.81E-006	Up
A127656	LOC_Os06g16520.1	**2.033335026**	4.00E-007	Up
A114373	LOC_Os03g33828.1	**2.029259921**	1.72E-006	Up
**A130353**	**LOC_Os06g48200.1**	**2.023550508**	**3.29E-006**	**Up**
A118829	LOC_Os04g25050.1	**2.019353462**	3.14E-006	Up
A139205	LOC_Os09g06790.1	**2.016049863**	1.80E-005	Up
**A126978**	**LOC_Os06g09240.1**	**2.010757051**	**5.14E-007**	**Up**
A132191	LOC_Os07g18260.1	**2.008701788**	1.10E-005	Up
A127328	LOC_Os06g12960.1	**1.99640988**	3.66E-005	Up
A130503	LOC_Os06g49820.1	**1.986763548**	3.12E-005	Up
A137674	LOC_Os08g33500.1	**1.983696529**	6.07E-006	Up
A143875	LOC_Os10g26870.1	**1.979037616**	1.52E-006	Up
A149025	LOC_Os11g46840.1	**1.978793707**	1.28E-006	Up
A113688	LOC_Os03g25210.1	**1.977575801**	0.000000364	Up
A106290	LOC_Os01g74450.1	**1.959320542**	5.04E-007	Up
A102236	LOC_Os01g27340.1	**1.945516457**	3.71E-006	Up
A136205	LOC_Os08g16100.1	**1.937232018**	5.48E-007	Up
A110318	LOC_Os02g46260.1	**1.932015007**	3.68E-006	Up
A139777	LOC_Os09g13550.1	**1.930584752**	1.09E-005	Up
A132450	LOC_Os07g22840.1	**1.918835622**	2.61E-006	Up
A146796	LOC_Os11g18961.1	**1.914261249**	2.13E-006	Up
A120115	LOC_Os04g40220.1	**1.911362788**	0.000754416	Up
A105656	LOC_Os01g67200.1	**1.907806872**	1.02E-006	Up
A114829	LOC_Os03g40610.1	**1.906625906**	1.71E-006	Up
A108400	LOC_Os02g24760.1	**1.904010709**	1.05E-005	Up
A130400	LOC_Os06g48750.1	**1.901052271**	5.33E-007	Up
A138096	LOC_Os08g37980.1	**1.90017826**	3.65E-006	Up
A151837	LOC_Os12g33050.1	**1.897145446**	0.002560403	Up
A146872	LOC_Os11g19764.1	**1.896170715**	0.000001568	Up
A127797	LOC_Os06g18670.1	**1.893692245**	6.24E-006	Up
A124172	LOC_Os05g28740.1	**1.886721099**	7.53E-007	Up
A127590	LOC_Os06g15840.1	**1.885176237**	4.34E-006	Up
A143287	LOC_Os10g19322.1	**1.879224616**	4.81E-005	Up
A122376	LOC_Os05g05990.1	**1.878080975**	0.000205122	Up

**A107753**	**LOC_Os02g16730.1**	**1.876275741**	**4.93E-007**	**Up**
A100115	LOC_Os01g02240.1	**1.874419311**	1.80E-005	Up
A137545	LOC_Os08g32130.2	**1.873958178**	5.88E-007	Up
A105190	LOC_Os01g62110.1	**1.872566348**	1.50E-006	Up
A119080	LOC_Os04g27950.1	**1.868843861**	8.06E-007	Up
A116524	LOC_Os03g60010.1	**1.862681608**	5.79E-006	Up
A150877	LOC_Os12g20370.1	**1.862514721**	0.000620297	Up
A150193	LOC_Os12g12210.1	**1.858530784**	1.53E-006	Up
A107094	LOC_Os02g09330.1	**1.840610522**	0.00161207	Up
A150598	LOC_Os12g16850.1	**1.826693672**	2.21E-006	Up
A109628	LOC_Os02g38790.1	**1.823069965**	7.88E-007	Up
A148933	LOC_Os11g45295.1	**1.817164856**	2.64E-006	Up
A136728	LOC_Os08g22852.1	**1.813372767**	0.003955531	Up
A133585	LOC_Os07g35920.2	**1.812258686**	0.000210468	Up
A149632	LOC_Os12g06040.1	**1.806666863**	5.29E-005	Up
A131977	LOC_Os07g15540.1	**1.804557436**	8.47E-006	Up
A128580	LOC_Os06g28590.1	**1.799537554**	1.08E-006	Up
A117750	LOC_Os04g11420.1	**1.799263694**	1.89E-005	Up
A125022	LOC_Os05g38660.1	**1.795813314**	0.000000627	Up
A102283	LOC_Os01g27890.1	**1.776906133**	2.11E-005	Up
A123271	LOC_Os05g16620.1	**1.771745272**	0.000302474	Up
A101040	LOC_Os01g12290.1	**1.771644244**	0.000005259	Up
A115376	LOC_Os03g47070.1	**1.7611047**	2.48E-005	Up
A131762	LOC_Os07g13060.1	**1.758475173**	4.95E-006	Up
A122025	LOC_Os05g02100.1	**1.757004081**	1.70E-005	Up
A137301	LOC_Os08g29400.1	**1.755930925**	0.000163443	Up
A102350	LOC_Os01g28680.1	**1.734002824**	2.73E-006	Up
A139621	LOC_Os09g11750.1	**1.728174651**	1.10E-005	Up
A102320	LOC_Os01g28300.1	**1.723666586**	3.07E-006	Up
A108659	LOC_Os02g27560.1	**1.698167349**	4.50E-006	Up
A135808	LOC_Os08g11310.1	**1.698046562**	0.000893408	Up
A109401	LOC_Os02g36290.1	**1.692989871**	0.000320662	Up
A129063	LOC_Os06g34510.1	**1.690710035**	3.69E-005	Up
A133221	LOC_Os07g31910.1	**1.680523264**	0.000613554	Up
A111948	LOC_Os03g05520.1	**1.658210848**	2.13E-005	Up
A152346	LOC_Os12g38500.1	**1.656668678**	1.70E-006	Up
A150630	LOC_Os12g17270.1	**1.656535826**	4.06E-006	Up
A116988	LOC_Os04g01730.1	**1.652291134**	1.18E-005	Up
A134630	LOC_Os07g47201.1	**1.644430287**	4.22E-006	Up
A131187	LOC_Os07g06834.1	**1.641212003**	0.001338994	Up
A129231	LOC_Os06g36390.1	**1.64075027**	0.000108482	Up
A116510	LOC_Os03g59870.1	**1.640337848**	5.96E-005	Up
A102631	LOC_Os01g33050.1	**1.635776675**	1.99E-006	Up
A101160	LOC_Os01g13570.1	**1.631002644**	0.00022034	Up
A122677	LOC_Os05g09300.1	**1.629470692**	9.60E-006	Up
A141844	LOC_Os09g38670.1	**1.628015203**	0.000314982	Up
A133112	LOC_Os07g30700.1	**1.620761631**	4.55E-005	Up
**A137238**	**LOC_Os08g28710.1**	**1.616729549**	**8.21E-005**	**Up**

A117157	LOC_Os04g03680.1	1.607763323	3.01E-006	Up
A141947	LOC_Os09g39710.1	1.602545739	8.86E-006	Up
A150486	LOC_Os12g15505.1	1.601704623	0.000408418	Up
**A119717**	**LOC_Os04g35020.1**	**1.600237103**	**0.001410464**	**Up**
A118696	LOC_Os04g23290.1	1.597686378	0.000713159	Up
A126064	LOC_Os05g50530.1	1.596421468	0.000215348	Up
A149717	LOC_Os12g06940.1	1.5930504	0.001732656	Up
A144863	LOC_Os10g38940.1	1.592603422	7.76E-005	Up
A148964	LOC_Os11g45650.1	1.590867112	0.000406135	Up
**A112001**	**LOC_Os03g06040.1**	**1.588399684**	**0.000239985**	**Up**
A115742	LOC_Os03g51090.1	1.57861801	2.44E-005	Up
A139758	LOC_Os09g13350.1	1.578006449	5.92E-006	Up
A116640	LOC_Os03g61360.1	1.573619622	2.22E-006	Up
A109021	LOC_Os02g32060.1	1.570946761	1.99E-005	Up
A101168	LOC_Os01g13660.1	1.569141559	1.68E-006	Up
A147728	LOC_Os11g31660.1	1.565654782	0.000206739	Up
A123178	LOC_Os05g15570.1	1.558972198	7.15E-005	Up
A116302	LOC_Os03g57600.1	1.556551197	3.99E-006	Up
A130798	LOC_Os07g02420.1	1.55599737	0.046840054	Up
A135561	LOC_Os08g08680.1	1.55591717	3.14E-005	Up
A133299	LOC_Os07g32740.1	1.552794616	0.001967062	Up
A119591	LOC_Os04g33640.1	1.54929328	1.40E-005	Up
A100686	LOC_Os01g08380.1	1.548933867	0.00000642	Up
A130557	LOC_Os06g50390.1	1.54605694	0.000255222	Up
A130602	LOC_Os06g50930.1	1.544692685	0.000135931	Up
A146631	LOC_Os11g16940.1	1.544206775	0.001432278	Up
A115091	LOC_Os03g43860.1	1.543324237	7.41E-006	Up
A137136	LOC_Os08g27580.1	1.541837356	0.009117942	Up
A139411	LOC_Os09g09160.1	1.541042892	8.73E-006	Up
A100141	LOC_Os01g02510.1	1.539288611	0.005712794	Up
A125200	LOC_Os05g40490.1	1.537660652	1.45E-005	Up
A137352	LOC_Os08g30014.1	1.535994317	7.09E-005	Up
A146330	LOC_Os11g12660.1	1.535024968	0.000549118	Up
A127661	LOC_Os06g16570.1	1.530855737	3.28E-006	Up
A105757	LOC_Os01g68290.1	1.525939219	0.000113859	Up
A131616	LOC_Os07g11420.1	1.52444988	5.86E-006	Up
A136453	LOC_Os08g19230.1	1.524275847	0.00000939	Up
A115760	LOC_Os03g51280.1	1.518101481	2.08E-005	Up
**A140727**	**LOC_Os09g25070.1**	**1.513096723**	**2.74E-005**	**Up**
A135274	LOC_Os08g05500.1	1.509503766	2.76E-005	Up
A129960	LOC_Os06g44140.1	1.508381077	1.75E-005	Up
A107683	LOC_Os02g15950.1	1.500434742	4.70E-005	Up
A140817	LOC_Os09g26020.1	1.499515013	4.65E-005	Up
A124385	LOC_Os05g31480.1	1.498781859	3.90E-006	Up
A120896	LOC_Os04g48590.1	1.497201609	0.000500576	Up
A118917	LOC_Os04g26040.1	1.496375616	0.001990494	Up
A114851	LOC_Os03g40960.1	1.495176667	1.58E-005	Up
A126210	LOC_Os06g01050.1	1.491514757	9.16E-005	Up

A140032	LOC_Os09g16400.1	**1.490061701**	0.00019174	Up
A111327	LOC_Os02g56790.1	**1.489055899**	0.000349588	Up
A116738	LOC_Os03g62370.1	**1.488496721**	6.05E-005	Up
A112808	LOC_Os03g15110.1	**1.488091478**	4.87E-005	Up
A150140	LOC_Os12g11570.1	**1.486044212**	6.20E-006	Up
A119238	LOC_Os04g29830.1	**1.484126603**	0.000603751	Up
A151315	LOC_Os12g26660.1	**1.483047647**	4.66E-006	Up
A128308	LOC_Os06g24420.1	**1.48198535**	0.00026657	Up
A127294	LOC_Os06g12580.1	**1.480774738**	2.88E-006	Up
A145573	LOC_Os11g04560.1	**1.473818903**	1.17E-005	Up
A134482	LOC_Os07g45640.1	**1.472935557**	0.000886558	Up
A129395	LOC_Os06g38210.2	**1.469531307**	8.48E-006	Up
A109632	LOC_Os02g38810.1	**1.468150898**	0.000315387	Up
A116939	LOC_Os04g01160.1	**1.466995517**	3.76E-006	Up
A136871	LOC_Os08g24520.1	**1.466228737**	0.000624919	Up
A148932	LOC_Os11g45290.1	**1.450525621**	0.001856683	Up
A106410	LOC_Os02g01970.1	**1.449118453**	0.016960446	Up
A101460	LOC_Os01g16870.1	**1.448235163**	5.24E-006	Up
A137224	LOC_Os08g28560.1	**1.445939634**	0.000218032	Up
A133685	LOC_Os07g37000.1	**1.445024171**	0.041495589	Up
A102761	LOC_Os01g34620.1	**1.444473835**	3.07E-006	Up
A112129	LOC_Os03g07410.3	**1.444455563**	0.000142121	Up
A100649	LOC_Os01g07950.1	**1.442674992**	2.43E-006	Up
A105007	LOC_Os01g60100.1	**1.439185063**	0.001437805	Up
A110542	LOC_Os02g48570.1	**1.438954341**	0.00000853	Up
A137193	LOC_Os08g28230.1	**1.437967984**	0.000005465	Up
A140351	LOC_Os09g20390.1	**1.434944025**	0.000344154	Up
A131522	LOC_Os07g10440.1	**1.434240565**	9.78E-006	Up
A147732	LOC_Os11g31700.1	**1.43150713**	9.99E-005	Up
A117055	LOC_Os04g02470.1	**1.428848182**	0.000084127	Up
A109177	LOC_Os02g33710.1	**1.428747093**	3.32E-006	Up
A132130	LOC_Os07g17500.1	**1.427289666**	4.28E-005	Up
A105496	LOC_Os01g65470.1	**1.425399704**	9.86E-005	Up
A117495	LOC_Os04g07990.1	**1.425016289**	8.79E-006	Up
A127572	LOC_Os06g15630.1	**1.42332335**	2.43E-006	Up
A138069	LOC_Os08g37690.1	**1.422096078**	0.000004982	Up
A128163	LOC_Os06g22750.1	**1.421825556**	0.000635436	Up
A127785	LOC_Os06g18010.1	**1.420212126**	0.000180775	Up
A140849	LOC_Os09g26350.1	**1.417627019**	2.08E-005	Up
A109986	LOC_Os02g42940.1	**1.414644737**	5.30E-006	Up
A135506	LOC_Os08g08084.1	**1.414335218**	0.002256437	Up
A121596	LOC_Os04g56309.1	**1.412017152**	2.47E-006	Up
A100657	LOC_Os01g08060.1	**1.411409691**	1.07E-005	Up
A111034	LOC_Os02g53660.1	**1.408636008**	7.06E-005	Up
A147830	LOC_Os11g32690.1	**1.408021357**	9.66E-006	Up
A137132	LOC_Os08g27540.1	**1.405865412**	0.000215956	Up
A105301	LOC_Os01g63340.1	**1.405090625**	0.000384061	Up
A142313	LOC_Os10g04770.1	**1.403333583**	5.19E-006	Up

A135967	LOC_Os08g13469.1	1.401654651	3.86E-005	Up
**A131066**	**LOC_Os07g05420.1**	**1.401029112**	**0.000640976**	**Up**
A101358	LOC_Os01g15770.1	1.400803252	1.90E-005	Up
A137778	LOC_Os08g34600.1	1.399448142	4.05E-005	Up
A132096	LOC_Os07g17150.1	1.398363934	0.002261558	Up
A120120	LOC_Os04g40270.1	1.396851461	5.72E-005	Up
A101360	LOC_Os01g15790.1	1.396230598	7.83E-005	Up
A104744	LOC_Os01g57350.2	1.394588166	0.048605333	Up
A137926	LOC_Os08g36080.1	1.394286316	1.94E-005	Up
A101955	LOC_Os01g23610.1	1.391477336	5.53E-006	Up
**A117282**	**LOC_Os04g05330.1**	**1.390133595**	**0.000731534**	**Up**
A102679	LOC_Os01g33640.1	1.387356872	2.20E-005	Up
A132308	LOC_Os07g20164.1	1.384340691	7.05E-005	Up
A108650	LOC_Os02g27460.1	1.383924708	0.000807024	Up
A151608	LOC_Os12g30420.1	1.383597838	0.000761812	Up
A112862	LOC_Os03g15680.1	1.38321884	0.011331663	Up
A122618	LOC_Os05g08680.1	1.379240444	0.00016195	Up
A127728	LOC_Os06g17340.1	1.376068125	1.89E-005	Up
A110467	LOC_Os02g47840.1	1.376053142	4.02E-006	Up
A136392	LOC_Os08g18150.1	1.370190675	0.000057706	Up
A131226	LOC_Os07g07280.1	1.365520254	0.0001327	Up
A102967	LOC_Os01g37280.1	1.364313657	0.000183996	Up
A125733	LOC_Os05g46630.1	1.362861251	0.000426105	Up
A116643	LOC_Os03g61390.1	1.36136959	4.05E-005	Up
A138521	LOC_Os08g42430.1	1.361224436	0.000106989	Up
A110182	LOC_Os02g44820.1	1.359836796	3.47E-006	Up
A117943	LOC_Os04g13670.1	1.359459335	3.37E-005	Up
A133483	LOC_Os07g34770.1	1.359269392	0.014736897	Up
A122023	LOC_Os05g02070.2	1.35755249	3.61E-006	Up
A115612	LOC_Os03g49600.1	1.357297479	4.35E-006	Up
A101597	LOC_Os01g18900.1	1.356447102	1.73E-005	Up
A136282	LOC_Os08g16880.1	1.354756875	1.42E-005	Up
A100858	LOC_Os01g10210.1	1.35468634	0.000736754	Up
A137274	LOC_Os08g29100.1	1.352853574	0.000273566	Up
A102776	LOC_Os01g34790.1	1.351275059	0.000233273	Up
A131054	LOC_Os07g05290.1	1.349271093	0.019042332	Up
A109798	LOC_Os02g40460.1	1.345018347	4.36E-006	Up
A101905	LOC_Os01g22910.1	1.344412396	0.024812188	Up
A137297	LOC_Os08g29360.1	1.34217876	0.000394696	Up
A132185	LOC_Os07g18180.1	1.341305827	6.11E-005	Up
A132498	LOC_Os07g23430.1	1.340424843	0.00021771	Up
A139595	LOC_Os09g11400.1	1.337577361	0.000145764	Up
A111588	LOC_Os03g01620.1	1.335728497	0.000422954	Up
A133415	LOC_Os07g34080.1	1.333510691	0.000103345	Up
A127081	LOC_Os06g10360.1	1.332342989	0.001186489	Up
A135700	LOC_Os08g10220.1	1.330122761	2.13E-005	Up
A134448	LOC_Os07g45210.1	1.328453218	0.002206123	Up
**A102181**	**LOC_Os01g26174.1**	**1.32499543**	**6.84E-006**	**Up**

A107841	LOC_Os02g17650.1	1.32186243	1.41E-005	Up
A129884	LOC_Os06g43370.1	1.320602846	9.22E-006	Up
A103172	LOC_Os01g39660.1	1.320031119	0.031771229	Up
**A135709**	**LOC_Os08g10300.1**	**1.31926416**	**0.000369106**	**Up**
A119448	LOC_Os04g32140.1	1.318812517	5.73E-006	Up
A117044	LOC_Os04g02360.1	1.317973643	0.001689267	Up
A132255	LOC_Os07g19060.1	1.317951244	1.44E-005	Up
A151392	LOC_Os12g27640.1	1.315125361	7.45E-005	Up
A106084	LOC_Os01g72260.1	1.315086878	1.27E-005	Up
A135976	LOC_Os08g13640.1	1.314346774	8.65E-005	Up
**A102187**	**LOC_Os01g26280.1**	**1.313801211**	**0.0011778**	**Up**
A101695	LOC_Os01g20030.1	1.313647952	7.96E-006	Up
A133459	LOC_Os07g34529.1	1.312549724	0.02769103	Up
A149864	LOC_Os12g08480.1	1.310764638	0.033922473	Up
A101457	LOC_Os01g16840.1	1.309131659	0.001745169	Up
A135929	LOC_Os08g13060.1	1.308529786	0.000004506	Up
A135692	LOC_Os08g10100.1	1.304623635	2.22E-005	Up
A102842	LOC_Os01g35780.1	1.304441962	0.05985816	Up
A148046	LOC_Os11g35480.1	1.302652859	8.14E-005	Up
A133805	LOC_Os07g38270.1	1.302292956	1.48E-005	Up
A136799	LOC_Os08g23700.1	1.300455844	6.07E-005	Up
A115432	LOC_Os03g47640.1	1.300428633	0.000435372	Up
A135436	LOC_Os08g07320.1	1.299912704	0.000167773	Up
A123083	LOC_Os05g14450.1	1.298433089	1.54E-005	Up
A102215	LOC_Os01g27090.1	1.296902436	4.33E-005	Up
A109184	LOC_Os02g33790.1	1.296198584	0.000077795	Up
**A100319**	**LOC_Os01g04450.1**	**1.295885386**	**6.39E-006**	**Up**
A151582	LOC_Os12g30050.1	1.295759505	0.000106206	Up
**A106462**	**LOC_Os02g02540.1**	**1.294970798**	**0.000389072**	**Up**
A128867	LOC_Os06g32160.1	1.293850144	6.11E-006	Up
A132633	LOC_Os07g25180.1	1.292052701	4.90E-005	Up
A102146	LOC_Os01g25710.1	1.290370215	1.04E-005	Up
A103972	LOC_Os01g49020.1	1.288396833	0.001085318	Up
A115360	LOC_Os03g46910.1	1.286182168	0.000347726	Up
A135474	LOC_Os08g07720.1	1.286107826	5.68E-006	Up
A151427	LOC_Os12g28090.1	1.283140815	0.018756139	Up
A134823	LOC_Os07g49210.1	1.283118535	0.041304285	Up
A113722	LOC_Os03g25490.1	1.282091545	4.76E-005	Up
A120543	LOC_Os04g44840.1	1.281078904	0.000285496	Up
A107884	LOC_Os02g18090.1	1.281036222	5.95E-006	Up
A141821	LOC_Os09g38440.1	1.275544185	9.80E-005	Up
A144391	LOC_Os10g33470.1	1.275282075	6.24E-005	Up
A134087	LOC_Os07g41290.1	1.273216952	0.000671609	Up
A117270	LOC_Os04g05130.1	1.272683635	0.026334173	Up
A117915	LOC_Os04g13364.1	1.270110221	0.000670086	Up
A140317	LOC_Os09g20040.1	1.268869177	0.000796979	Up
A100889	LOC_Os01g10580.1	1.268503283	6.63E-006	Up
A151378	LOC_Os12g27480.1	1.267755116	0.003476157	Up

A130522	LOC_Os06g50020.1	1.266186625	0.000067456	Up
A150343	LOC_Os12g13760.1	1.263040493	5.46E-005	Up
A142853	LOC_Os10g11520.1	1.26247601	2.84E-005	Up
A116796	LOC_Os03g63010.1	1.259032023	0.000179577	Up
A130946	LOC_Os07g04120.1	1.258981409	0.044717797	Up
**A124920**	**LOC_Os05g37620.1**	**1.257137405**	**0.026215172**	**Up**
A147725	LOC_Os11g31630.1	1.25562227	1.04E-005	Up
A146765	LOC_Os11g18640.1	1.253964902	2.38E-005	Up
A139601	LOC_Os09g11470.1	1.253785258	0.000205931	Up
A133600	LOC_Os07g36080.1	1.248354885	7.42E-006	Up
A108431	LOC_Os02g25090.1	1.242974319	0.003002753	Up
A146426	LOC_Os11g14220.1	1.242371768	7.75E-006	Up
A148650	LOC_Os11g42170.1	1.240229861	1.15E-005	Up
A100293	LOC_Os01g04160.1	1.238179303	0.000436582	Up
A115977	LOC_Os03g53710.1	1.237758251	0.000163693	Up
A150973	LOC_Os12g22460.1	1.237476461	7.86E-006	Up
A143320	LOC_Os10g20060.1	1.232372085	0.001451758	Up
A100872	LOC_Os01g10380.1	1.229132368	4.49E-005	Up
A147346	LOC_Os11g27060.1	1.227560808	0.018035136	Up
A149621	LOC_Os12g05920.1	1.226658455	5.61E-006	Up
A150383	LOC_Os12g14190.1	1.224709797	0.000284714	Up
A101461	LOC_Os01g16890.1	1.224045145	0.000006726	Up
**A146729**	**LOC_Os11g18170.1**	**1.22369514**	**5.34E-005**	**Up**
A147951	LOC_Os11g34450.1	1.223605099	6.99E-006	Up
A124123	LOC_Os05g28150.1	1.22353448	7.91E-005	Up
A150629	LOC_Os12g17260.1	1.222928435	0.000323863	Up
A131582	LOC_Os07g11040.1	1.222037708	0.000052848	Up
A144917	LOC_Os10g39500.1	1.220440386	0.000145143	Up
**A149040**	**LOC_Os11g47000.1**	**1.21922524**	**0.044279781**	**Up**
A140551	LOC_Os09g22550.1	1.217287388	1.79E-005	Up
A119430	LOC_Os04g31950.1	1.214616124	3.62E-005	Up
A101522	LOC_Os01g18070.1	1.214605109	8.17E-006	Up
A152603	LOC_Os12g41230.1	1.213797413	1.98E-005	Up
A126840	LOC_Os06g07780.1	1.212907511	0.000798156	Up
**A137999**	**LOC_Os08g36910.1**	**1.209296997**	**6.99E-005**	**Up**
A136076	LOC_Os08g14770.1	1.209241055	5.94E-005	Up
A124238	LOC_Os05g29880.3	1.208991345	0.000057311	Up
A137419	LOC_Os08g30760.1	1.207821705	0.001345054	Up
A122670	LOC_Os05g09230.1	1.207122849	0.000157911	Up
A100575	LOC_Os01g07170.1	1.206122669	9.02E-006	Up
A117999	LOC_Os04g14390.1	1.205795046	6.91E-006	Up
A138079	LOC_Os08g37790.1	1.205590223	8.76E-006	Up
A116268	LOC_Os03g57220.1	1.20348503	7.64E-005	Up
A121878	LOC_Os04g59260.1	1.202970837	0.000007137	Up
A147634	LOC_Os11g30546.1	1.200904011	0.00011677	Up
A147140	LOC_Os11g24610.1	1.200263509	0.000013524	Up
A150791	LOC_Os12g19190.1	1.200250763	0.004557161	Up
A107414	LOC_Os02g13140.1	1.199998618	0.000091164	Up

A129402	LOC_Os06g38294.1	**1.199616575**	8.54E-006	Up
A103742	LOC_Os01g46540.1	**1.199586876**	5.68E-005	Up
A124622	LOC_Os05g33960.1	**1.199116717**	0.000132221	Up
A112356	LOC_Os03g10290.1	**1.197091747**	0.035952335	Up
A100721	LOC_Os01g08720.1	**1.197080203**	0.000892034	Up
A134140	LOC_Os07g41870.1	**1.197042211**	0.000227215	Up
A115122	LOC_Os03g44220.1	**1.196242102**	0.041990647	Up
A103254	LOC_Os01g40600.1	**1.19449366**	1.81E-005	Up
A102683	LOC_Os01g33700.1	**1.194347965**	0.044207042	Up
A113633	LOC_Os03g24610.1	**1.192529386**	0.000367896	Up
A103159	LOC_Os01g39500.1	**1.192399179**	0.000023196	Up
A108936	LOC_Os02g30650.1	**1.191928027**	2.09E-005	Up
A138348	LOC_Os08g40610.1	**1.191552714**	2.18E-005	Up
A134299	LOC_Os07g43604.1	**1.191217759**	0.000011725	Up
A110844	LOC_Os02g51640.1	**1.189186765**	0.000128569	Up
A135727	LOC_Os08g10470.1	**1.189001126**	1.85E-005	Up
A133207	LOC_Os07g31720.1	**1.188484944**	1.66E-005	Up
A111320	LOC_Os02g56720.2	**1.188103571**	3.04E-005	Up
A102432	LOC_Os01g29700.1	**1.186667791**	5.39E-005	Up
A138743	LOC_Os08g44780.1	**1.185702365**	5.72E-005	Up
A109716	LOC_Os02g39630.1	**1.185657851**	9.22E-006	Up
A129804	LOC_Os06g42510.1	**1.184524942**	0.000304262	Up
A152893	LOC_Os12g44310.1	**1.179243124**	9.28E-006	Up
A107508	LOC_Os02g14110.1	**1.177501825**	1.18E-005	Up
A138248	LOC_Os08g39560.1	**1.177210052**	0.006822429	Up
A118964	LOC_Os04g26550.1	**1.173906154**	0.059844426	Up
A147741	LOC_Os11g31770.1	**1.171887872**	0.009339887	Up
A141853	LOC_Os09g38755.1	**1.171765281**	0.001747346	Up
A124736	LOC_Os05g35160.1	**1.171624164**	0.044676982	Up
A132420	LOC_Os07g22494.1	**1.171485719**	0.000433075	Up
A150419	LOC_Os12g14640.1	**1.17043347**	0.000102714	Up
A135897	LOC_Os08g12710.1	**1.170313002**	1.34E-005	Up
A152370	LOC_Os12g38760.1	**1.16950754**	0.001438765	Up
A129865	LOC_Os06g43150.1	**1.168683242**	0.000120734	Up
A141659	LOC_Os09g36760.1	**1.168115093**	0.0002001	Up
A142028	LOC_Os10g01510.1	**1.16643382**	0.001420626	Up
A138421	LOC_Os08g41360.1	**1.166424021**	0.039537237	Up
A128586	LOC_Os06g28680.1	**1.166010946**	0.003498203	Up
A144113	LOC_Os10g30070.1	**1.165309682**	4.27E-005	Up
**A115370**	**LOC_Os03g47022.1**	**1.164424934**	**0.022588817**	**Up**
A119673	LOC_Os04g34530.1	**1.164303413**	0.000191697	Up
A150341	LOC_Os12g13740.1	**1.159613151**	0.003241459	Up
A117737	LOC_Os04g11260.1	**1.158227987**	0.000104416	Up
A139220	LOC_Os09g06970.1	**1.156377874**	0.000108747	Up
A108107	LOC_Os02g20590.1	**1.1555509**	0.000315872	Up
A129818	LOC_Os06g42650.1	**1.155195984**	0.026091086	Up
A129944	LOC_Os06g43980.1	**1.155147257**	0.000101192	Up
A139787	LOC_Os09g13670.1	**1.154519536**	0.000124292	Up

A115433	LOC_Os03g47650.1	1.152939073	0.001295209	Up
A105875	LOC_Os01g70040.1	1.152468259	5.76E-005	Up
A130651	LOC_Os06g51460.1	1.152148362	0.00024492	Up
A144762	LOC_Os10g37660.1	1.150608576	3.92E-005	Up
A144967	LOC_Os10g40000.1	1.148969481	0.036436734	Up
A128861	LOC_Os06g32020.1	1.148933908	0.000237795	Up
A141206	LOC_Os09g30160.1	1.147560079	1.62E-005	Up
A151080	LOC_Os12g23840.1	1.146654231	0.018786496	Up
A128977	LOC_Os06g33490.1	1.143944271	0.000966429	Up
A137366	LOC_Os08g30160.1	1.143598501	0.002799602	Up
A151885	LOC_Os12g33630.1	1.142602277	0.000873826	Up
A113225	LOC_Os03g19520.2	1.142352556	8.97E-005	Up
A124927	LOC_Os05g37690.1	1.14185185	1.51E-005	Up
A118186	LOC_Os04g17030.1	1.141478249	0.031679801	Up
A122135	LOC_Os05g03290.1	1.141466129	2.34E-005	Up
A134447	LOC_Os07g45195.1	1.141065214	2.19E-005	Up
A120185	LOC_Os04g40950.1	1.138924623	8.75E-005	Up
A109827	LOC_Os02g40784.1	1.13842925	0.000134229	Up
A126992	LOC_Os06g09390.1	1.138067328	1.47E-005	Up
**A133006**	**LOC_Os07g29490.1**	**1.137828229**	**0.016493531**	**Up**
A123773	LOC_Os05g24110.1	1.137819678	2.89E-005	Up
A114483	LOC_Os03g36500.1	1.137514639	3.72E-005	Up
A126886	LOC_Os06g08310.1	1.136852154	0.002678264	Up
A101104	LOC_Os01g12950.1	1.136759567	0.003058581	Up
A101933	LOC_Os01g23380.1	1.136216935	0.000134686	Up
A121333	LOC_Os04g53520.1	1.135758183	1.75E-005	Up
A151953	LOC_Os12g34250.1	1.134764929	0.000529238	Up
A127813	LOC_Os06g18860.1	1.133726116	0.000551311	Up
A117066	LOC_Os04g02590.1	1.133501679	0.007806931	Up
A118460	LOC_Os04g20440.1	1.129888002	0.00036139	Up
A142642	LOC_Os10g08780.1	1.12873893	1.45E-005	Up
A115514	LOC_Os03g48480.1	1.128431301	0.000023072	Up
A125375	LOC_Os05g42330.1	1.127363475	0.000236407	Up
A137406	LOC_Os08g30610.1	1.126836458	9.23E-005	Up
A105108	LOC_Os01g61230.1	1.126828982	0.000101494	Up
A106400	LOC_Os02g01870.1	1.126520407	3.93E-005	Up
A141874	LOC_Os09g38960.2	1.125608669	0.001016545	Up
A100033	LOC_Os01g01340.1	1.125139014	5.27E-005	Up
**A133642**	**LOC_Os07g36544.2**	**1.124818938**	**0.013011879**	**Up**
**A132862**	**LOC_Os07g27900.1**	**1.122078791**	**0.004882734**	**Up**
A116077	LOC_Os03g55310.1	1.120829293	9.96E-006	Up
A122797	LOC_Os05g10980.1	1.120122053	0.000204347	Up
A151447	LOC_Os12g28420.1	1.120117881	0.002236661	Up
A132381	LOC_Os07g22070.1	1.118898178	2.54E-005	Up
A134130	LOC_Os07g41770.1	1.117962661	5.63E-005	Up
A129941	LOC_Os06g43950.1	1.117581395	1.88E-005	Up
A128853	LOC_Os06g31890.1	1.117468936	2.04E-005	Up
A127532	LOC_Os06g15200.1	1.115664482	0.000440749	Up

**A117583**	**LOC_Os04g09390.1**	**1.115608844**	**1.87E-005**	**Up**
A107307	LOC_Os02g12020.1	1.115565682	0.018776022	Up
A146458	LOC_Os11g14530.1	1.114276139	8.08E-005	Up
A107371	LOC_Os02g12680.1	1.112607707	0.035352728	Up
A136184	LOC_Os08g15890.1	1.111219002	0.036597877	Up
A146340	LOC_Os11g12770.1	1.110746385	4.50E-005	Up
A111916	LOC_Os03g05210.1	1.109524049	0.000344664	Up
A109664	LOC_Os02g39120.1	1.109370173	0.000124885	Up
A128124	LOC_Os06g22350.1	1.107674182	0.006850339	Up
A128129	LOC_Os06g22394.1	1.107649354	0.006962987	Up
A105022	LOC_Os01g60260.1	1.107624489	0.004695446	Up
A110913	LOC_Os02g52360.1	1.107253782	0.000116707	Up
A126861	LOC_Os06g08014.1	1.106886738	9.79E-005	Up
A102137	LOC_Os01g25610.1	1.10662452	2.80E-005	Up
A115595	LOC_Os03g49440.1	1.106365942	1.60E-005	Up
A130799	LOC_Os07g02430.1	1.105455988	0.000511374	Up
A132334	LOC_Os07g20460.1	1.104148257	3.40E-005	Up
A132804	LOC_Os07g27190.1	1.103170146	0.002537558	Up
A119090	LOC_Os04g28060.1	1.102224477	2.15E-005	Up
A149289	LOC_Os12g02290.1	1.099626588	2.23E-005	Up
A102765	LOC_Os01g34680.1	1.096633187	0.036372927	Up
A148102	LOC_Os11g36100.1	1.09638294	0.000107148	Up
A126743	LOC_Os06g06760.1	1.095913074	0.000364678	Up
A126552	LOC_Os06g04830.1	1.094723877	0.000452073	Up
A128350	LOC_Os06g24930.1	1.094042201	0.000266595	Up
A130868	LOC_Os07g03130.1	1.093280286	1.74E-005	Up
A115526	LOC_Os03g48642.1	1.089519173	0.000152613	Up
A106854	LOC_Os02g06470.1	1.088552128	0.000350762	Up
A123951	LOC_Os05g26090.1	1.086961765	4.34E-005	Up
A115498	LOC_Os03g48320.1	1.085958399	0.000138923	Up
A133601	LOC_Os07g36090.1	1.085727733	2.70E-005	Up
A116623	LOC_Os03g61160.1	1.085638153	0.002177664	Up
A118274	LOC_Os04g18090.1	1.085419792	0.000012827	Up
A128914	LOC_Os06g32710.1	1.085359738	0.000843666	Up
A134967	LOC_Os08g02140.2	1.085036808	2.68E-005	Up
A144775	LOC_Os10g37810.1	1.085034684	0.000123143	Up
A116637	LOC_Os03g61310.1	1.084622491	0.000983073	Up
A122538	LOC_Os05g07830.1	1.084182591	0.007849287	Up
A123561	LOC_Os05g20620.1	1.083872619	0.000516248	Up
A127737	LOC_Os06g17450.1	1.083754332	8.78E-005	Up
A104635	LOC_Os01g56150.1	1.082948381	0.00004116	Up
A122977	LOC_Os05g13380.1	1.082123865	0.000458908	Up
A127319	LOC_Os06g12876.1	1.081493934	0.013265897	Up
A152090	LOC_Os12g35730.1	1.081012537	0.003441243	Up
A119709	LOC_Os04g34930.1	1.080263951	1.12E-005	Up
A125285	LOC_Os05g41400.1	1.07967899	0.029169568	Up
A143388	LOC_Os10g20870.1	1.07965956	0.010749233	Up
A117079	LOC_Os04g02730.1	1.079622456	2.97E-005	Up

A141856	LOC_Os09g38772.1	1.079200644	1.74E-005	Up
**A136108**	**LOC_Os08g15090.1**	**1.078413825**	**0.001092045**	**Up**
A141839	LOC_Os09g38620.1	1.078367954	1.98E-005	Up
A141181	LOC_Os09g29890.1	1.077840415	1.85E-005	Up
A126896	LOC_Os06g08420.1	1.07612058	0.009078631	Up
**A141372**	**LOC_Os09g32510.1**	**1.075691096**	**0.000030025**	**Up**
A115936	LOC_Os03g53270.1	1.074484975	2.16E-005	Up
A132977	LOC_Os07g29170.1	1.074213725	0.002198794	Up
A145989	LOC_Os11g08840.1	1.073829746	0.054576223	Up
A107040	LOC_Os02g08300.1	1.072917664	1.78E-005	Up
A126317	LOC_Os06g02260.1	1.072613509	7.28E-005	Up
A109453	LOC_Os02g36850.1	1.072570296	0.000340145	Up
A131400	LOC_Os07g09180.1	1.070847657	0.000236456	Up
A109199	LOC_Os02g34000.1	1.070224136	0.000155569	Up
A108732	LOC_Os02g28380.1	1.069149337	0.001813282	Up
A146987	LOC_Os11g22250.1	1.068876752	4.43E-005	Up
A107705	LOC_Os02g16220.1	1.06844309	0.000298544	Up
A129004	LOC_Os06g33820.1	1.066913452	0.000102865	Up
A137280	LOC_Os08g29170.1	1.06573098	0.000139512	Up
A139143	LOC_Os09g05120.1	1.065420466	0.000200108	Up
A103902	LOC_Os01g48260.1	1.064394427	8.82E-005	Up
A149828	LOC_Os12g08100.1	1.062852352	0.001220266	Up
A126004	LOC_Os05g49900.1	1.062306321	0.000183943	Up
A136189	LOC_Os08g15940.1	1.062288453	0.000518631	Up
A110860	LOC_Os02g51810.1	1.060489213	0.000293493	Up
A126470	LOC_Os06g03930.1	1.059734717	0.000119991	Up
A132309	LOC_Os07g20170.1	1.058560085	0.000213132	Up
A123494	LOC_Os05g19750.1	1.053332872	4.71E-005	Up
A142602	LOC_Os10g08350.1	1.052891397	0.000148108	Up
A113305	LOC_Os03g20400.1	1.0515441	0.009205664	Up
A121526	LOC_Os04g55555.1	1.051360923	0.000212713	Up
A130070	LOC_Os06g45250.1	1.051271303	0.000122853	Up
A135118	LOC_Os08g03770.1	1.051009665	0.017314946	Up
A110219	LOC_Os02g45210.1	1.050046608	0.001109644	Up
A137172	LOC_Os08g28010.1	1.048696555	0.001043766	Up
A124279	LOC_Os05g30350.1	1.048029962	1.96E-005	Up
A124062	LOC_Os05g27470.1	1.047744628	0.000126234	Up
A103131	LOC_Os01g39136.1	1.047475795	4.93E-005	Up
A119041	LOC_Os04g27470.1	1.047322477	0.008077213	Up
A137567	LOC_Os08g32360.1	1.046459609	0.030571678	Up
A136579	LOC_Os08g20780.1	1.045050481	2.19E-005	Up
A128391	LOC_Os06g25470.1	1.044999079	0.000243714	Up
A100873	LOC_Os01g10390.1	1.044267546	0.00036367	Up
A109018	LOC_Os02g32030.1	1.044089742	0.000023963	Up
A115761	LOC_Os03g51290.1	1.04401237	0.000105405	Up
A152787	LOC_Os12g43150.1	1.043667533	0.010448217	Up
A109346	LOC_Os02g35700.1	1.043170186	2.57E-005	Up
A118417	LOC_Os04g19870.1	1.043166115	0.00011321	Up

A130555	LOC_Os06g50370.1	**1.042570354**	2.86E-005	Up
A109412	LOC_Os02g36400.1	**1.041029238**	0.000044427	Up
A147486	LOC_Os11g28840.1	**1.040704461**	3.44E-005	Up
A100770	LOC_Os01g09260.1	**1.040338025**	0.000680419	Up
A122087	LOC_Os05g02750.1	**1.039567531**	5.58E-005	Up
A121710	LOC_Os04g57500.1	**1.038449541**	7.67E-005	Up
A131647	LOC_Os07g11810.1	**1.037458665**	3.78E-005	Up
A141832	LOC_Os09g38550.1	**1.036404306**	4.31E-005	Up
A101458	LOC_Os01g16850.1	**1.03574804**	0.000113688	Up
A133968	LOC_Os07g39980.1	**1.034775304**	0.000860558	Up
A100238	LOC_Os01g03570.1	**1.034330891**	0.00041106	Up
A112088	LOC_Os03g06980.1	**1.034206635**	2.85E-005	Up
A109624	LOC_Os02g38750.1	**1.033431079**	2.47E-005	Up
A111062	LOC_Os02g53940.1	**1.032977028**	0.000267453	Up
A120951	LOC_Os04g49270.1	**1.031721086**	0.000667285	Up
A111924	LOC_Os03g05290.1	**1.030301793**	2.36E-005	Up
A115239	LOC_Os03g45519.1	**1.029811277**	0.000245672	Up
A120959	LOC_Os04g49370.1	**1.029220521**	0.000141599	Up
A138784	LOC_Os08g45210.1	**1.028775409**	7.35E-005	Up
A133859	LOC_Os07g38830.1	**1.028736922**	0.000073677	Up
A104805	LOC_Os01g57954.1	**1.028433842**	3.90E-005	Up
A116622	LOC_Os03g61150.1	**1.027506687**	8.98E-005	Up
A107625	LOC_Os02g15340.1	**1.027083693**	0.016705464	Up
A109605	LOC_Os02g38490.1	**1.025763825**	0.00025393	Up
A141439	LOC_Os09g33555.1	**1.024971771**	0.005768635	Up
A146009	LOC_Os11g09070.1	**1.024939365**	0.050844152	Up
A109746	LOC_Os02g39930.1	**1.024600898**	3.58E-005	Up
A133345	LOC_Os07g33240.1	**1.023607737**	5.84E-005	Up
A120556	LOC_Os04g44970.1	**1.02323156**	0.000145469	Up
A103728	LOC_Os01g46380.1	**1.02226668**	2.02E-005	Up
A143559	LOC_Os10g22650.1	**1.022232694**	0.00031101	Up
A116454	LOC_Os03g59290.1	**1.022156611**	2.18E-005	Up
A106449	LOC_Os02g02400.1	**1.022107439**	8.46E-005	Up
A123428	LOC_Os05g18880.1	**1.021836183**	0.001786729	Up
A145357	LOC_Os11g02330.1	**1.020799921**	0.000047574	Up
A113911	LOC_Os03g27730.1	**1.019650204**	0.046440981	Up
A118463	LOC_Os04g20474.1	**1.01924333**	0.000301669	Up
A124676	LOC_Os05g34530.1	**1.01867214**	0.00046425	Up
A108678	LOC_Os02g27760.1	**1.018609884**	2.60E-005	Up
A116415	LOC_Os03g58850.1	**1.018537094**	3.28E-005	Up
A132593	LOC_Os07g24569.1	**1.018417987**	9.39E-005	Up
A113677	LOC_Os03g25090.1	**1.016997415**	0.010458138	Up
A151493	LOC_Os12g28980.1	**1.016877819**	0.000162315	Up
A143553	LOC_Os10g22590.1	**1.016325094**	1.79E-005	Up
A126001	LOC_Os05g49870.1	**1.015753761**	0.008881531	Up
A142513	LOC_Os10g07300.1	**1.015650854**	0.005988824	Up
A146451	LOC_Os11g14470.1	**1.015543323**	0.001563749	Up
A119100	LOC_Os04g28180.1	**1.015180754**	1.91E-005	Up

A145438	LOC_Os11g03170.1	1.01427679	6.70E-005	Up
A124879	LOC_Os05g37170.1	1.013848635	3.16E-005	Up
A136884	LOC_Os08g24710.1	1.012981499	0.00482314	Up
A111779	LOC_Os03g03720.1	1.012953331	0.007332999	Up
A132319	LOC_Os07g20290.1	1.012762836	4.25E-005	Up
A125286	LOC_Os05g41410.1	1.011296518	0.009594089	Up
A139212	LOC_Os09g06890.1	1.011278842	0.000244843	Up
A150536	LOC_Os12g16170.1	1.009830516	0.00014674	Up
A118691	LOC_Os04g23240.1	1.009302767	0.000398969	Up
A150300	LOC_Os12g13330.1	1.008372602	0.005941073	Up
A143556	LOC_Os10g22620.1	1.008156467	0.000277691	Up
A139867	LOC_Os09g14614.1	1.007731168	0.000366171	Up
A129600	LOC_Os06g40360.1	1.006157742	5.31E-005	Up
A118730	LOC_Os04g23710.1	1.004545738	0.05476206	Up
A110200	LOC_Os02g45000.1	1.004117	1.76E-005	Up
A112946	LOC_Os03g16580.1	1.003276086	0.000157329	Up
A130797	LOC_Os07g02410.1	1.002531843	0.055562322	Up
A143500	LOC_Os10g21970.1	1.001482319	0.038480731	Up
A122338	LOC_Os05g05600.1	1.001148369	2.13E-005	Up
A123700	LOC_Os05g23310.1	1.00107849	9.98E-005	Up
A114070	LOC_Os03g30084.1	1.000849532	0.01331668	Up
A130814	LOC_Os07g02590.1	-1.000036035	0.009221233	Down
A137264	LOC_Os08g28970.1	-1.000132683	2.55E-005	Down
A144823	LOC_Os10g38489.1	-1.000156507	2.42E-005	Down
A116595	LOC_Os03g60850.1	-1.000448806	9.32E-005	Down
A119539	LOC_Os04g33115.1	-1.00107722	6.94E-005	Down
A101114	LOC_Os01g13070.1	-1.00136127	0.000658637	Down
A138138	LOC_Os08g38410.1	-1.002157021	0.005537852	Down
A132709	LOC_Os07g26030.1	-1.002183076	0.011098099	Down
A102498	LOC_Os01g31450.1	-1.002584037	0.002313299	Down
A128987	LOC_Os06g33620.1	-1.004378077	0.038722439	Down
A123296	LOC_Os05g16990.1	-1.004511765	0.00489107	Down
A121356	LOC_Os04g53750.1	-1.004940399	0.000507189	Down
A100102	LOC_Os01g02080.1	-1.005733063	0.005863955	Down
A130842	LOC_Os07g02880.1	-1.00708533	0.000259406	Down
A107387	LOC_Os02g12840.1	-1.008093794	8.41E-005	Down
A130777	LOC_Os07g02192.1	-1.008253212	3.83E-005	Down
**A143467**	**LOC_Os10g21590.1**	-1.009028679	**1.92E-005**	**Down**
A145703	LOC_Os11g05830.1	-1.009281838	0.000171208	Down
A107032	LOC_Os02g08220.1	-1.010202765	0.00056097	Down
A131462	LOC_Os07g09850.1	-1.010897823	0.040239961	Down
A151470	LOC_Os12g28720.1	-1.013048193	0.002121546	Down
A131607	LOC_Os07g11330.1	-1.013304977	4.64E-005	Down
A150232	LOC_Os12g12640.1	-1.013723992	0.007852363	Down
A113279	LOC_Os03g20110.1	-1.013832633	0.001173454	Down
**A119911**	**LOC_Os04g38026.1**	-1.01407436	**3.59E-005**	**Down**
A113804	LOC_Os03g26490.1	-1.014412917	1.84E-005	Down
A142523	LOC_Os10g07430.1	-1.01442606	0.00019757	Down

A117527	LOC_Os04g08415.1	-1.014703189	0.004350558	Down
A108589	LOC_Os02g26794.1	-1.015154819	0.006747268	Down
A131656	LOC_Os07g11910.1	-1.015395385	1.68E-005	Down
A144808	LOC_Os10g38189.1	-1.01551437	0.001304829	Down
A138270	LOC_Os08g39800.1	-1.015554335	1.82E-005	Down
A142829	LOC_Os10g11180.1	-1.015622935	0.000346492	Down
**A147573**	**LOC_Os11g29870.1**	-1.016501101	**4.01E-005**	**Down**
A111399	LOC_Os02g57520.1	-1.016974756	0.011402463	Down
A111942	LOC_Os03g05460.1	-1.017115536	0.000926516	Down
A146732	LOC_Os11g18230.1	-1.017586202	0.002270944	Down
A106061	LOC_Os01g72009.1	-1.019413908	1.98E-005	Down
A105465	LOC_Os01g65100.1	-1.020353643	9.91E-005	Down
A133877	LOC_Os07g39020.1	-1.020437582	0.000611689	Down
**A117115**	**LOC_Os04g03180.1**	-1.020991206	**0.008418187**	**Down**
A128522	LOC_Os06g27910.1	-1.021427049	2.06E-005	Down
**A124868**	**LOC_Os05g37060.1**	-1.021484202	**5.44E-005**	**Down**
A109574	LOC_Os02g38140.1	-1.021974185	0.000845316	Down
A112148	LOC_Os03g07620.1	-1.023734846	0.003110565	Down
A119883	LOC_Os04g37700.1	-1.024168215	0.00012641	Down
**A121133**	**LOC_Os04g51460.1**	-1.024617227	**0.001513431**	**Down**
A152337	LOC_Os12g38410.1	-1.02462781	0.000215704	Down
A109483	LOC_Os02g37180.1	-1.024963187	2.57E-005	Down
A152657	LOC_Os12g41790.1	-1.025345638	0.001051354	Down
A106919	LOC_Os02g07110.1	-1.025352514	4.80E-005	Down
A129491	LOC_Os06g39240.1	-1.025719669	2.42E-005	Down
A151762	LOC_Os12g32200.1	-1.026359703	1.94E-005	Down
A130178	LOC_Os06g46400.1	-1.026853674	8.54E-005	Down
A130564	LOC_Os06g50460.1	-1.027175336	0.001012799	Down
A112029	LOC_Os03g06370.1	-1.027208606	0.000187243	Down
**A107343**	**LOC_Os02g12400.1**	-1.028108035	**0.000186573**	**Down**
A134173	LOC_Os07g42250.1	-1.028669395	2.18E-005	Down
A104969	LOC_Os01g59680.1	-1.029583522	0.005313698	Down
A119563	LOC_Os04g33360.1	-1.03012151	0.054360461	Down
A140477	LOC_Os09g21740.1	-1.03115212	2.56E-005	Down
A119538	LOC_Os04g33110.1	-1.031364895	2.71E-005	Down
A106465	LOC_Os02g02570.1	-1.031518041	6.08E-005	Down
A120859	LOC_Os04g48220.1	-1.031562342	0.001164284	Down
A152503	LOC_Os12g40160.1	-1.03166395	0.010880258	Down
A145194	LOC_Os10g42620.1	-1.03236933	0.000214668	Down
A150153	LOC_Os12g11700.1	-1.032869718	0.001904736	Down
A136143	LOC_Os08g15430.1	-1.033418042	0.008506832	Down
A123174	LOC_Os05g15510.1	-1.033441491	5.93E-005	Down
A122982	LOC_Os05g13430.1	-1.03427504	4.10E-005	Down
A108059	LOC_Os02g20060.1	-1.034500221	0.014012055	Down
A107027	LOC_Os02g08170.1	-1.034737928	1.45E-005	Down
A130830	LOC_Os07g02750.1	-1.034885977	1.86E-005	Down
A109711	LOC_Os02g39580.1	-1.035101551	3.74E-005	Down
A104878	LOC_Os01g58680.1	-1.03591302	0.000022429	Down

A102606	LOC_Os01g32770.1	-1.036405877	0.00116802	Down
A115471	LOC_Os03g48030.1	-1.038497876	4.91E-005	Down
A150660	LOC_Os12g17600.1	-1.03900627	0.000357126	Down
A100656	LOC_Os01g08050.1	-1.03949959	8.60E-005	Down
A148992	LOC_Os11g45960.1	-1.039767678	0.016693465	Down
A139949	LOC_Os09g15520.1	-1.039850817	2.47E-005	Down
A137077	LOC_Os08g26880.1	-1.040935448	0.030502254	Down
A152635	LOC_Os12g41590.1	-1.041100528	0.043802835	Down
A109959	LOC_Os02g42640.1	-1.042012716	3.34E-005	Down
**A107830**	**LOC_Os02g17534.1**	-1.042016437	**0.000526922**	**Down**
A111046	LOC_Os02g53790.1	-1.042197743	0.000753776	Down
A138660	LOC_Os08g43900.1	-1.042279328	0.007219531	Down
A135738	LOC_Os08g10580.1	-1.043240156	6.95E-005	Down
A119370	LOC_Os04g31250.1	-1.043313373	0.002017969	Down
A102167	LOC_Os01g26000.1	-1.043356867	0.030738169	Down
A145361	LOC_Os11g02369.1	-1.043558588	2.36E-005	Down
A115023	LOC_Os03g43050.1	-1.044218562	0.00320211	Down
A143490	LOC_Os10g21870.1	-1.044316844	0.052142633	Down
A150975	LOC_Os12g22489.1	-1.04441033	1.52E-005	Down
A149268	LOC_Os12g02080.1	-1.044804756	7.15E-005	Down
A105859	LOC_Os01g69890.1	-1.044833059	2.08E-005	Down
A138593	LOC_Os08g43180.1	-1.045067589	0.000395661	Down
A130846	LOC_Os07g02920.1	-1.045663728	0.000160298	Down
A141023	LOC_Os09g28170.1	-1.045953608	5.26E-005	Down
A133701	LOC_Os07g37156.1	-1.04627839	2.07E-005	Down
A125144	LOC_Os05g39930.1	-1.046279712	3.36E-005	Down
A119780	LOC_Os04g35690.1	-1.046783991	0.000246929	Down
A123138	LOC_Os05g15100.1	-1.048117205	0.003153252	Down
A124004	LOC_Os05g26770.1	-1.048185137	2.47E-005	Down
A117440	LOC_Os04g07350.1	-1.048321604	0.006488704	Down
A135452	LOC_Os08g07480.1	-1.0485163	0.033486121	Down
A101101	LOC_Os01g12920.1	-1.049211394	5.41E-005	Down
A117621	LOC_Os04g09910.1	-1.049280297	0.000119679	Down
**A130351**	**LOC_Os06g48180.1**	-1.049590028	**0.000623481**	**Down**
A119442	LOC_Os04g32080.1	-1.049658645	2.37E-005	Down
**A142546**	**LOC_Os10g07578.1**	-1.049951585	**0.041569884**	**Down**
A111931	LOC_Os03g05350.1	-1.049973485	0.00236232	Down
A102119	LOC_Os01g25386.1	-1.050175624	2.13E-005	Down
A136806	LOC_Os08g23760.1	-1.050364255	0.000530691	Down
A119889	LOC_Os04g37760.1	-1.050369492	0.000106793	Down
A131612	LOC_Os07g11380.1	-1.0504374	1.82E-005	Down
A139966	LOC_Os09g15680.1	-1.050929331	0.000197561	Down
A129158	LOC_Os06g35540.1	-1.052901459	4.10E-005	Down
A110748	LOC_Os02g50690.1	-1.053386955	0.00013421	Down
A145363	LOC_Os11g02389.1	-1.053835051	3.18E-005	Down
A149292	LOC_Os12g02320.1	-1.054238073	2.90E-005	Down
A121441	LOC_Os04g54630.1	-1.054388378	7.90E-005	Down
A141518	LOC_Os09g34847.1	-1.054515267	0.001134144	Down

A107938	LOC_Os02g18650.1	-1.054676638	3.35E-005	Down
A114856	LOC_Os03g41030.1	-1.055740441	2.77E-005	Down
A109595	LOC_Os02g38386.1	-1.057007219	8.71E-005	Down
A150542	LOC_Os12g16230.1	-1.058132078	7.10E-005	Down
A104625	LOC_Os01g56050.1	-1.058444669	6.95E-005	Down
A146061	LOC_Os11g09660.1	-1.058759388	0.058964735	Down
A136414	LOC_Os08g18870.1	-1.059037274	0.003492738	Down
**A107599**	**LOC_Os02g15070.1**	**-1.059158108**	**7.33E-005**	**Down**
A110635	LOC_Os02g49550.1	-1.059211455	0.000153359	Down
A108351	LOC_Os02g24240.1	-1.059547155	0.002314713	Down
A104718	LOC_Os01g57020.1	-1.059838405	0.000519029	Down
A139315	LOC_Os09g07990.1	-1.060034516	0.042468641	Down
A144364	LOC_Os10g33200.1	-1.060295085	9.36E-005	Down
A135100	LOC_Os08g03600.1	-1.060327148	0.00319957	Down
A118711	LOC_Os04g23480.1	-1.06050399	0.000287346	Down
A106701	LOC_Os02g04945.1	-1.061105402	0.001794699	Down
A113396	LOC_Os03g21380.1	-1.061178179	5.17E-005	Down
A100018	LOC_Os01g01180.1	-1.06218213	3.25E-005	Down
A101026	LOC_Os01g12150.1	-1.063343038	1.92E-005	Down
A109606	LOC_Os02g38494.1	-1.064074425	0.000020538	Down
A116791	LOC_Os03g62910.1	-1.0647187	0.000131274	Down
A123395	LOC_Os05g18470.1	-1.065252651	1.80E-005	Down
A103775	LOC_Os01g46900.1	-1.066878273	0.022805983	Down
A120742	LOC_Os04g46960.1	-1.066880656	0.000018284	Down
A146360	LOC_Os11g13520.1	-1.066996454	0.005358697	Down
A121100	LOC_Os04g51160.1	-1.067005654	7.31E-005	Down
A114433	LOC_Os03g35650.1	-1.067028338	0.000128927	Down
A149291	LOC_Os12g02310.1	-1.067184726	0.000012486	Down
A122852	LOC_Os05g11580.1	-1.067599381	0.015179852	Down
A151196	LOC_Os12g25260.1	-1.068256764	0.001405769	Down
A116355	LOC_Os03g58180.1	-1.068359895	0.000012289	Down
A144384	LOC_Os10g33400.1	-1.068613829	3.46E-005	Down
A130171	LOC_Os06g46340.1	-1.068743261	2.31E-005	Down
A115175	LOC_Os03g44810.1	-1.068875347	0.000121568	Down
A132729	LOC_Os07g26310.1	-1.069231225	0.001333593	Down
A110807	LOC_Os02g51280.1	-1.069428891	8.40E-005	Down
A138458	LOC_Os08g41750.1	-1.071185683	1.40E-005	Down
A106712	LOC_Os02g05070.1	-1.071224361	4.71E-005	Down
A132316	LOC_Os07g20250.1	-1.071451419	0.000598292	Down
A119588	LOC_Os04g33610.1	-1.071722288	0.006445731	Down
A103391	LOC_Os01g42030.1	-1.073189522	6.25E-005	Down
A100977	LOC_Os01g11620.1	-1.073357973	2.15E-005	Down
A117925	LOC_Os04g13480.1	-1.073484384	0.003609059	Down
A134473	LOC_Os07g45540.1	-1.073522974	0.000661012	Down
A129230	LOC_Os06g36380.1	-1.073810763	0.000101727	Down
A125057	LOC_Os05g39010.1	-1.074050025	0.017637481	Down
A121370	LOC_Os04g53910.1	-1.075285437	0.025723677	Down
A152178	LOC_Os12g36710.1	-1.076024254	0.010450512	Down

**A130615**	**LOC_Os06g51084.1**	**-1.076111872**	**1.58E-005**	**Down**
A107384	LOC_Os02g12810.1	-1.077379914	1.77E-005	Down
A107840	LOC_Os02g17640.1	-1.077602638	0.00010482	Down
A131624	LOC_Os07g11510.1	-1.080249979	0.000116667	Down
A150737	LOC_Os12g18530.1	-1.080345692	0.00386605	Down
A134351	LOC_Os07g44180.1	-1.080522336	0.000017091	Down
A107437	LOC_Os02g13370.1	-1.082050821	1.48E-005	Down
A122795	LOC_Os05g10950.1	-1.082437764	0.000145211	Down
A152689	LOC_Os12g42110.1	-1.085662682	0.049662382	Down
A135532	LOC_Os08g08360.1	-1.086012128	4.04E-005	Down
A124400	LOC_Os05g31620.1	-1.08645187	0.000014075	Down
A116549	LOC_Os03g60350.1	-1.087771584	0.000163029	Down
A101347	LOC_Os01g15640.1	-1.088262801	2.24E-005	Down
A125321	LOC_Os05g41770.1	-1.091044204	0.001968162	Down
A137614	LOC_Os08g32870.1	-1.091868664	3.09E-005	Down
A129844	LOC_Os06g42930.1	-1.092071373	8.26E-005	Down
A101588	LOC_Os01g18800.1	-1.092690983	0.000203242	Down
A100997	LOC_Os01g11830.1	-1.093261661	0.019837643	Down
A148207	LOC_Os11g37270.1	-1.093957647	1.17E-005	Down
A111058	LOC_Os02g53900.1	-1.094674155	2.95E-005	Down
A150062	LOC_Os12g10670.1	-1.095184519	0.040884265	Down
A138461	LOC_Os08g41780.1	-1.098107826	4.36E-005	Down
**A124585**	**LOC_Os05g33570.1**	**-1.098147314**	**7.44E-005**	**Down**
A106005	LOC_Os01g71380.1	-1.098989616	4.23E-005	Down
A151918	LOC_Os12g34010.1	-1.101105708	1.49E-005	Down
A115218	LOC_Os03g45280.1	-1.102291769	2.21E-005	Down
A145062	LOC_Os10g41090.1	-1.104608899	0.054971068	Down
A149284	LOC_Os12g02240.1	-1.106097004	0.001278829	Down
A106792	LOC_Os02g05860.1	-1.10653342	0.001393976	Down
A108926	LOC_Os02g30550.1	-1.107705856	3.34E-005	Down
**A152528**	**LOC_Os12g40419.1**	**-1.108436664**	**4.62E-005**	**Down**
A101767	LOC_Os01g21250.1	-1.108792706	6.10E-005	Down
A103979	LOC_Os01g49070.1	-1.109240127	0.008803853	Down
A151271	LOC_Os12g26100.1	-1.10975063	0.018492098	Down
A112748	LOC_Os03g14440.1	-1.110170852	0.002333666	Down
A106343	LOC_Os02g01280.1	-1.110373454	6.12E-005	Down
A127753	LOC_Os06g17629.1	-1.110953164	0.000108911	Down
A110329	LOC_Os02g46380.1	-1.111389001	6.84E-005	Down
A105228	LOC_Os01g62520.1	-1.11190797	0.00024833	Down
A110166	LOC_Os02g44654.1	-1.113673522	0.05331081	Down
A110054	LOC_Os02g43620.1	-1.114001799	4.64E-005	Down
A105310	LOC_Os01g63444.1	-1.114050241	0.0018994	Down
A127474	LOC_Os06g14520.1	-1.114151962	0.000013852	Down
A122544	LOC_Os05g07890.1	-1.114578573	2.10E-005	Down
A140682	LOC_Os09g24580.1	-1.11487359	1.94E-005	Down
A128592	LOC_Os06g28760.1	-1.115378082	0.059287618	Down
A113287	LOC_Os03g20210.1	-1.116160968	0.056354081	Down
A105532	LOC_Os01g65830.1	-1.116352139	0.000270735	Down

A136196	LOC_Os08g16010.1	-1.116592222	0.00013794	Down
A127441	LOC_Os06g14170.1	-1.117812895	0.001380101	Down
A117264	LOC_Os04g05060.1	-1.11941688	0.007712871	Down
A126124	LOC_Os05g51130.1	-1.119932852	2.54E-005	Down
A134078	LOC_Os07g41200.1	-1.122410086	0.00011956	Down
A110337	LOC_Os02g46473.1	-1.122564144	1.82E-005	Down
A152236	LOC_Os12g37310.1	-1.123257579	4.30E-005	Down
A149542	LOC_Os12g05050.1	-1.124509481	1.76E-005	Down
A105575	LOC_Os01g66270.1	-1.124822517	0.000132531	Down
A147098	LOC_Os11g24070.1	-1.124971765	9.54E-006	Down
A131670	LOC_Os07g12090.1	-1.125239019	1.58E-005	Down
A130168	LOC_Os06g46284.1	-1.127058025	1.30E-005	Down
A125341	LOC_Os05g41970.1	-1.127379958	7.93E-005	Down
A131606	LOC_Os07g11320.1	-1.128170391	0.00001012	Down
A101654	LOC_Os01g19570.1	-1.128767133	0.001188319	Down
A142996	LOC_Os10g14060.1	-1.129371534	0.019160223	Down
A134739	LOC_Os07g48340.1	-1.129777913	2.78E-005	Down
A105519	LOC_Os01g65700.1	-1.129997136	0.007024313	Down
A138655	LOC_Os08g43850.1	-1.130305779	0.004557898	Down
A103090	LOC_Os01g38720.1	-1.131234961	0.007210517	Down
A131396	LOC_Os07g09140.1	-1.132267455	8.17E-005	Down
**A112562**	**LOC_Os03g12470.1**	-1.133121397	**0.000153217**	**Down**
**A141969**	**LOC_Os09g39920.1**	-1.133376429	**0.000079416**	**Down**
A100810	LOC_Os01g09680.1	-1.133441387	0.001046405	Down
A147676	LOC_Os11g31060.1	-1.134569356	9.41E-006	Down
A105458	LOC_Os01g65030.1	-1.134630762	0.021634241	Down
**A109849**	**LOC_Os02g41500.1**	-1.13659967	**3.19E-005**	**Down**
A119051	LOC_Os04g27610.1	-1.136915817	0.003701753	Down
A115803	LOC_Os03g51760.1	-1.137154851	0.000981814	Down
A131325	LOC_Os07g08380.1	-1.13934221	0.000534053	Down
A149852	LOC_Os12g08350.1	-1.140711732	0.040967745	Down
A119712	LOC_Os04g34970.1	-1.140874399	1.05E-005	Down
A105196	LOC_Os01g62180.1	-1.142257376	0.000009293	Down
A137319	LOC_Os08g29600.1	-1.142631801	8.68E-006	Down
A134120	LOC_Os07g41660.1	-1.143531674	0.000216481	Down
A141214	LOC_Os09g30240.1	-1.146107997	1.76E-005	Down
**A127202**	**LOC_Os06g11650.1**	-1.146226239	**0.000708066**	**Down**
A118961	LOC_Os04g26520.1	-1.146297593	0.000205437	Down
A152577	LOC_Os12g40940.1	-1.146694993	8.12E-005	Down
A146816	LOC_Os11g19180.1	-1.146860245	0.000340616	Down
A114050	LOC_Os03g29880.1	-1.147288377	0.021459411	Down
A105701	LOC_Os01g67660.1	-1.148338017	0.019346843	Down
A140436	LOC_Os09g21270.1	-1.149145828	8.37E-005	Down
A105260	LOC_Os01g62850.1	-1.14919787	2.53E-005	Down
A120773	LOC_Os04g47310.1	-1.149330456	2.59E-005	Down
A125056	LOC_Os05g39000.1	-1.15031816	3.20E-005	Down
A145850	LOC_Os11g07350.1	-1.150593767	0.001943523	Down
A143793	LOC_Os10g25990.1	-1.152716713	3.18E-005	Down

A130838	LOC_Os07g02840.1	-1.153545176	0.051302639	Down
A109612	LOC_Os02g38580.1	-1.153796778	1.19E-005	Down
A123996	LOC_Os05g26690.1	-1.154363939	8.68E-006	Down
A137312	LOC_Os08g29530.1	-1.154451856	8.93E-005	Down
A144958	LOC_Os10g39930.1	-1.155072975	0.000025791	Down
A122449	LOC_Os05g06720.1	-1.155299277	0.000330494	Down
A140709	LOC_Os09g24870.1	-1.156042448	0.001622655	Down
A136274	LOC_Os08g16800.1	-1.157645831	7.47E-005	Down
A139964	LOC_Os09g15660.1	-1.158328298	8.57E-006	Down
A141110	LOC_Os09g29090.1	-1.158611674	0.000726987	Down
A144082	LOC_Os10g29670.1	-1.158967009	1.32E-005	Down
A137797	LOC_Os08g34800.1	-1.161258269	0.00010458	Down
A100312	LOC_Os01g04360.1	-1.162047239	0.00000755	Down
A114815	LOC_Os03g40440.1	-1.163940739	2.13E-005	Down
A130513	LOC_Os06g49930.1	-1.164038312	0.019532422	Down
**A112002**	**LOC_Os03g06050.1**	**-1.165123886**	**2.07E-005**	**Down**
**A107798**	**LOC_Os02g17190.1**	**-1.167705349**	**1.16E-005**	**Down**
A108948	LOC_Os02g30790.1	-1.168769909	2.35E-005	Down
A101169	LOC_Os01g13670.1	-1.169623078	0.010576162	Down
A145660	LOC_Os11g05410.1	-1.169972926	8.88E-006	Down
A120403	LOC_Os04g43300.1	-1.170691353	1.57E-005	Down
A119581	LOC_Os04g33540.1	-1.171660448	0.000329622	Down
A138764	LOC_Os08g45000.1	-1.172877556	0.001700109	Down
A124583	LOC_Os05g33554.1	-1.173639604	1.44E-005	Down
A122754	LOC_Os05g10550.1	-1.173830362	0.016461189	Down
A131623	LOC_Os07g11500.1	-1.174143816	0.015931803	Down
A110588	LOC_Os02g49070.1	-1.174583002	5.59E-005	Down
A111106	LOC_Os02g54430.1	-1.175173246	0.000572327	Down
**A104395**	**LOC_Os01g53640.1**	**-1.176256407**	**0.000190774**	**Down**
A142388	LOC_Os10g05690.1	-1.177920034	1.71E-005	Down
**A109163**	**LOC_Os02g33580.1**	**-1.179592103**	**0.000117284**	**Down**
A137527	LOC_Os08g31950.1	-1.181979824	1.13E-005	Down
A115292	LOC_Os03g46150.1	-1.183640972	0.031944171	Down
A100314	LOC_Os01g04380.1	-1.184578675	9.08E-006	Down
A144407	LOC_Os10g33660.1	-1.185261164	8.60E-006	Down
**A148972**	**LOC_Os11g45740.1**	**-1.187104077**	**0.003229546**	**Down**
A111929	LOC_Os03g05334.1	-1.18737585	0.003933708	Down
A145789	LOC_Os11g06730.1	-1.188096643	0.004275098	Down
A136518	LOC_Os08g19970.1	-1.188937388	9.94E-005	Down
A138595	LOC_Os08g43200.1	-1.189072354	1.13E-005	Down
A145930	LOC_Os11g08210.1	-1.189529314	2.09E-005	Down
**A112509**	**LOC_Os03g11900.1**	**-1.19058619**	**0.000111726**	**Down**
A133771	LOC_Os07g37920.1	-1.191016944	0.000442908	Down
A107087	LOC_Os02g09260.1	-1.191469634	1.98E-005	Down
A110616	LOC_Os02g49350.1	-1.19176867	4.48E-005	Down
**A136973**	**LOC_Os08g25734.1**	**-1.192296813**	**1.82E-005**	**Down**
A142789	LOC_Os10g10664.1	-1.19266572	2.35E-005	Down
A151970	LOC_Os12g34419.1	-1.193772935	0.001019682	Down

**A122850**	**LOC_Os05g11560.1**	**-1.193959569**	**0.000825748**	**Down**
A111940	LOC_Os03g05440.1	-1.194505736	2.44E-005	Down
A145337	LOC_Os11g02130.1	-1.195462357	6.81E-005	Down
A102996	LOC_Os01g37630.1	-1.197784332	5.73E-005	Down
A134376	LOC_Os07g44440.1	-1.197784978	2.94E-005	Down
A116638	LOC_Os03g61330.1	-1.201240554	1.07E-005	Down
**A128162**	**LOC_Os06g22730.1**	**-1.202595428**	**0.056522604**	**Down**
A124703	LOC_Os05g34800.1	-1.203229585	0.001621663	Down
A121521	LOC_Os04g55510.1	-1.203712972	9.14E-006	Down
A140845	LOC_Os09g26310.1	-1.204025064	2.42E-005	Down
**A141602**	**LOC_Os09g36180.1**	**-1.204231393**	**2.70E-005**	**Down**
A133982	LOC_Os07g40130.1	-1.20476308	0.000103461	Down
A139971	LOC_Os09g15740.1	-1.204824943	0.041226598	Down
A139549	LOC_Os09g10880.1	-1.204843406	0.002063625	Down
A107121	LOC_Os02g09630.1	-1.204863476	0.000113491	Down
A132876	LOC_Os07g28060.1	-1.206432516	0.001623085	Down
A134711	LOC_Os07g48040.1	-1.20835811	0.017499075	Down
A128832	LOC_Os06g31562.1	-1.209172628	4.40E-005	Down
A139093	LOC_Os09g04580.1	-1.211203243	0.000124461	Down
A125706	LOC_Os05g46350.1	-1.211779915	0.000167205	Down
A101090	LOC_Os01g12810.1	-1.214015502	8.93E-006	Down
A114020	LOC_Os03g29410.1	-1.215092175	0.00063219	Down
A117433	LOC_Os04g07230.1	-1.215287437	0.000144088	Down
A102724	LOC_Os01g34200.1	-1.215582734	1.27E-005	Down
A100924	LOC_Os01g10950.1	-1.216151922	0.001706926	Down
A138178	LOC_Os08g38830.1	-1.216441952	0.000138954	Down
**A142468**	**LOC_Os10g06740.1**	**-1.217314597**	**0.004291986**	**Down**
A110464	LOC_Os02g47810.1	-1.21807264	6.62E-006	Down
A120628	LOC_Os04g45780.1	-1.218816959	0.000850821	Down
A105975	LOC_Os01g71090.1	-1.219604522	1.35E-005	Down
A113102	LOC_Os03g18230.1	-1.221938537	0.000115074	Down
A129414	LOC_Os06g38440.1	-1.222876511	0.00174511	Down
A110533	LOC_Os02g48480.1	-1.223396077	9.50E-006	Down
**A141869**	**LOC_Os09g38910.1**	**-1.224948007**	**4.21E-005**	**Down**
A112663	LOC_Os03g13540.1	-1.225629119	1.48E-005	Down
A140008	LOC_Os09g16120.1	-1.226041346	0.011127654	Down
A117069	LOC_Os04g02630.1	-1.226231698	0.003601307	Down
A104895	LOC_Os01g58850.1	-1.227593633	1.12E-005	Down
A141834	LOC_Os09g38570.1	-1.229984293	2.20E-005	Down
A109113	LOC_Os02g33050.1	-1.231505695	4.00E-005	Down
A129370	LOC_Os06g37890.1	-1.232990347	0.043277831	Down
A108950	LOC_Os02g30810.1	-1.234541656	1.15E-005	Down
A116022	LOC_Os03g54240.1	-1.23481875	0.000044269	Down
A109306	LOC_Os02g35180.1	-1.236816196	0.00200025	Down
A110956	LOC_Os02g52850.1	-1.238277528	0.000139078	Down
A116081	LOC_Os03g55350.1	-1.238848101	9.55E-006	Down
A101048	LOC_Os01g12381.1	-1.239972475	2.08E-005	Down
A136754	LOC_Os08g23210.1	-1.240778474	0.000482083	Down

**A104066**	**LOC_Os01g50030.1**	**-1.241096081**	**8.35E-006**	**Down**
A142549	LOC_Os10g07604.1	-1.242119321	6.58E-005	Down
A149061	LOC_Os11g47229.1	-1.24214248	0.033021454	Down
A106568	LOC_Os02g03640.1	-1.242560327	0.003244512	Down
A109214	LOC_Os02g34190.1	-1.242665737	0.000143105	Down
A122823	LOC_Os05g11240.1	-1.243192985	0.01435584	Down
A128139	LOC_Os06g22490.1	-1.244095894	0.014014518	Down
A110022	LOC_Os02g43314.1	-1.245096553	0.000122971	Down
A141605	LOC_Os09g36210.1	-1.24513	0.000008053	Down
A120578	LOC_Os04g45200.1	-1.246791207	2.42E-005	Down
**A104067**	**LOC_Os01g50032.1**	**-1.246997096**	**5.79E-006**	**Down**
A127630	LOC_Os06g16250.1	-1.247077043	0.000174813	Down
A102379	LOC_Os01g28989.2	-1.247398974	0.000016584	Down
A102918	LOC_Os01g36720.1	-1.248903344	0.000236092	Down
A130824	LOC_Os07g02690.1	-1.250235129	0.000016031	Down
A123927	LOC_Os05g25850.1	-1.252286707	0.000013126	Down
A150580	LOC_Os12g16620.1	-1.253741145	0.046089563	Down
A133728	LOC_Os07g37454.1	-1.253826798	0.000538213	Down
A128837	LOC_Os06g31700.1	-1.254025516	0.000310388	Down
A114481	LOC_Os03g36460.1	-1.254658124	0.000160887	Down
A141722	LOC_Os09g37390.1	-1.255122013	0.00006449	Down
A110699	LOC_Os02g50220.1	-1.256757067	0.002000283	Down
A120777	LOC_Os04g47360.1	-1.25730742	0.018552225	Down
A150886	LOC_Os12g21490.1	-1.257520029	0.000306317	Down
A102523	LOC_Os01g31810.2	-1.259028764	4.58E-005	Down
A112268	LOC_Os03g08860.1	-1.259611554	1.22E-005	Down
A150606	LOC_Os12g16970.1	-1.259724868	0.000532472	Down
A139954	LOC_Os09g15570.1	-1.259766619	0.00128282	Down
A144716	LOC_Os10g37079.1	-1.259811183	0.000180917	Down
A104877	LOC_Os01g58670.1	-1.259911874	7.26E-006	Down
A109155	LOC_Os02g33500.1	-1.261333103	6.15E-005	Down
A110291	LOC_Os02g45974.1	-1.26192644	1.62E-005	Down
A103355	LOC_Os01g41660.1	-1.263239046	7.03E-006	Down
A137877	LOC_Os08g35580.1	-1.264586835	0.017399973	Down
**A149021**	**LOC_Os11g46806.1**	**-1.265942003**	**0.046890218**	**Down**
A100747	LOC_Os01g09010.1	-1.266625606	1.16E-005	Down
A138894	LOC_Os09g02270.1	-1.268386329	0.000758108	Down
A135736	LOC_Os08g10560.1	-1.270255516	0.000091472	Down
A108662	LOC_Os02g27590.1	-1.271052002	1.27E-005	Down
A150386	LOC_Os12g14220.1	-1.272352678	2.52E-005	Down
A107997	LOC_Os02g19310.1	-1.272902126	0.000228101	Down
A105356	LOC_Os01g63970.1	-1.274152173	0.000041871	Down
A126515	LOC_Os06g04430.1	-1.274992778	3.12E-005	Down
**A144208**	**LOC_Os10g31200.1**	**-1.276234887**	**1.21E-005**	**Down**
A102763	LOC_Os01g34660.1	-1.276396384	1.07E-005	Down
A130867	LOC_Os07g03120.1	-1.277290157	0.004958524	Down
A110632	LOC_Os02g49520.1	-1.278610317	0.000107116	Down
A127104	LOC_Os06g10610.1	-1.279203884	0.000533082	Down

**A114623**	**LOC_Os03g38210.1**	**-1.280526409**	**0.011603471**	**Down**
A100099	LOC_Os01g02050.1	**-1.282947459**	0.002323306	Down
A135557	LOC_Os08g08650.1	**-1.283004011**	2.32E-005	Down
A120518	LOC_Os04g44570.1	**-1.283436829**	6.05E-006	Down
A117061	LOC_Os04g02530.1	**-1.283947168**	0.000104715	Down
A138320	LOC_Os08g40330.1	**-1.28395772**	0.030186225	Down
**A106446**	**LOC_Os02g02370.1**	**-1.285440891**	**0.010090093**	**Down**
A118486	LOC_Os04g20749.1	**-1.288793543**	1.44E-005	Down
A104842	LOC_Os01g58320.1	**-1.292824875**	0.000819897	Down
A105515	LOC_Os01g65670.1	**-1.29305595**	0.000053652	Down
A141651	LOC_Os09g36690.1	**-1.293447367**	7.94E-005	Down
A133508	LOC_Os07g35050.1	**-1.296527774**	0.000253255	Down
A149556	LOC_Os12g05200.1	**-1.296638582**	0.000884647	Down
A124298	LOC_Os05g30540.1	**-1.297312956**	1.03E-005	Down
A112390	LOC_Os03g10640.1	**-1.299053481**	0.000137013	Down
A122260	LOC_Os05g04690.1	**-1.299367036**	0.002964424	Down
A104332	LOC_Os01g52900.1	**-1.302121097**	7.11E-006	Down
A110868	LOC_Os02g51890.1	**-1.30233629**	6.45E-006	Down
A116577	LOC_Os03g60650.1	**-1.302717038**	0.000102276	Down
A132847	LOC_Os07g27710.1	**-1.305516417**	0.000240806	Down
A101045	LOC_Os01g12340.1	**-1.305851185**	0.005579043	Down
A110338	LOC_Os02g46480.1	**-1.306020289**	5.01E-006	Down
A116006	LOC_Os03g54050.1	**-1.30630341**	4.32E-006	Down
A150308	LOC_Os12g13410.1	**-1.306976447**	1.08E-005	Down
A143302	LOC_Os10g19898.1	**-1.30865232**	0.021191966	Down
**A107763**	**LOC_Os02g16830.1**	**-1.31084372**	**8.76E-006**	**Down**
A100819	LOC_Os01g09770.1	**-1.31362915**	7.46E-006	Down
A113124	LOC_Os03g18470.1	**-1.31561028**	0.000204347	Down
A149106	LOC_Os11g47520.1	**-1.316323456**	0.000164553	Down
A125169	LOC_Os05g40180.1	**-1.317647107**	0.011777043	Down
**A120764**	**LOC_Os04g47210.1**	**-1.318938454**	**6.19E-005**	**Down**
A126147	LOC_Os05g51390.1	**-1.31907301**	0.000155902	Down
A130491	LOC_Os06g49700.1	**-1.319996932**	0.000003845	Down
A135723	LOC_Os08g10430.1	**-1.320041435**	0.000024808	Down
A138587	LOC_Os08g43120.1	**-1.320237925**	0.002277631	Down
A117900	LOC_Os04g13190.1	**-1.320328812**	3.06E-005	Down
A136902	LOC_Os08g24910.1	**-1.320892815**	0.036756272	Down
A133249	LOC_Os07g32220.1	**-1.321404293**	0.002486877	Down
A131731	LOC_Os07g12700.1	**-1.327529694**	6.12E-006	Down
A108924	LOC_Os02g30530.1	**-1.327878939**	0.002952067	Down
A109596	LOC_Os02g38392.1	**-1.328911988**	8.12E-005	Down
A121308	LOC_Os04g53250.1	**-1.329714389**	0.00010727	Down
A149117	LOC_Os11g47630.1	**-1.331970957**	0.000231024	Down
A141868	LOC_Os09g38900.1	**-1.333002195**	3.39E-005	Down
**A128508**	**LOC_Os06g27770.1**	**-1.333616475**	**4.56E-006**	**Down**
A134281	LOC_Os07g43400.1	**-1.333986167**	3.60E-006	Down
A113309	LOC_Os03g20440.1	**-1.335078969**	5.08E-006	Down
A115485	LOC_Os03g48180.1	**-1.336937841**	1.01E-005	Down

A152323	LOC_Os12g38260.1	-1.337886849	0.053902026	Down
A109654	LOC_Os02g39030.1	-1.338071591	6.79E-006	Down
**A109851**	**LOC_Os02g41520.1**	-1.33999128	**1.33E-005**	**Down**
A149377	LOC_Os12g03220.1	-1.340855684	0.02489009	Down
A132497	LOC_Os07g23410.1	-1.340951352	5.44E-006	Down
A134135	LOC_Os07g41820.1	-1.341017304	4.19E-006	Down
A120572	LOC_Os04g45130.1	-1.343634077	3.56E-006	Down
A136529	LOC_Os08g20090.2	-1.345663069	5.62E-006	Down
A116557	LOC_Os03g60440.1	-1.347277156	2.20E-005	Down
A140350	LOC_Os09g20380.1	-1.351654441	1.24E-005	Down
A135058	LOC_Os08g03110.1	-1.352253906	4.71E-006	Down
A127459	LOC_Os06g14370.1	-1.353613966	3.47E-006	Down
A106161	LOC_Os01g73120.1	-1.353664267	0.000004098	Down
A114014	LOC_Os03g29340.1	-1.357001274	1.76E-005	Down
A118258	LOC_Os04g17880.1	-1.360393044	0.000300852	Down
A103496	LOC_Os01g43200.1	-1.360727612	0.016507551	Down
A138509	LOC_Os08g42310.1	-1.36141811	6.79E-005	Down
**A110405**	**LOC_Os02g47190.1**	-1.362213114	**0.001937947**	**Down**
A134935	LOC_Os08g01810.1	-1.364663022	0.000008103	Down
A130570	LOC_Os06g50520.1	-1.366227386	0.000228841	Down
A113201	LOC_Os03g19275.1	-1.366849901	2.54E-005	Down
A109965	LOC_Os02g42710.1	-1.368052012	0.000079606	Down
A111759	LOC_Os03g03510.1	-1.368114212	8.43E-006	Down
**A121267**	**LOC_Os04g52840.1**	-1.368245275	**3.51E-006**	**Down**
A146152	LOC_Os11g10600.1	-1.368826425	0.058061386	Down
A111653	LOC_Os03g02300.1	-1.370468738	8.68E-005	Down
A134185	LOC_Os07g42390.1	-1.372588338	0.000008049	Down
A105711	LOC_Os01g67760.1	-1.373082473	0.000333036	Down
A130844	LOC_Os07g02900.1	-1.37444571	3.94E-005	Down
A145043	LOC_Os10g40830.1	-1.375069629	5.32E-005	Down
A146214	LOC_Os11g11320.1	-1.376311442	0.00011054	Down
A110979	LOC_Os02g53070.1	-1.376669961	7.46E-006	Down
A148770	LOC_Os11g43470.1	-1.37729768	0.000125427	Down
A121046	LOC_Os04g50204.1	-1.377583562	7.97E-006	Down
**A138219**	**LOC_Os08g39270.1**	-1.378223139	**3.94E-005**	**Down**
A104908	LOC_Os01g59009.1	-1.380353522	0.010099053	Down
**A130919**	**LOC_Os07g03820.1**	-1.380805378	**3.98E-005**	**Down**
A117073	LOC_Os04g02670.1	-1.381158252	5.73E-006	Down
A139004	LOC_Os09g03540.1	-1.384505038	0.012936444	Down
A109480	LOC_Os02g37150.1	-1.38492494	1.47E-005	Down
A110921	LOC_Os02g52450.1	-1.385382622	2.39E-005	Down
A103611	LOC_Os01g45000.1	-1.385686623	0.000286795	Down
A129922	LOC_Os06g43770.1	-1.385787664	7.39E-006	Down
A129057	LOC_Os06g34450.1	-1.387979392	2.63E-005	Down
A102036	LOC_Os01g24500.1	-1.388504064	0.00269723	Down
A131657	LOC_Os07g11920.1	-1.389398314	2.80E-006	Down
A138180	LOC_Os08g38850.1	-1.39116037	9.19E-006	Down
A108664	LOC_Os02g27594.1	-1.392036459	5.05E-005	Down

A101018	LOC_Os01g12060.1	-1.392150661	0.000106372	Down
A139995	LOC_Os09g15980.1	-1.395697419	0.008039669	Down
A110321	LOC_Os02g46290.1	-1.397529062	7.30E-006	Down
A120495	LOC_Os04g44320.1	-1.398028325	8.49E-006	Down
A117403	LOC_Os04g06840.1	-1.398199797	0.004211827	Down
A101975	LOC_Os01g23810.1	-1.398222562	0.008977794	Down
A102548	LOC_Os01g32070.1	-1.400279626	0.014368738	Down
**A133705**	**LOC_Os07g37210.1**	**-1.403012095**	**3.11E-006**	**Down**
A111971	LOC_Os03g05750.1	-1.407729848	7.57E-005	Down
A107929	LOC_Os02g18580.1	-1.40986928	0.000399746	Down
A138233	LOC_Os08g39410.1	-1.412759731	5.35E-005	Down
**A138825**	**LOC_Os09g01430.1**	**-1.41305764**	**0.019929032**	**Down**
**A110934**	**LOC_Os02g52590.1**	**-1.413181296**	**4.18E-006**	**Down**
A151575	LOC_Os12g29970.1	-1.413826002	5.88E-006	Down
A127553	LOC_Os06g15430.1	-1.421430686	2.71E-005	Down
A131811	LOC_Os07g13634.1	-1.422155294	2.71E-006	Down
A130374	LOC_Os06g48490.1	-1.424998031	0.00295188	Down
A109302	LOC_Os02g35144.1	-1.425325196	0.000249591	Down
A123451	LOC_Os05g19160.1	-1.426985979	6.51E-005	Down
A108896	LOC_Os02g30240.1	-1.427860172	4.10E-006	Down
A148019	LOC_Os11g35210.1	-1.428138359	0.00069381	Down
A131615	LOC_Os07g11410.1	-1.428875635	2.04E-006	Down
A127405	LOC_Os06g13760.1	-1.432587829	0.000135639	Down
A102247	LOC_Os01g27449.1	-1.436437014	4.93E-006	Down
A107691	LOC_Os02g16040.1	-1.43729497	9.46E-006	Down
A109173	LOC_Os02g33680.1	-1.439007093	0.000143852	Down
A132323	LOC_Os07g20340.1	-1.440016957	2.77E-006	Down
A110750	LOC_Os02g50710.1	-1.442037329	2.20E-006	Down
A113531	LOC_Os03g22820.1	-1.451248225	0.000024706	Down
A123218	LOC_Os05g16054.2	-1.451819588	0.015075359	Down
A112145	LOC_Os03g07590.1	-1.452760702	8.50E-006	Down
A136419	LOC_Os08g18910.1	-1.456003044	4.31E-005	Down
**A136751**	**LOC_Os08g23180.1**	**-1.458401989**	**7.86E-006**	**Down**
A146139	LOC_Os11g10470.1	-1.458618457	2.85E-005	Down
A136349	LOC_Os08g17630.1	-1.459659096	0.001435878	Down
A135816	LOC_Os08g11450.1	-1.460138312	5.11E-005	Down
A140279	LOC_Os09g19650.1	-1.463700938	1.43E-005	Down
A108879	LOC_Os02g30070.1	-1.466772181	2.22E-005	Down
A122967	LOC_Os05g12800.1	-1.467936594	4.64E-005	Down
A114964	LOC_Os03g42380.1	-1.469411662	4.88E-005	Down
**A120548**	**LOC_Os04g44900.1**	**-1.471291743**	**4.28E-006**	**Down**
A149137	LOC_Os11g47840.1	-1.474641784	3.89E-006	Down
A104979	LOC_Os01g59780.1	-1.474836996	8.07E-006	Down
A125305	LOC_Os05g41610.1	-1.475658233	7.62E-006	Down
**A126963**	**LOC_Os06g09090.1**	**-1.477996665**	**1.25E-005**	**Down**
A109653	LOC_Os02g39020.1	-1.478249858	3.87E-005	Down
A127351	LOC_Os06g13190.1	-1.478693225	2.17E-005	Down
A142020	LOC_Os10g01430.1	-1.480268527	3.05E-006	Down

A102490	LOC_Os01g31370.1	-1.480419969	3.65E-006	Down
A110422	LOC_Os02g47370.1	-1.482436227	1.04E-005	Down
A106746	LOC_Os02g05400.1	-1.484622069	0.000003902	Down
A134163	LOC_Os07g42130.1	-1.492428071	5.53E-005	Down
A128099	LOC_Os06g22060.1	-1.492999551	1.84E-005	Down
A105289	LOC_Os01g63210.1	-1.49342094	0.000002089	Down
**A133204**	**LOC_Os07g31690.1**	**-1.495722321**	**0.000077716**	**Down**
A139523	LOC_Os09g10620.1	-1.497819188	1.72E-006	Down
A135551	LOC_Os08g08592.1	-1.50530907	0.002315083	Down
**A129964**	**LOC_Os06g44180.1**	**-1.510419774**	**2.78E-005**	**Down**
A133767	LOC_Os07g37880.1	-1.510844422	1.77E-005	Down
A110125	LOC_Os02g44235.1	-1.511609629	0.000217863	Down
A132301	LOC_Os07g20110.1	-1.515840744	0.000010285	Down
A152330	LOC_Os12g38330.1	-1.51627783	1.95E-006	Down
**A100120**	**LOC_Os01g02290.1**	**-1.517390461**	**0.001655593**	**Down**
A103523	LOC_Os01g43480.1	-1.524028338	4.00E-006	Down
A109818	LOC_Os02g40680.1	-1.526472173	2.58E-006	Down
A103485	LOC_Os01g43080.1	-1.528429297	1.70E-006	Down
A137249	LOC_Os08g28810.1	-1.53099695	0.000149042	Down
A128098	LOC_Os06g22050.1	-1.531857328	3.23E-005	Down
**A138629**	**LOC_Os08g43550.1**	**-1.534207642**	**1.98E-006**	**Down**
A132395	LOC_Os07g22224.1	-1.545117352	2.95E-006	Down
A125957	LOC_Os05g49430.1	-1.545719798	3.36E-005	Down
A133993	LOC_Os07g40240.1	-1.548828454	1.62E-006	Down
A106099	LOC_Os01g72420.1	-1.550087447	5.41E-006	Down
A136713	LOC_Os08g22604.1	-1.55009614	5.66E-006	Down
**A108959**	**LOC_Os02g30910.1**	**-1.55155035**	**4.41E-006**	**Down**
A135150	LOC_Os08g04090.1	-1.551864971	2.01E-006	Down
A130839	LOC_Os07g02850.1	-1.552206098	4.48E-006	Down
**A113014**	**LOC_Os03g17300.1**	**-1.55258241**	**1.45E-005**	**Down**
A115757	LOC_Os03g51260.1	-1.554477742	1.35E-005	Down
A102204	LOC_Os01g26970.5	-1.55659568	1.88E-005	Down
A119587	LOC_Os04g33600.1	-1.560256343	0.000105188	Down
A110771	LOC_Os02g50900.1	-1.565941801	0.001952063	Down
A112351	LOC_Os03g10240.1	-1.566688169	1.37E-006	Down
A102898	LOC_Os01g36510.1	-1.567723747	0.000267278	Down
A115490	LOC_Os03g48230.1	-1.569607732	0.000100126	Down
A128423	LOC_Os06g25840.1	-1.578982455	0.000672133	Down
**A109459**	**LOC_Os02g36924.1**	**-1.580990911**	**2.11E-006**	**Down**
A121904	LOC_Os04g59540.1	-1.583462649	2.60E-006	Down
A106134	LOC_Os01g72810.1	-1.584582187	2.01E-006	Down
A110581	LOC_Os02g48990.1	-1.585370764	1.01E-005	Down
A124523	LOC_Os05g32950.1	-1.587098583	0.000398114	Down
A126106	LOC_Os05g50940.1	-1.587173564	1.59E-006	Down
A147136	LOC_Os11g24570.1	-1.590975918	6.10E-005	Down
**A135611**	**LOC_Os08g09230.1**	**-1.593146289**	**1.50E-006**	**Down**
A109611	LOC_Os02g38574.1	-1.597898905	1.86E-006	Down
A149983	LOC_Os12g09800.1	-1.598884793	0.000012292	Down

A135817	LOC_Os08g11470.1	-1.59888582	3.37E-005	Down
**A110877**	**LOC_Os02g52010.1**	-1.599700544	**0.00001019**	**Down**
A101393	LOC_Os01g16140.1	-1.603891645	0.000001903	Down
A146935	LOC_Os11g20580.1	-1.604145259	0.000495033	Down
A108931	LOC_Os02g30610.1	-1.612078461	1.26E-006	Down
A122022	LOC_Os05g02060.1	-1.617865143	1.24E-006	Down
A144576	LOC_Os10g35424.1	-1.625257953	0.000006092	Down
A122905	LOC_Os05g12140.1	-1.62633141	0.000001852	Down
A116569	LOC_Os03g60560.1	-1.627939478	9.85E-007	Down
A133842	LOC_Os07g38664.1	-1.63278482	2.41E-005	Down
A104386	LOC_Os01g53550.1	-1.634569883	0.00000364	Down
**A126757**	**LOC_Os06g06900.1**	-1.636362825	**3.29E-005**	**Down**
A106741	LOC_Os02g05350.1	-1.636783252	0.000003131	Down
A117856	LOC_Os04g12620.1	-1.642828919	4.49E-006	Down
A105608	LOC_Os01g66670.1	-1.647265603	0.00886553	Down
A123982	LOC_Os05g26490.1	-1.647680733	0.000002369	Down
A130848	LOC_Os07g02940.1	-1.649483985	2.22E-006	Down
A115615	LOC_Os03g49630.1	-1.650547919	0.000001132	Down
A135783	LOC_Os08g11060.1	-1.654010925	3.13E-005	Down
A123999	LOC_Os05g26720.1	-1.655755408	1.13E-006	Down
A138553	LOC_Os08g42750.1	-1.657164906	1.48E-006	Down
A101636	LOC_Os01g19340.1	-1.66168972	0.000006149	Down
A141761	LOC_Os09g37750.1	-1.665428427	0.001821911	Down
A138419	LOC_Os08g41340.1	-1.666306562	5.63E-005	Down
A132716	LOC_Os07g26110.1	-1.667878344	0.000035631	Down
A120416	LOC_Os04g43440.1	-1.668355414	1.23E-005	Down
**A141655**	**LOC_Os09g36730.1**	-1.670433805	**5.08E-006**	**Down**
A101140	LOC_Os01g13360.1	-1.672353786	8.55E-006	Down
**A131172**	**LOC_Os07g06680.1**	-1.676400373	**6.10E-006**	**Down**
A106607	LOC_Os02g04030.1	-1.678981268	0.001055112	Down
**A133517**	**LOC_Os07g35140.1**	-1.680366485	**0.000314007**	**Down**
A120431	LOC_Os04g43660.1	-1.680532924	3.40E-005	Down
A121317	LOC_Os04g53360.1	-1.683244546	1.69E-006	Down
**A123881**	**LOC_Os05g25350.1**	-1.687515836	**0.000001021**	**Down**
A135112	LOC_Os08g03710.1	-1.688373808	3.48E-006	Down
A108341	LOC_Os02g24080.4	-1.690904447	1.79E-005	Down
**A106638**	**LOC_Os02g04330.1**	-1.691052416	**2.30E-006**	**Down**
A126855	LOC_Os06g07923.1	-1.695190916	3.90E-006	Down
A101145	LOC_Os01g13420.1	-1.700113806	1.96E-006	Down
A109402	LOC_Os02g36300.1	-1.700269925	0.000003961	Down
**A100300**	**LOC_Os01g04230.1**	-1.704502708	**3.07E-006**	**Down**
A127047	LOC_Os06g10000.1	-1.705297029	1.06E-006	Down
A110622	LOC_Os02g49410.1	-1.707031344	6.83E-006	Down
A112600	LOC_Os03g12870.1	-1.710967974	0.008353031	Down
A146494	LOC_Os11g14940.1	-1.711148476	0.002233611	Down
A136025	LOC_Os08g14195.1	-1.711820857	6.72E-006	Down
A136103	LOC_Os08g15040.1	-1.717827324	3.87E-006	Down
A107545	LOC_Os02g14500.1	-1.72099214	3.58E-006	Down

**A107762**	**LOC_Os02g16820.1**	**-1.726724206**	**1.83E-006**	**Down**
A145448	LOC_Os11g03280.1	**-1.727326272**	9.93E-006	Down
A104966	LOC_Os01g59640.1	**-1.729830591**	1.71E-005	Down
A142858	LOC_Os10g11580.1	**-1.736762839**	6.45E-006	Down
A122261	LOC_Os05g04700.1	**-1.737685337**	1.17E-006	Down
A116759	LOC_Os03g62580.1	**-1.74386577**	1.71E-005	Down
A105570	LOC_Os01g66210.1	**-1.745557411**	1.76E-005	Down
A126691	LOC_Os06g06230.1	**-1.747729179**	1.69E-006	Down
A132114	LOC_Os07g17330.1	**-1.750695996**	0.000001125	Down
A123965	LOC_Os05g26250.1	**-1.75131525**	5.87E-007	Down
A123976	LOC_Os05g26377.1	**-1.752130139**	6.20E-007	Down
A117122	LOC_Os04g03260.1	**-1.757005699**	0.023392439	Down
A100272	LOC_Os01g03950.1	**-1.762682813**	6.45E-006	Down
**A126946**	**LOC_Os06g08910.1**	**-1.763891707**	**0.000102554**	**Down**
**A107609**	**LOC_Os02g15169.1**	**-1.76457612**	**1.29E-006**	**Down**
A143621	LOC_Os10g23830.1	**-1.768176145**	0.000118183	Down
A146556	LOC_Os11g15630.1	**-1.778543268**	0.000002878	Down
A101794	LOC_Os01g21585.1	**-1.780548887**	2.15E-005	Down
A152707	LOC_Os12g42284.1	**-1.782814337**	5.98E-005	Down
A135337	LOC_Os08g06210.1	**-1.789413273**	7.73E-006	Down
A117160	LOC_Os04g03710.1	**-1.789965462**	0.004029456	Down
A114460	LOC_Os03g36080.1	**-1.791102714**	8.99E-006	Down
A109728	LOC_Os02g39764.1	**-1.801962973**	0.00010402	Down
A107344	LOC_Os02g12410.1	**-1.810278101**	4.48E-005	Down
A130366	LOC_Os06g48320.1	**-1.814622091**	5.62E-007	Down
A141634	LOC_Os09g36500.1	**-1.814901398**	0.00125014	Down
A128333	LOC_Os06g24711.1	**-1.816330826**	4.50E-007	Down
A137978	LOC_Os08g36680.1	**-1.817606506**	1.13E-005	Down
A115664	LOC_Os03g50210.1	**-1.825009948**	2.77E-006	Down
A123974	LOC_Os05g26350.1	**-1.829116336**	0.000001223	Down
A123978	LOC_Os05g26440.1	**-1.83170948**	5.67E-007	Down
A151759	LOC_Os12g32170.1	**-1.835754753**	4.04E-005	Down
A120910	LOC_Os04g48780.1	**-1.836023154**	1.68E-006	Down
A124674	LOC_Os05g34510.1	**-1.837995268**	6.24E-006	Down
A149500	LOC_Os12g04570.1	**-1.839237957**	3.53E-006	Down
A103665	LOC_Os01g45659.1	**-1.843995556**	6.38E-007	Down
A141723	LOC_Os09g37394.1	**-1.849890382**	2.31E-006	Down
**A134089**	**LOC_Os07g41310.1**	**-1.85306866**	**0.00000057**	**Down**
A152269	LOC_Os12g37650.1	**-1.854265405**	8.97E-007	Down
A137463	LOC_Os08g31250.1	**-1.858405292**	3.66E-006	Down
A115228	LOC_Os03g45390.1	**-1.863460657**	5.22E-007	Down
A148266	LOC_Os11g37900.1	**-1.863998589**	3.24E-006	Down
A123990	LOC_Os05g26620.1	**-1.880190391**	4.10E-007	Down
A138007	LOC_Os08g37010.1	**-1.892900857**	0.000000763	Down
A106143	LOC_Os01g72910.1	**-1.899610611**	5.55E-006	Down
A133382	LOC_Os07g33650.1	**-1.901027098**	3.24E-006	Down
A136558	LOC_Os08g20480.1	**-1.902527567**	3.69E-005	Down
A119453	LOC_Os04g32190.1	**-1.910506191**	4.09E-005	Down

A144795	LOC_Os10g38050.1	-1.915522945	1.34E-006	Down
A111631	LOC_Os03g02070.1	-1.91692482	1.29E-006	Down
A109750	LOC_Os02g39970.1	-1.917013364	8.46E-007	Down
A137333	LOC_Os08g29740.1	-1.918126495	1.68E-005	Down
A134687	LOC_Os07g47790.1	-1.923271748	9.42E-006	Down
A124002	LOC_Os05g26750.1	-1.930970891	5.44E-007	Down
A122604	LOC_Os05g08540.1	-1.935957073	1.04E-005	Down
A101143	LOC_Os01g13390.1	-1.93871414	3.96E-007	Down
A101421	LOC_Os01g16430.1	-1.944321698	4.07E-007	Down
A109627	LOC_Os02g38780.1	-1.94477647	1.48E-005	Down
A101206	LOC_Os01g14060.1	-1.949248631	9.26E-007	Down
A109221	LOC_Os02g34270.1	-1.978715615	4.28E-007	Down
A126725	LOC_Os06g06580.1	-1.986964279	2.62E-006	Down
A142097	LOC_Os10g02260.1	-1.993393446	0.002973227	Down
A135653	LOC_Os08g09700.1	-2.001228654	4.97E-006	Down
A123975	LOC_Os05g26368.1	-2.002606496	2.60E-007	Down
**A103587**	**LOC_Os01g44220.1**	**-2.003245019**	**6.92E-007**	**Down**
A134134	LOC_Os07g41810.1	-2.003560951	3.30E-007	Down
A107265	LOC_Os02g11110.1	-2.01042241	5.41E-007	Down
A141215	LOC_Os09g30250.1	-2.029108062	1.31E-005	Down
A148654	LOC_Os11g42210.1	-2.070210112	0.000131007	Down
A109941	LOC_Os02g42450.1	-2.070656247	1.32E-006	Down
A128377	LOC_Os06g25294.1	-2.078393803	2.17E-005	Down
A144514	LOC_Os10g34770.1	-2.083813664	3.38E-006	Down
A119671	LOC_Os04g34510.1	-2.089040049	4.29E-007	Down
**A114172**	**LOC_Os03g31360.1**	**-2.096632272**	**2.35E-007**	**Down**
A116993	LOC_Os04g01780.1	-2.102241965	2.26E-005	Down
A126719	LOC_Os06g06520.1	-2.105536915	6.78E-007	Down
A108854	LOC_Os02g29774.1	-2.108818293	6.37E-007	Down
A128989	LOC_Os06g33640.1	-2.113120714	4.76E-007	Down
A135339	LOC_Os08g06230.1	-2.120538342	3.30E-007	Down
A123019	LOC_Os05g13810.2	-2.123395186	6.51E-007	Down
A116325	LOC_Os03g57880.1	-2.12848422	6.09E-006	Down
A137517	LOC_Os08g31850.1	-2.13029603	9.02E-007	Down
A128993	LOC_Os06g33690.1	-2.136584365	2.34E-006	Down
**A100160**	**LOC_Os01g02730.1**	**-2.136907161**	**1.21E-006**	**Down**
A135356	LOC_Os08g06415.1	-2.150720191	3.17E-007	Down
A152590	LOC_Os12g41110.1	-2.156254119	2.35E-007	Down
A132533	LOC_Os07g23820.1	-2.169407754	0.000000531	Down
A121016	LOC_Os04g49970.1	-2.179477972	5.08E-006	Down
A111756	LOC_Os03g03480.1	-2.183765278	1.78E-006	Down
A133815	LOC_Os07g38400.1	-2.199226462	5.42E-007	Down
**A104587**	**LOC_Os01g55630.1**	**-2.207509693**	**1.80E-007**	**Down**
A124049	LOC_Os05g27340.1	-2.209469374	1.76E-007	Down
**A107601**	**LOC_Os02g15090.1**	**-2.21991306**	**1.50E-007**	**Down**
A100124	LOC_Os01g02334.1	-2.227632755	2.13E-007	Down
A110211	LOC_Os02g45120.1	-2.269350486	1.56E-007	Down
A123981	LOC_Os05g26480.1	-2.278072553	1.90E-007	Down

A144224	LOC_Os10g31420.1	**-2.295671652**	2.93E-007	Down
A107636	LOC_Os02g15460.1	**-2.295685485**	0.000322167	Down
A134431	LOC_Os07g45060.1	**-2.297894616**	1.41E-006	Down
A123964	LOC_Os05g26240.1	**-2.307257933**	0.000000153	Down
A104792	LOC_Os01g57840.1	**-2.321548116**	3.02E-007	Down
A122220	LOC_Os05g04240.1	**-2.327178023**	1.26E-006	Down
A129281	LOC_Os06g36930.1	**-2.358517594**	4.60E-007	Down
A120451	LOC_Os04g43860.1	**-2.364777835**	0.000004315	Down
A102123	LOC_Os01g25450.1	**-2.366057686**	1.82E-006	Down
A108326	LOC_Os02g23930.1	**-2.374781192**	6.21E-005	Down
A106450	LOC_Os02g02410.1	**-2.375140031**	4.27E-007	Down
A136404	LOC_Os08g18770.1	**-2.386961829**	8.41E-007	Down
A135928	LOC_Os08g13050.1	**-2.404491379**	0.000354616	Down
A123980	LOC_Os05g26460.1	**-2.407825978**	9.15E-008	Down
A146466	LOC_Os11g14650.1	**-2.454234**	1.15E-006	Down
A135645	LOC_Os08g09610.1	**-2.486518844**	1.42E-007	Down
A115293	LOC_Os03g46180.1	**-2.496969686**	0.000000216	Down
A121272	LOC_Os04g52890.1	**-2.521113544**	0.000000199	Down
A110580	LOC_Os02g48980.1	**-2.523229292**	1.19E-007	Down
A115696	LOC_Os03g50530.1	**-2.533581869**	0.000000268	Down
A131317	LOC_Os07g08290.1	**-2.539840833**	0.000211848	Down
A100283	LOC_Os01g04050.1	**-2.553087762**	2.25E-007	Down
A144735	LOC_Os10g37350.1	**-2.577591693**	5.19E-006	Down
**A130282**	**LOC_Os06g47470.1**	**-2.592462237**	**5.79E-006**	**Down**
A128259	LOC_Os06g23870.1	**-2.636178076**	1.72E-007	Down
A115375	LOC_Os03g47050.1	**-2.657453314**	5.81E-007	Down
A134394	LOC_Os07g44670.1	**-2.660782561**	1.74E-007	Down
A136687	LOC_Os08g22210.1	**-2.672237408**	3.02E-007	Down
A150482	LOC_Os12g15470.1	**-2.802614242**	2.46E-006	Down
A109039	LOC_Os02g32250.1	**-2.820681539**	0.00000005	Down
A128756	LOC_Os06g30620.1	**-2.852649403**	2.93E-006	Down
A109253	LOC_Os02g34610.1	**-2.852952124**	1.42E-007	Down
A112730	LOC_Os03g14250.1	**-2.854808305**	4.00E-007	Down
A113182	LOC_Os03g19070.1	**-2.876349975**	5.90E-007	Down
A112045	LOC_Os03g06550.1	**-2.888396886**	3.94E-008	Down
A108053	LOC_Os02g19990.1	**-2.912880408**	1.65E-007	Down
A116336	LOC_Os03g57990.1	**-2.961382614**	1.53E-006	Down
A108588	LOC_Os02g26790.1	**-2.984262668**	2.94E-008	Down
A135558	LOC_Os08g08655.1	**-2.995890282**	1.58E-006	Down
A136472	LOC_Os08g19410.1	**-3.004289288**	0.000000369	Down
A148823	LOC_Os11g44014.1	**-3.08402704**	1.46E-006	Down
A143823	LOC_Os10g26320.1	**-3.126809983**	8.70E-007	Down
**A107607**	**LOC_Os02g15150.1**	**-3.160810178**	**3.16E-007**	**Down**
A109871	LOC_Os02g41730.1	**-3.197671176**	7.10E-005	Down
A134549	LOC_Os07g46400.1	**-3.247438817**	4.89E-007	Down
A112980	LOC_Os03g16940.1	**-3.26516394**	9.22E-008	Down
A138112	LOC_Os08g38130.1	**-3.272639893**	1.20E-006	Down
A141733	LOC_Os09g37480.1	**-3.280206287**	7.69E-007	Down

A126104	LOC_Os05g50920.1	**-3.353596348**	1.08E-007	Down
A127505	LOC_Os06g14870.1	**-3.367744453**	0.000180961	Down
**A130569**	**LOC_Os06g50510.1**	**-3.435205377**	**2.23E-006**	**Down**
A111072	LOC_Os02g54040.1	**-3.44713346**	4.31E-008	Down
**A107555**	**LOC_Os02g14600.1**	**-3.491667476**	**2.25E-008**	**Down**
A115843	LOC_Os03g52230.1	**-3.523284363**	2.24E-006	Down
A126324	LOC_Os06g02340.1	**-3.535350311**	3.82E-007	Down
A108169	LOC_Os02g21260.1	**-3.596713156**	1.17E-008	Down
A111670	LOC_Os03g02470.1	**-3.631188831**	1.33E-008	Down
A148523	LOC_Os11g40770.1	**-3.758789683**	0.000000568	Down
A141099	LOC_Os09g28980.1	**-3.977407307**	1.76E-007	Down
A144219	LOC_Os10g31360.1	**-4.164991207**	7.95E-008	Down
A148182	LOC_Os11g36980.1	**-4.312185357**	3.41E-009	Down
**A126494**	**LOC_Os06g04200.1**	**-4.31301831**	**0.000000049**	**Down**
A137582	LOC_Os08g32510.1	**-4.432731038**	5.05E-009	Down
A139662	LOC_Os09g12210.1	**-4.437437015**	3.93E-009	Down
A138510	LOC_Os08g42320.1	**-4.570179685**	4.65E-009	Down
A145596	LOC_Os11g04790.1	**-4.643085862**	0.000000002	Down
A135930	LOC_Os08g13070.1	**-4.695064949**	9.16E-009	Down
A106142	LOC_Os01g72900.1	**-4.745064745**	3.81E-009	Down
A127982	LOC_Os06g20750.1	**-5.03774345**	1.31E-009	Down
A139429	LOC_Os09g09410.1	**-5.052952093**	2.25E-008	Down
A104756	LOC_Os01g57470.1	**-5.097045811**	2.83E-009	Down
A147113	LOC_Os11g24260.1	**-5.320063942**	1.50E-009	Down
A109496	LOC_Os02g37309.1	**-5.925490064**	1.48E-009	Down
A116782	LOC_Os03g62810.1	**-6.596601117**	1.72E-008	Down

Supplementary Table 4. List of DEGs between IR36ae vs IR36 with potential candidate genes in bold.

ProbeName	SystematicName	logFC	P.Value	Regulation
**A131054**	**LOC_Os07g05290.1**	**1.743003642**	**0.005197885**	**Up**
**A133006**	**LOC_Os07g29490.1**	**1.695897836**	**0.000539085**	**Up**
**A143405**	**LOC_Os10g21070.1**	**1.484520945**	**0.036886619**	**Up**
**A137999**	**LOC_Os08g36910.1**	**1.454289975**	**6.35E-006**	**Up**
**A146227**	**LOC_Os11g11480.1**	**1.317165664**	**0.027094185**	**Up**
**A143764**	**LOC_Os10g25630.1**	**1.304909049**	**0.049137169**	**Up**
**A139447**	**LOC_Os09g09610.1**	**1.281422415**	**0.024829376**	**Up**
**A114032**	**LOC_Os03g29560.1**	**1.279986562**	**0.038633498**	**Up**
A107625	LOC_Os02g15340.1	**1.273405608**	0.001151677	**Up**
**A150145**	**LOC_Os12g11620.1**	**1.234236171**	**0.000141834**	**Up**
**A107307**	**LOC_Os02g12020.1**	**1.221317918**	**0.008430321**	**Up**
A112862	LOC_Os03g15680.1	**1.220693517**	0.007616515	**Up**
**A143388**	**LOC_Os10g20870.1**	**1.220048383**	**0.00393148**	**Up**
A147370	LOC_Os11g27400.1	**1.218789668**	0.046242397	**Up**
**A103160**	**LOC_Os01g39510.1**	**1.209032498**	**0.045358642**	**Up**
**A150174**	**LOC_Os12g11990.1**	**1.202590991**	**0.000104316**	**Up**
A103381	LOC_Os01g41930.1	**1.182436837**	0.016158921	**Up**
**A108554**	**LOC_Os02g26430.1**	**1.181126223**	**3.59E-005**	**Up**
**A110869**	**LOC_Os02g51900.1**	**1.171805998**	**0.047975228**	**Up**
**A149864**	**LOC_Os12g08480.1**	**1.150714588**	**0.00561903**	**Up**
**A141044**	**LOC_Os09g28400.1**	**1.135553269**	**1.02E-005**	**Up**
**A105753**	**LOC_Os01g68230.1**	**1.129152787**	**0.028938805**	**Up**
**A135776**	**LOC_Os08g10990.1**	**1.127837849**	**0.03387102**	**Up**
**A150422**	**LOC_Os12g14670.1**	**1.123911942**	**0.001581378**	**Up**
**A122943**	**LOC_Os05g12540.1**	**1.106720463**	**0.012631728**	**Up**
**A121649**	**LOC_Os04g56850.1**	**1.10009006**	**0.024585689**	**Up**
A100950	LOC_Os01g11260.1	**1.097148169**	0.000857306	**Up**
**A117066**	**LOC_Os04g02590.1**	**1.090849518**	**0.00669837**	**Up**
**A130408**	**LOC_Os06g48830.1**	**1.087073246**	**2.20E-005**	**Up**
**A143173**	**LOC_Os10g17920.1**	**1.086217222**	**0.018620254**	**Up**
**A137383**	**LOC_Os08g30340.1**	**1.084777237**	**0.004693517**	**Up**
**A137998**	**LOC_Os08g36900.1**	**1.083110615**	**4.10E-005**	**Up**
**A151046**	**LOC_Os12g23400.1**	**1.0696427**	**0.002433015**	**Up**
**A132454**	**LOC_Os07g22880.1**	**1.068996778**	**0.007852881**	**Up**
**A129138**	**LOC_Os06g35290.1**	**1.066573937**	**0.022911417**	**Up**
**A129554**	**LOC_Os06g39860.1**	**1.058363696**	**0.046309632**	**Up**
**A119041**	**LOC_Os04g27470.1**	**1.05197612**	**0.04362466**	**Up**
**A133642**	**LOC_Os07g36544.2**	**1.049992966**	**0.0082287**	**Up**
A130114	LOC_Os06g45720.1	**1.045067722**	0.005913329	**Up**
**A151245**	**LOC_Os12g25800.1**	**1.038917327**	**0.005181883**	**Up**
A103172	LOC_Os01g39660.1	**1.032823004**	0.059065316	**Up**
**A150571**	**LOC_Os12g16524.1**	**1.032258552**	**0.051583425**	**Up**
**A103774**	**LOC_Os01g46890.1**	**1.020757478**	**0.014957383**	**Up**
A100850	LOC_Os01g10130.1	**1.019162192**	0.018079862	**Up**
A106290	LOC_Os01g74450.1	**1.012309755**	0.000024193	**Up**
**A135780**	**LOC_Os08g11030.1**	**1.009380261**	**0.000252302**	**Up**

A123076	LOC_Os05g14380.1	-1.000026677	0.00425963	Down
A114011	LOC_Os03g29290.1	-1.005208349	0.019005214	Down
A146732	LOC_Os11g18230.1	-1.006715586	0.009479619	Down
A107599	LOC_Os02g15070.1	-1.01127877	0.000116241	Down
A111596	LOC_Os03g01720.1	-1.011664373	0.000559895	Down
A100611	LOC_Os01g07550.1	-1.011920855	0.001034667	Down
A115057	LOC_Os03g43470.1	-1.016911832	0.047706344	Down
A137042	LOC_Os08g26510.1	-1.023345317	0.000706088	Down
A151376	LOC_Os12g27460.1	-1.024301742	0.004819281	Down
A138220	LOC_Os08g39280.1	-1.027188576	0.001963907	Down
A134687	LOC_Os07g47790.1	-1.027204847	6.82E-005	Down
A114570	LOC_Os03g37630.1	-1.028856792	0.046762046	Down
A110291	LOC_Os02g45974.1	-1.031134807	8.01E-005	Down
A134809	LOC_Os07g49080.1	-1.034529165	0.036731179	Down
A143688	LOC_Os10g24670.1	-1.034828939	0.039599998	Down
A147410	LOC_Os11g27880.1	-1.04052635	0.024312322	Down
A131901	LOC_Os07g14640.1	-1.040647192	0.001034429	Down
A143155	LOC_Os10g17724.1	-1.0552393	0.001466707	Down
A138205	LOC_Os08g39130.1	-1.057819213	0.005814266	Down
A118258	LOC_Os04g17880.1	-1.059989358	0.001886941	Down
A118000	LOC_Os04g14400.1	-1.06012202	0.019909678	Down
A138999	LOC_Os09g03490.1	-1.061401107	0.055389171	Down
A102035	LOC_Os01g24490.1	-1.06281747	0.030460521	Down
A148983	LOC_Os11g45880.1	-1.063037552	0.026040611	Down
A134850	LOC_Os07g49490.1	-1.066403941	0.010506709	Down
A110499	LOC_Os02g48140.1	-1.074446254	0.000130257	Down
A129075	LOC_Os06g34620.1	-1.077745188	0.035871693	Down
A118924	LOC_Os04g26110.1	-1.083619722	0.001550513	Down
A111178	LOC_Os02g55160.1	-1.089978341	0.056182626	Down
A126195	LOC_Os05g51910.1	-1.092678784	0.005545836	Down
A138663	LOC_Os08g43930.1	-1.097544776	0.008924399	Down
A122944	LOC_Os05g12550.1	-1.104170975	0.043906496	Down
A125998	LOC_Os05g49840.1	-1.106693167	0.005535953	Down
A110284	LOC_Os02g45900.1	-1.113049586	0.019328796	Down
A127982	LOC_Os06g20750.1	-1.114400805	2.01E-005	Down
A115664	LOC_Os03g50210.1	-1.115253092	3.36E-005	Down
A147299	LOC_Os11g26560.1	-1.12637046	0.008508762	Down
A148903	LOC_Os11g44970.1	-1.136384145	0.051168485	Down
A135058	LOC_Os08g03110.1	-1.138542769	2.64E-005	Down
A148444	LOC_Os11g39820.1	-1.14016213	0.000521626	Down
A111294	LOC_Os02g56470.1	-1.141536595	0.020143218	Down
A133961	LOC_Os07g39910.1	-1.143181925	0.039736617	Down
A130544	LOC_Os06g50240.1	-1.143887437	0.012072563	Down
A149505	LOC_Os12g04630.1	-1.151477017	5.18E-005	Down
A149803	LOC_Os12g07870.1	-1.152285884	1.20E-005	Down
A126685	LOC_Os06g06170.1	-1.152367035	0.004683886	Down
A140048	LOC_Os09g16570.1	-1.162352364	0.010197757	Down
A107924	LOC_Os02g18530.1	-1.163719942	0.022550746	Down

A152796	LOC_Os12g43300.1	-1.175990941	0.040347743	Down
A114355	LOC_Os03g33610.1	-1.177581753	0.010614174	Down
A152313	LOC_Os12g38160.1	-1.183945028	0.047442272	Down
A114632	LOC_Os03g38320.1	-1.184716137	0.029478803	Down
A146403	LOC_Os11g13980.1	-1.185038471	1.11E-005	Down
A120508	LOC_Os04g44470.1	-1.187925028	1.64E-005	Down
A135660	LOC_Os08g09760.1	-1.188484809	0.030631781	Down
A131833	LOC_Os07g13910.1	-1.201923034	0.048834261	Down
A150673	LOC_Os12g17780.1	-1.217458501	0.050377937	Down
A108403	LOC_Os02g24800.1	-1.219555163	0.028246283	Down
A122580	LOC_Os05g08300.1	-1.219850756	0.000936091	Down
A117355	LOC_Os04g06244.1	-1.221664645	0.049133532	Down
A112327	LOC_Os03g09990.1	-1.224709676	0.003521659	Down
A150461	LOC_Os12g15180.1	-1.23042099	0.043355736	Down
A149117	LOC_Os11g47630.1	-1.232850722	0.000323976	Down
A150659	LOC_Os12g17590.1	-1.235567228	0.007696265	Down
A117001	LOC_Os04g01860.1	-1.242599241	0.044599372	Down
A148196	LOC_Os11g37130.1	-1.24903391	0.000010184	Down
A111895	LOC_Os03g04990.1	-1.25271124	0.008060517	Down
A102366	LOC_Os01g28830.1	-1.265300352	0.056245628	Down
A128300	LOC_Os06g24300.1	-1.269651384	0.04786808	Down
A139600	LOC_Os09g11460.1	-1.271499329	0.001683362	Down
A124596	LOC_Os05g33690.1	-1.274345951	0.027222907	Down
A135081	LOC_Os08g03410.1	-1.306865189	8.75E-006	Down
A151234	LOC_Os12g25665.1	-1.313758438	0.018629372	Down
A129057	LOC_Os06g34450.1	-1.335556393	1.52E-005	Down
A146588	LOC_Os11g16440.1	-1.349077259	0.00548793	Down
A117786	LOC_Os04g11820.1	-1.349463422	0.00295974	Down
A145845	LOC_Os11g07300.1	-1.364382849	0.001384422	Down
A124638	LOC_Os05g34120.1	-1.369529323	0.006682928	Down
A151485	LOC_Os12g28870.1	-1.410498985	0.043405596	Down
A103337	LOC_Os01g41470.1	-1.420433168	0.013884691	Down
A110771	LOC_Os02g50900.1	-1.436015874	0.000538835	Down
A143749	LOC_Os10g25430.1	-1.437494817	2.81E-005	Down
A147354	LOC_Os11g27150.1	-1.451997823	0.009662736	Down
A124845	LOC_Os05g36330.1	-1.466318694	1.75E-005	Down
A101794	LOC_Os01g21585.1	-1.475298774	5.46E-005	Down
A136226	LOC_Os08g16310.1	-1.536733593	0.036846153	Down
A115218	LOC_Os03g45280.1	-1.547142583	4.07E-006	Down
A151575	LOC_Os12g29970.1	-1.586478734	0.000006534	Down
A144224	LOC_Os10g31420.1	-1.641097986	1.33E-005	Down
A103496	LOC_Os01g43200.1	-1.641254493	0.001443033	Down
A113909	LOC_Os03g27710.1	-1.655369753	2.90E-005	Down
A100556	LOC_Os01g06980.1	-1.700044503	0.056263749	Down
A115293	LOC_Os03g46180.1	-1.748475695	1.43E-006	Down
A116557	LOC_Os03g60440.1	-1.790421206	0.000566954	Down
A150350	LOC_Os12g13850.1	-1.866766275	0.000126113	Down
A112003	LOC_Os03g06060.1	-2.004989084	0.000002995	Down

**A120910**	**LOC_Os04g48780.1**	**-2.063417568**	**2.61E-006**	**Down**
A143621	LOC_Os10g23830.1	**-2.073971994**	9.85E-006	**Down**
**A124523**	**LOC_Os05g32950.1**	**-2.200538552**	**6.89E-005**	**Down**
**A119453**	**LOC_Os04g32190.1**	**-2.23285683**	**2.90E-007**	**Down**
**A112002**	**LOC_Os03g06050.1**	**-2.310859158**	**2.84E-007**	**Down**
**A131317**	**LOC_Os07g08290.1**	**-2.417099145**	**1.05E-006**	**Down**
**A129281**	**LOC_Os06g36930.1**	**-2.441444474**	**4.29E-007**	**Down**
A104966	LOC_Os01g59640.1	**-2.458939685**	7.34E-007	**Down**
A150482	LOC_Os12g15470.1	**-2.577914297**	2.11E-005	**Down**
**A143823**	**LOC_Os10g26320.1**	**-3.134156056**	**6.64E-007**	**Down**
A126104	LOC_Os05g50920.1	**-3.5293838**	1.34E-006	**Down**
**A111072**	**LOC_Os02g54040.1**	**-3.640839958**	**7.56E-008**	**Down**
